# Uncovering the Immune Cell Infiltration Landscape in Low-Grade Glioma for Aiding Immunotherapy

**DOI:** 10.1155/2022/3370727

**Published:** 2022-03-11

**Authors:** Youyuan Yang, Yue Tian, Qing Li, Rui Jiang, Junfeng Zhang

**Affiliations:** ^1^Outpatient Department, Sichuan Provincial Military Command of PLA, Chengdu, Sichuan, China; ^2^Department of General Surgery, Daping Hospital, Army Medical University, Chongqing, China; ^3^Department of Oncology, Daping Hospital, Army Medical University, Chongqing, China; ^4^Department of Radiology, The General Hospital of Western Theater Command, Chengdu, Sichuan, China

## Abstract

**Objective:**

Low-grade glioma (LGG) mainly threatens the elderly population, with undesirable prognoses. This study uncovered the immune cell infiltration (ICI) landscape in LGG.

**Methods:**

RNA-seq profiles of LGG were retrieved from TCGA and CGGA databases. CIBERSORTx and ESTIMATE algorithms were employed to characterize the ICI landscape in LGG tissues. Through unsupervised clustering analysis, ICI subtypes were clustered. ICI scores were computed via principal component analysis (PCA). The differences in survival, tumor-infiltrating immune cells, stromal scores, immune scores, immune checkpoint genes, immune activity genes, and tumor mutation burden (TMB) were assessed between high and low ICI score groups.

**Results:**

Three ICI subtypes were constructed in LGG, with distinct survival outcomes, PD-L1 expression, and infiltration levels of immune cells. Furthermore, ICI scores were developed. Both in TCGA and CGGA datasets, low ICI scores were indicative of undesirable outcomes. High ICI scores were significantly correlated to increased infiltration levels of memory B cells, CD8 T cells, CD4 naïve T cells, T follicular helper cells, macrophages M0, and eosinophils, while low ICI scores were characterized by increased infiltration levels of naïve B cells, plasma cells, CD4 memory resting T cells, Tregs, resting NK cells, macrophages M2, and activated dendritic cells. High ICI scores exhibited correlations with lower immune activity genes and immune checkpoint genes. Furthermore, TMB was distinctly reduced in the high ICI score group.

**Conclusion:**

The ICI scores may serve as a promising prognostic index and predictive indicator for immunotherapies, extending our understanding of immune microenvironment in LGG.

## 1. Introduction

Glioma is a common primary intracranial malignancy, which is classified into four grades according to the 2007 WHO classification of tumors: Grades I and II are LGGs, and Grades III and IV are high-grade gliomas [1]. Among them, LGG represents the most common primary brain malignancy [2]. LGG mainly occurs in old people. However, it is predisposed to younger individuals (average age: 41 years old), with mean survival time of approximately seven years [3]. Despite the much progress in neurosurgical resection, chemotherapy, and radiotherapy, it is ineluctable to experience resistance and recurrence [4]. Due to biological behaviors, this malignancy displays considerable heterogeneity. Some subjects experience indolent outcomes, while others develop into high-grade gliomas with undesirable outcomes [5]. Despite the less aggressiveness of LGG, patients usually have varied survival outcomes [6]. Therefore, discovering precisely novel markers to predict patients' prognosis is of importance in current studies.

Immunotherapies have exhibited considerable promise in cancer therapy [7]. Novel immunotherapy has emerged as a promising therapeutic strategy against LGG [8]. Nevertheless, only some patients respond to immunotherapy [9]. The efficacy of immunotherapy is partly affected by tumor microenvironment that contains immune cells as well as stromal cells. Tumor-infiltrating immune cells may affect response to immunotherapies and survival outcomes [10]. Uncovering the relationships between tumor and tumor immune microenvironment is of importance for discovering prognostic markers, lowering drug resistance, and exploiting novel therapeutic strategies [11]. Therefore, it is of significance to construct ICI subtypes to differentiate LGG patients' prognosis. Herein, this study developed ICI score system to characterize the ICI landscape in LGG, which may accurately predict patients' outcomes as well as responsiveness to immunotherapies.

## 2. Materials and Methods

### 2.1. LGG Datasets

RNA-seq data and matched clinical information of LGG patients were retrieved from The Cancer Genome Atlas (TCGA; https://portal.gdc.cancer.gov/) database. After removing samples with survival time of 0, 506 samples were retained as the training set. Furthermore, 596 LGG subjects were obtained from the Chinese Glioma Genome Atlas (CGGA; https://www.cgga.org.cn/), and they were utilized as the validation set.  Table 1 lists the clinical information of the two datasets. Fragments per kilobase of transcript per million fragments mapped (FPKM) values were downloaded from TCGA or CGGA database and transformed into transcripts per kilobase million (TPM) values.

### 2.2. Inferring Tumor-Infiltrating Immune Cells and Stromal Cells

CIBERSORTx algorithm (https://cibersortx.stanford.edu/) was applied to estimate the abundances of immune cells in each LGG sample based on gene expression profiles [12]. The LM22 signatures were employed and permutations were set as 1,000 times. Meanwhile, immune scores and stromal scores were determined to infer the fractions of stromal cells and immune cells in each specimen according to expression data via ESTIMATE algorithm (https://sourceforge.net/projects/estimateproject/) [13].

### 2.3. Unsupervised Clustering Analysis

Consensus clustering method may provide quantitative evidence for determining the number and membership of possible clusters in a dataset. LGG specimens were clustered utilizing “ConsensusClusterPlus” R package (version 1.58.0) [14]. When *k* = 2 to 9, consensus matrix, consensus cumulative distribution function (CDF), delta area, and tracking plots were constructed to determine the optimal *k* value. Then, cluster consensus and item consensus were calculated, respectively. Clustering results were validated by principal component analysis (PCA).

### 2.4. Differential Expression Analysis

Differentially expressed genes (DEGs) with |fold change (FC)| > 2 and adjusted *p* value < 0.05 were screened among ICI subtypes through applying “limma” R package (version 1.9.6) [13].

### 2.5. ICI Scores

Unsupervised clustering analysis was applied for categorizing all subjects based on DEGs. Furthermore, DEGs that displayed positive and negative correlations to gene clusters were separately named as ICI gene signatures A and B. To lower the noise or redundant genes, the Boruta algorithm was utilized for performing dimension reduction in the ICI gene signatures A and B. Principal component 1 (PC1) was extracted as the signature score through applying the PCA. According to previous studies [15, 16], the ICI score of each subject was calculated as follows: ICI score = Ʃ PC1_A_ – PC1_B_.

### 2.6. Functional Enrichment Analysis

Gene ontology (GO) and Kyoto Encyclopedia of Genes and Genomes (KEGG) enrichment analyses of ICI gene signatures A and B were separately presented via “clusterProfiler” R package (version 2.2.7) [17]. Adjusted *p* value < 0.05 indicated significant enrichment.

### 2.7. Gene Set Enrichment Analysis (GSEA)

GSEA, a computational method, may be utilized for determining whether a set of genes display differential expression in two biological states [18]. Here, this study employed GSEA to identify differences in KEGG pathways between high and low ICI score groups. Gene set permutations were presented 1000 times. ICI score was set as a phenotype label. Enriched KEGG pathways were screened based on false discovery rate (FDR) < 0.05.

### 2.8. Tumor Mutation Burden (TMB)

TMB was defined as the ratio of total count of variants and the total length of exons [19]. The differences in TMB between high and low ICI score groups were compared by the Wilcoxon rank-sum test. The correlation coefficient between ICI scores and TMB was computed via Spearman analysis.

### 2.9. Screening Small Molecule Drugs

DEGs with |FC| > 2 and adjusted *p* value < 0.05 were filtered between high and low ICI score groups utilizing “limma” R package. The two lists of upregulated and downregulated genes were analyzed through the Connectivity Map (CMap; http://portals.broadinstitute.org/cmap/) database [20]. Small molecular drugs were filtered based on the enrichment value and permutation *p* value. CMap mode-of-action (MoA) analysis was applied for exploring underlying mechanisms of action.

### 2.10. Statistical Analysis

Statistical analysis was achieved via R language. Kaplan-Meier curves of overall survival (OS) were presented for LGG patients in different subgroups and the differences were compared by log-rank test. Spearman analysis was employed to determine the correlation coefficients. Kruskal-Wallis test was applied for comparing over two subgroups, while Wilcoxon test was utilized for comparing two subgroups. The X-tile software was employed for classifying patients into high and low ICI groups to lower the computational batch effects. Two-tailed *p* value < 0.05 indicated statistical significance.

## 3. Results

### 3.1. Characterization of ICI Subtypes with Distinct Survival Outcomes in LGG

**Figure 1:**
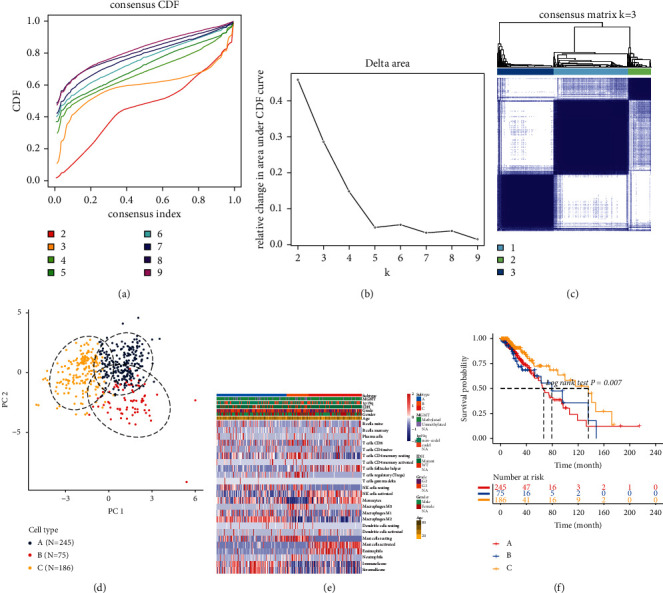
Characterization of ICI subtypes with distinct survival outcomes for LGG in TCGA-LGG dataset. ((a)–(c)) Unsupervised clustering analysis for classifying three ICI subtypes by the “ConsensusClusterPlus” package. (a) Consensus cumulative distribution function graph. (b) Delta area plot. (c) Heatmap for consensus matrix when *k* = 3. (d) PCA plots for the classification patterns of the ICI subtypes. (e) Heatmap of tumor-infiltrating immune cells in different clinical phenotypes and ICI subtypes. (f) Kaplan-Meier curves for OS of LGG patients in the three ICI subtypes.

Here, the CIBERSORTx and ESTIMATE algorithms were employed for determining the infiltration levels of immune cells in LGG tissues. On the basis of 506 LGG specimens plus corresponding ICI profiling, these subjects were classified into three subtypes through the “ConsensusClusterPlus” package (Figures 1(a)–1(c)). PCA results confirmed the distinct classifications into three subtypes: ICI subtype A (*n* = 245), ICI subtype B (*n* = 75), and ICI subtype C (*n* = 186; Figure 1(d)). We further clarified the differences in clinical phenotypes among the three ICI subtypes, as shown in Figure 1(e). Novel immunotherapies have brought hope to LGG patients, but not each patient can respond to such therapies [21]. Since every tumor is different, it is important to investigate how to use the biology behind tumor cells to successfully treat more cancer patients [22]. “Cold” tumors with few T cells are generally less sensitive to immunotherapy [23]. Among the three ICI subtypes, ICI subtype A displayed the lowest infiltration levels of T cells (Figure 1(e)). Moreover, ICI subtype A was in relation with undesirable survival outcomes (*p*=0.007; Figure 1(f)). This classification pattern was confirmed in the CGGA-LGG dataset (Supplementary Figure 1).

### 3.2. The Landscape of Tumor Microenvironment Components in the Three ICI Subtypes of LGG

**Figure 2:**
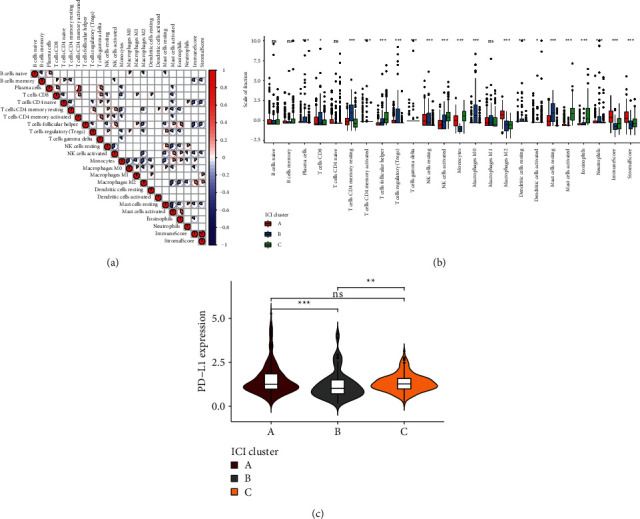
The landscape of tumor microenvironment components in the three ICI subtypes of LGG. (a) Correlations between tumor-infiltrating immune cells, immune scores, and stromal scores in LGG tissues. The more towards red, the greater the positive correlation coefficient; the more towards blue, the greater the negative correlation coefficient. (b) Box plots for the infiltration levels of tumor-infiltrating immune cells in each ICI subtype. (c) Violin plots for the expression of PD-L1 in each ICI subtype. Kruskal-Wallis test, ns: not significant; ^*∗*^*p* < 0.05; ^*∗∗*^*p* < 0.01; ^*∗∗∗*^*p* < 0.001.

The interactions between tumor-infiltrating immune cells, immune scores, and stromal scores in tumor microenvironment of LGG tissues were analyzed in depth.  Figure 2(a) depicts the correlation coefficients between them in tumor microenvironment. We found that activated CD4 memory T cells were strongly positively correlated to plasma cells. Meanwhile, there was a strongly positive correlation between stromal scores and immune scores. ICI subtype B was characterized by increased infiltration levels of plasma cells, CD8 T cells, CD4 memory resting T cells, regulatory T cells (Tregs), macrophages M0, resting dendritic cells, resting mast cells, and neutrophils (Figure 2(b)). ICI subtype C had the features of increased infiltration levels of follicular helper T cells, activated NK cells, monocytes, activated mast cells, and eosinophils. Furthermore, ICI subtype A exhibited the characteristics of elevated macrophage M2 levels, immune scores, and stromal scores. PD-L1, as an immune inhibitory receptor ligand, induces T cell dysfunction as well as apoptosis, thereby suppressing inflammatory responses and promoting tumor immune evasion [24]. Here, the expression of immune checkpoint PD-L1 was evaluated in each ICI subtype. Our results showed that ICI subtype A had the features of an increased PD-L1 expression, while ICI subtype C had the features of decreased PD-L1 expression (Figure 2(c)).

### 3.3. Identifying ICI Gene Clusters for LGG

**Figure 3:**
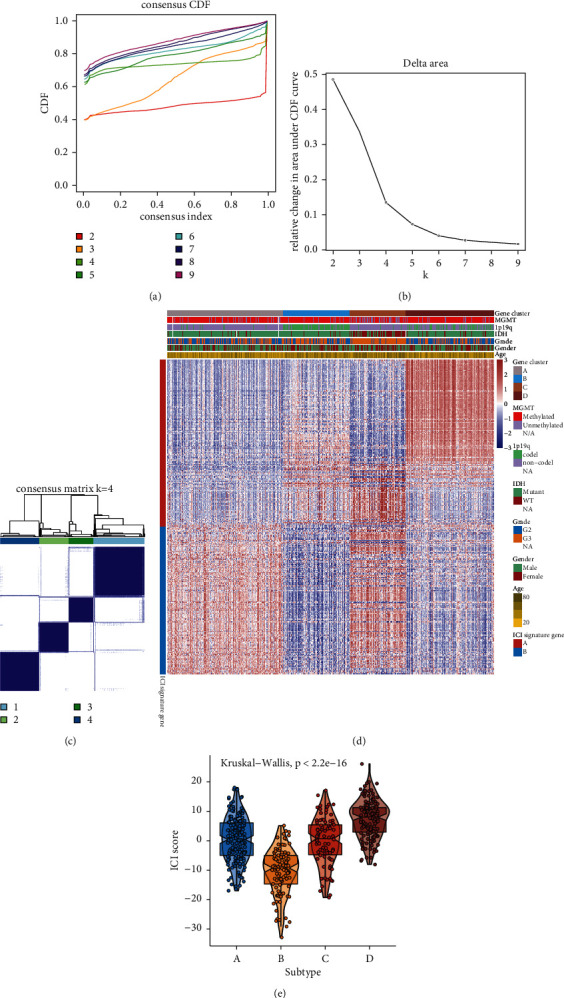
Construction of ICI gene clusters for LGG. ((a)–(c)) Unsupervised clustering analysis for identifying ICI gene clusters based on DEGs among ICI subtypes. (d) Heatmap for clinical features and expression patterns of ICI gene signatures in each ICI gene cluster. (e) Violin plots for the ICI scores in each ICI gene cluster. Kruskal-Wallis test, *p* < 2.2*e* − 16.

This study unraveled potential biological features of the three ICI subtypes. By differential analysis among subtypes, DEGs were determined. Through unsupervised clustering analysis, four ICI gene subtypes were clustered based on these DEGs, called gene clusters A, B, C, and D (Figures 3(a)–3(c)). 231 DEGs that had positive correlations to ICI gene subtypes were called ICI gene signatures A, while 236 DEGs were named as ICI gene signatures B (Supplementary Table 1). The heatmap depicted the clinical features and expression patterns of ICI gene signatures of the four ICI gene clusters (Figure 3(d)). The ICI scores were compared among the gene clusters. We found that gene cluster B was characterized by decreased ICI scores, while gene cluster D had increased ICI scores (*p* < 2.2*e* − 16; Figure 3(e)).

### 3.4. Biological Characteristics of ICI-Relevant Gene Signatures

**Figure 4:**
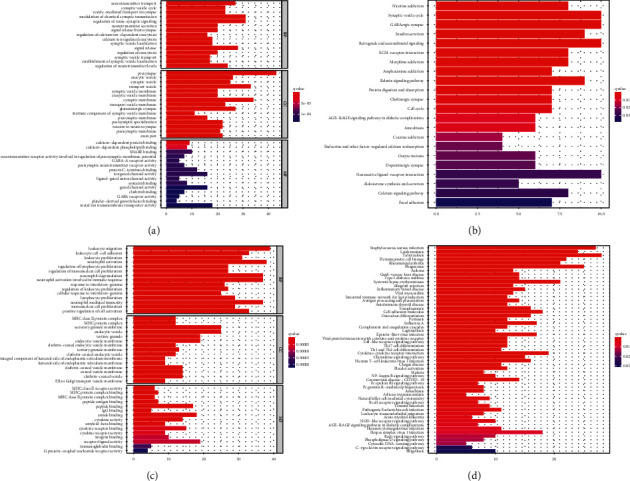
Functional enrichment analysis of ICI gene signatures A and B; GO and KEGG pathway enrichment results of ((a) and (b)) ICI gene signatures A and ((c) and (d)) ICI gene signatures B.

To uncover the biological characteristics of ICI gene signatures A and B, we presented functional enrichment analysis. Our results revealed that ICI gene signatures A were mainly related to signal transduction-related biological processes and pathways such as neurotransmitter transport, synaptic vesicle cycle, vesicle-mediated transport in synapse, modulation of chemical synaptic transmission, regulation of transsynaptic signaling, neurotransmitter secretion, GABAergic synapse, cholinergic synapse, and neuroactive ligand receptor interaction (Figures 4(a) and 4(b)). Meanwhile, ICI gene signatures B were mainly enriched in immune-related pathways such as leukocyte migration, leukocyte cell-cell adhesion, leukocyte proliferation, neutrophil activation, regulation of lymphocyte proliferation, regulation of mononuclear cell proliferation, antigen processing and presentation, complement and coagulation cascades, Toll-like receptor signaling pathway, Th17 cell differentiation, Th1 and Th2 cell differentiation, cytokine-cytokine receptor interaction, and chemokine signaling pathway (Figures 4(c) and 4(d)).

### 3.5. Development of the ICI Score System for LGG

**Figure 5:**
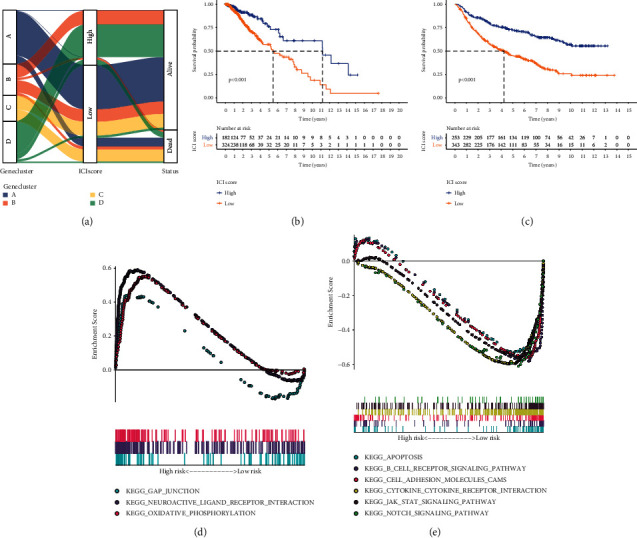
Development of ICI score system for LGG. (a) Alluvial diagram for the distributions of ICI scores and survival status in the four ICI gene clusters. ((b) and (c)) Kaplan-Meier curves of OS for patients with high and low ICI scores in the (b) TCGA-LGG and (c) CGGA-LGG datasets. Log-rank test, *p* < 0.001. ((d) and (e)) GSEA for the enrichment results in (d) high and (e) low ICI score groups.

Based on ICI gene signatures A and B, PCA was presented for computing ICI score of each LGG patient. All patients in the TCGA-LGG dataset were separated into high or low ICI scores according to the optimal cutoff value.  Figure 5(a) depicts the distribution of ICI scores and survival status for patients in the four ICI gene clusters. Patients with low ICI scores exhibited an undesirable prognosis compared to those with high ICI scores in the TCGA-LGG dataset (*p* < 0.001; Figure 5(b)). The prognostic efficiency of the ICI score system was confirmed in the CGGA-LGG dataset (*p* < 0.001; Figure 5(c)). To uncover the biological implications of ICI scores, GSEA was presented. High ICI scores were distinctly correlated to gap junction, neuroactive ligand receptor interaction, and oxidative phosphorylation (Figure 5(d); Supplementary Table 2). Meanwhile, low ICI scores were in relation with apoptosis, B cell receptor signaling pathway, cell adhesion, cytokine-cytokine receptor interaction, JAK STAT signaling pathway, and Notch signaling pathway (Figure 5(e); Supplementary Table 3).

### 3.6. The Roles of ICI Score in Predicting Response to Immunotherapy

**Figure 6:**
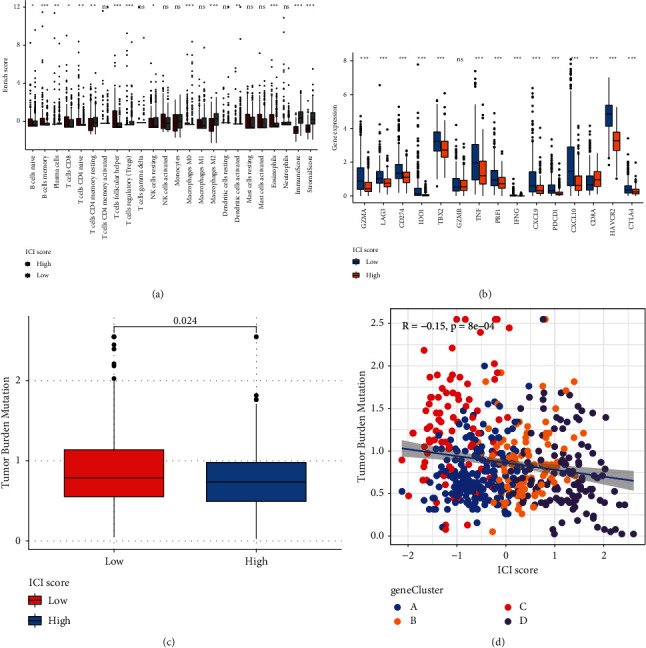
Assessment of the roles of ICI score in predicting response to immunotherapy. (a) The correlations between ICI scores and tumor-infiltrating immune cells. (b) The correlations of ICI scores with immune checkpoint genes and immune activity genes. (c) The TMB levels in high and low ICI score groups. (d) Scatter plots for the Spearman correlation between TMB and ICI scores. Wilcoxon test, ns: not significant; ^*∗*^*p* < 0.05; ^*∗∗*^*p* < 0.01; ^*∗∗∗*^*p* < 0.001.

High ICI scores were significantly correlated to increased infiltration levels of memory B cells, CD8 T cells, CD4 naïve T cells, follicular helper T cells, macrophages M0, and eosinophils (Figure 6(a)). Meanwhile, low ICI scores were characterized by increased infiltration levels of naïve B cells, plasma cells, CD4 memory resting T cells, Tregs, resting NK cells, macrophages M2, and activated dendritic cells as well as increased immune scores and stromal scores. We also assessed the differences in the expression of immune checkpoint genes and immune activity genes between groups. As shown in Figure 6(b), high ICI scores exhibited correlations with lower immune activity genes including GZMA, TBX2, TNF, PRF1, IFNG, CXCL9, and CXCL10 as well as reduced immune checkpoint genes including LAG3, CD274, IDO1, PDCD1, HAVCR2, and CTLA4. Furthermore, TMB score was distinctly reduced in the high ICI score group compared to the low ICI score group (*p*=0.024; Figure 6(c)). Spearman analysis demonstrated that ICI scores displayed a significant negative correlation to TMB (*r* = −0.15, *p*=8*e* − 04; Figure 6(d)). These data indicated that LGG patients with high ICI scores had lower responses to immunotherapy.

### 3.7. Potential Small Molecular Drugs Based on ICI Scores

**Figure 7:**
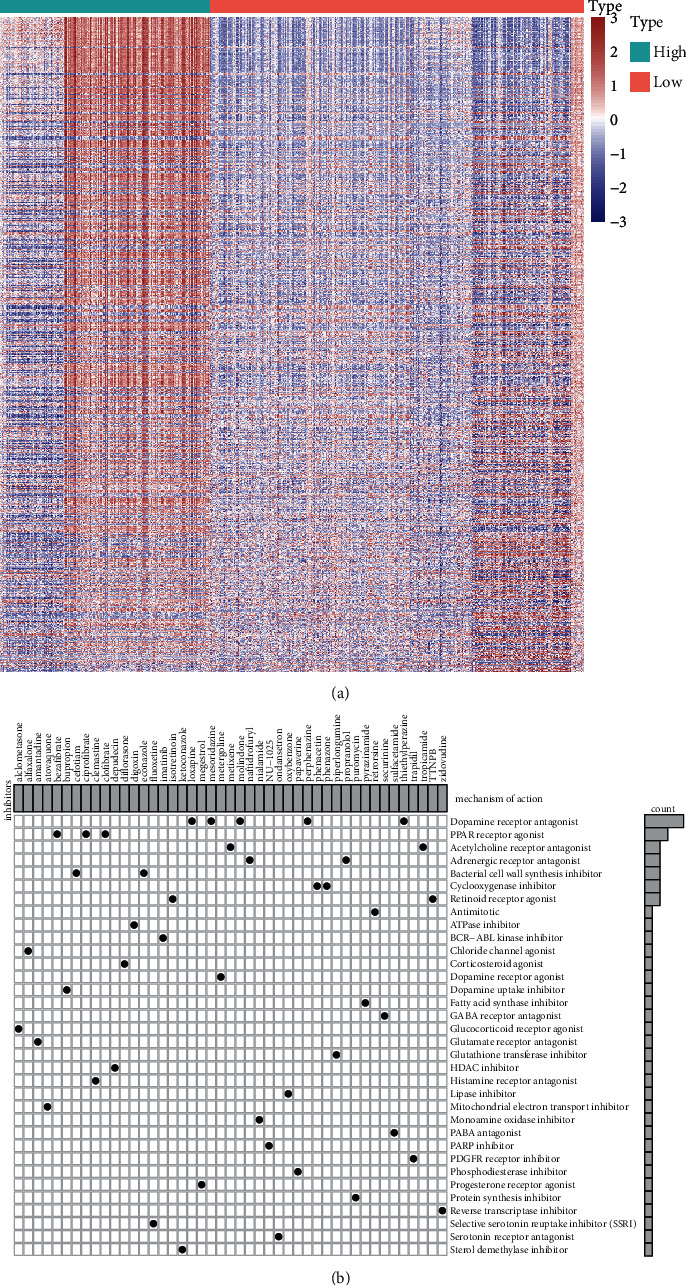
Prediction of potential small molecular drugs based on ICI scores by the CMap database. (a) Heatmap for upregulated genes (red) and downregulated genes (blue) between high and low ICI score groups. (b) Mechanisms of action shared by small molecular compounds.

Small molecular drugs were further predicted by employing the CMap database. Firstly, we identified 775 downregulated genes and 366 upregulated genes in high ICI score group compared to low ICI score group (Figure 7(a); Supplementary Table 4). Through the CMap database, underlying small molecular compounds against LGG such as carbarsone, sulfabenzamide, and phenazone were predicted based on downregulated and upregulated genes, listed in Table 2. Furthermore, the potential mechanisms of action were analyzed via MoA. Dopamine receptor antagonist and PPAR receptor agonist were the most shared mechanisms of action (Figure 7(b)).

## 4. Discussion

LGG displays great heterogeneity at the genetic and molecular levels, affecting the efficacy of immunotherapies [25]. The immune microenvironment of LGG is a complex neuroinflammatory network, involving positive as well as negative immune regulators [26]. This study characterized the ICI landscape and developed ICI score system that may predict survival outcomes as well as the response to immunotherapies, which extended our comprehension about the immune microenvironment of LGG.

Immunohistochemistry and flow cytometry are two commonly applied methods to detect tumor-infiltrating immune cells, depending on a certain biomarker [27–29]. However, because many marker proteins are expressed in distinct cell types, both are misleading and incomplete [30]. Here, we analyzed the fractions of 22 immune cells, immune scores, and stromal scores in LGG tissues by the CIBERSORTx and ESTIMATE algorithms [31]. We found that there was a strongly positive correlation between stromal scores and immune scores. Immune cells and stromal cells are key components in the tumor microenvironment [32], which exert a critical role in LGG progress and survival outcomes [33]. Previously, immune scores and stromal scores exhibited correlations to tumor grade as well as outcomes in LGG [3]. Here, this study characterized three ICI subtypes with distinct survival outcomes and infiltrations of immune cells. ICI subtype B was characterized by increased infiltration levels of plasma cells, CD8 T cells, CD4 memory resting T cells, Tregs, macrophages M0, resting dendritic cells, resting mast cells, and neutrophils. ICI subtype C was featured by increased infiltration levels of follicular helper T cells, activated NK cells, monocytes, activated mast cells, and eosinophils. ICI subtype A exhibited the characteristics of elevated macrophage M2 levels, immune scores, and stromal scores. PD-L1 expression is a critical marker for predicting response to immune checkpoint inhibitor therapy [34]. We found that three ICI subtypes showed correlations to PD-L1 expression, indicating that subjects in the three subtypes could be differentiated to the response to immunotherapy.

This study constructed four ICI gene subtypes. Gene cluster B displayed the features of decreased ICI scores, while gene cluster D had the features of increased ICI scores. We further uncovered the biological characteristics of ICI gene signatures A and B. ICI gene signatures A were mainly related to signal transduction. Malfunction of signal transduction may induce LGG initiation [35]. Moreover, ICI gene signatures B were primarily enriched in immune-related pathways such as Toll-like receptor pathway, chemokine pathway, B cell receptor pathway, and Th1, Th2, and Th17 cell differentiation. ICI score system was developed for prediction of LGG patients' prognosis. Our results showed that patients with low ICI scores exhibited undesirable clinical outcomes, which were confirmed in the CGGA-LGG dataset. Thus, this score system could be utilized for predicting LGG patients' prognosis. We further probed into the biological features based on ICI scores. High ICI scores displayed correlations to gap junction, neuroactive ligand receptor interaction, and oxidative phosphorylation. Furthermore, low ICI scores were significantly related to apoptosis, B cell receptor signaling pathway, cell adhesion, cytokine-cytokine receptor interaction, JAK STAT signaling pathway, and Notch signaling pathway. The above pathways may contribute to LGG progression. For example, IFN-*γ* may activate JAK/STAT pathway by binding to receptor, thereby inducing PD-L1 expression on tumor cells [24]. Several tumor-suppressive factors containing cytokines like TGF-*β* and IL-10 have been discovered in LGG [36].

Immunotherapies have emerged as promising therapeutic strategies in LGG. Tumor-infiltrating immune cells affect responsiveness to such therapies as well as outcomes. Thus, we further characterized the infiltration levels of tumor-infiltrating immune cells in high and low ICI score LGG samples. High ICI scores exhibited correlations to increased infiltration levels of memory B cells, CD8 T cells, CD4 naïve T cells, follicular helper T cells, macrophages M0, and eosinophils, while low ICI scores were in relation with increased infiltration levels of naïve B cells, plasma cells, CD4 memory resting T cells, Tregs, resting NK cells, macrophages M2, and activated dendritic cells. Furthermore, LGG with low ICI scores had increased immune scores as well as stromal scores. These data reflected the heterogeneity of tumor immune microenvironment between high and low ICI score LGG tissues. Immunotherapies with blockage of immune checkpoints have displayed clinical efficacy in LGG [37]. Here, high ICI scores were characterized by decreased immune activity genes including GZMA, TBX2, TNF, PRF1, IFNG, CXCL9, and CXCL10 as well as reduced immune checkpoint genes including LAG3, CD274, IDO1, PDCD1, HAVCR2, and CTLA4. TMB has been an independent prognostic index for glioma and increased TMB indicates poorer survival outcomes [38]. Furthermore, TMB may predict the response to immune checkpoint inhibitors in advanced cancers [39]. In the high ICI score group, there was a reduced TMB score compared to the low ICI score group. Also, ICI score displayed a negative correlation to TMB score. Hence, LGG patients with high ICI scores might have less response to immunotherapies. Based on DEGs between high and low ICI scores, we predicted several small molecular drugs against LGG such as carbarsone, sulfabenzamide, and phenazone. More experiments should be presented to verify the effects of these compounds on treating LGG in future studies.

However, several limitations should be pointed out. First of all, our conclusion was acquired in public databases. Thus, it is indispensable to verify it through experiments. The clinical significance of ICI score in predicting prognosis and immunotherapy in LGG should be confirmed in the future. Despite these limitations, our study provides clues for the ICI landscape in LGG for aiding immunotherapy.

## 5. Conclusion

Collectively, this study characterized the ICI landscape in LGG by the CIBERSORTx and ESTIMATE algorithms. Through unsupervised clustering analysis, we established three ICI subtypes and four ICI gene clusters. PCA was applied to develop ICI score system for LGG. Patients with high ICI scores exhibited favorable clinical outcomes but lower sensitivity to immunotherapies. Despite this, this scoring system should be validated in larger LGG cohorts.

## Figures and Tables

**Table 1:** The clinical characteristics of LGG patients in TCGA and CGGA datasets.

Characteristics	TCGA (*n* = 506)	CGGA (*n* = 596)
Age		
≤50	352	501
>50	154	94
NA	0	1
Gender		
Female	226	251
Male	280	345
IDH		
Mutant	405	416
Wild-type	94	141
NA	7	39
1p19q		
Codel	165	180
Noncodel	337	373
NA	4	43
MGMT		
Methylated	416	287
Unmethylated	86	202
NA	4	107

**Table 2:** Potential small molecular drugs based on ICI scores through the CMap database.

Rank		CMap name	Mean	*n*	Enrichment	*p*	Specificity	Percent nonnull
1		Carbarsone	0.345	4	0.863	0.00048	0	50
2		Sulfabenzamide	0.312	4	0.8	0.00302	0.0072	50
3		Phenazone	−0.359	3	−0.838	0.00853	0.0173	66
4		Prestwick-675	−0.468	4	−0.743	0.00869	0.0928	75
5		Epitiostanol	−0.386	4	−0.712	0.0141	0.0432	50
6		Cinoxacin	−0.5	4	−0.701	0.01675	0.0197	75
7		Econazole	0.508	4	0.7	0.01677	0.1282	75
8		Betulin	0.456	3	0.771	0.02396	0.0127	66
9		Mevalolactone	−0.443	3	−0.77	0.02504	0.0514	66
10		Depudecin	0.391	2	0.887	0.0263	0.0188	50
11	Antazoline		−0.433	4	−0.658	0.03127	0.0315	75
12	16,16-Dimethylprostaglandin E2		−0.388	3	−0.749	0.03219	0.0276	66
13	Naftidrofuryl		−0.3	4	−0.653	0.03428	0.0065	50
14	Metixene		−0.273	4	−0.65	0.03555	0.0615	50
15	Zidovudine		0.281	4	0.644	0.03873	0.0245	50
16	Metergoline		0.336	4	0.638	0.04205	0.1726	50
17	Harmaline		−0.268	4	−0.636	0.04255	0.0353	50

## Data Availability

The data used to support the findings of this study are included within Supplementary Materials.

## Abbreviations

ICI: Immune cell infiltration
LGG: Low-grade glioma
PCA: Principal component analysis
TMB: Tumor mutation burden
CGGA: Chinese Glioma Genome Atlas
CDF: Cumulative distribution function
DEGs: Differentially expressed genes
FC: Fold change
PC1: Principal component 1
GO: Gene ontology
KEGG: Kyoto Encyclopedia of Genes and Genomes
GSEA: Gene set enrichment analysis
CMap: Connectivity Map
MoA: Mode-of-action
OS.: Overall survival.

## Supplementary Materials

Supplementary Materials Supplementary Table 1: the list of ICI gene signatures A and B. Supplementary Table 2: GSEA results in high ICI score group. Supplementary Table 3: GSEA results in low ICI score group. Supplementary Table 4: DEGs between high and low ICI score groups. Supplementary Figure 1: validation of the three ICI subtypes for LGG in the CGGA-LGG dataset. (A–C) Unsupervised clustering analysis for validating the classifications of three ICI subtypes. (A) Consensus cumulative distribution function graph. (B) Delta area plot. (C) Heatmap for consensus matrix when *k* = 3. (D) Heatmap of tumor-infiltrating immune cells in different clinical phenotypes and ICI subtypes. (E) PCA plots for confirming the classification patterns of the ICI subtypes. (F) Kaplan-Meier curves for OS of LGG patients in the three ICI subtypes.
