# Supplementary material for: Uncovering the Immune Cell Infiltration Landscape in Low-Grade Glioma for Aiding Immunotherapy
Source: J Oncol. 2022 Mar 11;2022:3370727. doi: 10.1155/2022/3370727 (PMC8933094; doi:10.1155/2022/3370727)
Supplement: Supplementary Materials — Supplementary Table 1: the list of ICI gene signatures A and B. Supplementary Table 2: GSEA results in high ICI score group. Supplementary Table 3: GSEA results in low ICI score group. Supplementary Table 4: DEGs between high and low ICI score groups. Supplementary Figure 1: validation of the three ICI subtypes for LGG in the CGGA-LGG dataset. (A–C) Unsupervised clustering analysis for validating the classifications of three ICI subtypes. (A) Consensus cumulative distribution function graph. (B) Delta area plot. (C) Heatmap for consensus matrix when k = 3. (D) Heatmap of tumor-infiltrating immune cells in different clinical phenotypes and ICI subtypes. (E) PCA plots for confirming the classification patterns of the ICI subtypes. (F) Kaplan-Meier curves for OS of LGG patients in the three ICI subtypes. [file 3370727.f1.zip › 3370727.f1/Supplementary table 1.pdf]

Supplementary table 1. The list of ICI gene signatures A and B.

ICI gene signature A	ICI gene signature B
CHRM3	IL1B
ENC1	CFI
C11orf87	NTSR2
RAB15	HLA-DQB1
KCNS1	EGR2
C1QL3	CRLF1
RGS4	ACY3
SLC30A3	OSM
HS6ST3	HLA-DQA1
HCN1	SPP1
MAP7D2	SPOCD1
DNM1	ETNPPL
ZCCHC12	GPNMB
TESPA1	HLA-DQA2
SYT7	CXCL2
RBFOX1	LYZ
SLC6A7	CHI3L2
STX1A	SERPINA1
MAL2	HLA-DPA1
EPHB6	NAPSB
GDA	TGFBI
CACNG3	ALOX5AP
GABRB2	HLA-DRB5
CAMK1G	SLA
PHF24	OLR1
NEURL1	HLA-DOA
TMEM155	LINC01736
RBP4	HLA-DRB1
PTPN5	CCL2
SLC6A17	HLA-DRB6
DOC2A	HLA-DPB1
NEFH	HLA-DRA
SLC4A10	PRLHR
CREG2	TYMP
NAPB	AGT
SV2B	RASL10A
SLC12A5	RGS1
GABRA1	SLC11A1
NEFM	HSPB8
PCP4L1	ANKRD22
PCSK1	C3
NGB	IGSF1
DLX1	TNFSF18
SYT1	CD163
MPPED1	SLC15A2
KCNC2	FCGR3A
AMPH	S100A11
NELL2	MSR1
GNG3	BBOX1
GAD2	CD74

NEFL	IER3
SCN2A	LPCAT2
VSNL1	NTRK2
CAMK2A	CH25H
TMEM130	FCGBP
HPCA	RNASE2
SLC32A1	ALOX5
SYNGR3	CCL3L1
SYT13	CCL4
SULT4A1	CCL4L2
DDN	AL031056.1
CRYM	DHRS9
NRGN	FPR1
EEF1A2	P2RY12
KCNH3	IFI44L
NGEF	LYVE1
PHYHIP	SELL
CLEC2L	LINC01338
RFPL1S	UNC93B1
GABRA5	ITGB4
SYN2	ST14
RAB3A	TMEM119
ATP8A2	PLD4
PACSIN1	CEBPA
ICAM5	DAAM2
CHD5	PHKG1
RBFOX3	SELPLG
TMEM132D	LIX1
CALY	TLR2
NPTX1	PLCB2
CCK	CCL3
RIMS1	SFRP2
LHX2	CD38
PRKCG	CX3CR1
SYNPR	S100A10
PPP1R14C	SUSD3
KCNJ4	FYB1
VSTM2L	RARRES3
SNAP25	SH3TC1
TCEAL6	STOX1
ATP2B3	GGTA1P
CHRM1	ITGAM
MYT1L	CD44
RPH3A	ITGAX
SLC17A7	SLC25A48
RGS7BP	APBB1IP
CELF4	TNF
FAM84A	LRRC25
NPM2	AC073486.1
PVALB	HLA-DMA
CAMKV	EVI2B
CPNE6	THEMIS2
RAB3C	ADCY8

IQSEC3	SAMSN1
SYN1	HCST
FBXL16	CSF3R
SYT4	CD53
SNCB	BLNK
SHANK1	USH1C
PTPRN	HLA-DMB
CBLN2	SLC2A5
FXYD7	FCGR2A
CDK5R2	SIGLEC10
WSCD2	GBP2
GABRG2	C3AR1
RIMS4	IL18
SHC3	PLEK
CABP1	HCLS1
SHISA9	C4B
FAM19A2	FPR3
GRIN1	SCIN
STMN2	RPE65
SLC8A2	SASH3
SVOP	LAT2
LRTM2	HAVCR2
SNCG	CSF2RA
RIMS2	CCR1
CPLX2	MILR1
VGF	BTK
GJB6	LAIR1
PCSK2	MNDA
FAM163B	DEF6
HPCAL4	MYO1F
CHGA	PCED1B-AS1
SLC35F1	LILRB1
TMEM151B	TMIGD3
L1CAM	SLC14A1
SPRY4	HPSE2
GABRD	CYTH4
UBE2T	ITGB2
CREB3L1	CSF1R
DUSP4	TLR7
ETV5	DOCK2
VSTM2A	CTSS
4-Mar	LPAR5
CELF3	VCAM1
CHI3L1	GFAP
EGR3	CLEC9A
SNAP91	MKX
ETV4	RNASET2
ACBD7	FCGR1B
IGFBP2	PI16
FOSB	VAV1
AL022313.4	FCGR1A
INA	ABI3
TIMP1	RHBDF2

PLAT	CD84
POSTN	GPR34
NEK2	LINC01094
JPH3	PIK3R5
TRH	PARVG
TRIM67	FAM189A2
PTTG1	C1QB
SPC25	WAS
NSG2	CD86
PPP1R1B	P2RY13
TYMS	LST1
CD248	MS4A6A
KIF23	NCKAP1L
COL6A2	CYBB
FANCI	LAPTM5
H19	LUZP2
TPX2	IRF5
FOXM1	TREM2
SPC24	RNASE6
CENPF	PTPN6
AC141557.1	SPI1
KIF4A	SYK
PCLAF	HPGDS
DTL	ADAM28
RRM2	FERMT3
HJURP	AIF1
FREM2	CD37
METTL7B	PTAFR
PBK	LINC01532
MELK	LGALS9
COL1A2	TBXAS1
PIMREG	FCER1G
DPEP1	C1QC
NCAPH	GFPT2
TK1	CYBA
TOP2A	HCK
CDK1	CD99
KIF20A	CD300A
BUB1	ACSM5
NGFR	RGS10
MYBL2	SIGLEC8
CDC45	LY86
NCAPG	CD14
GTSE1	C1QA
COL4A2	VAMP8
EGR1	FCGR1CP
DLGAP5	IL10RA
MKI67	AOAH
LTF	LILRB4
BUB1B	C1orf162
CENPU	GPX3
HSPG2	PYCARD
NUSAP1	TYROBP

KIFC1	ABI3BP
NDC80	IRF8
COL3A1	VSIG4
ASF1B	APOE
BIRC5	LINC01480
COL4A1	CTSH
TROAP	LINC00844
CCNB2	UCP2
COL1A1	TNFAIP8L2
MMP9	CHST9
TMSB15A	NFIA-AS2
GSX1	LYL1
ANXA1	MS4A4A
CDC20	EBI3
GFRA1	SYTL4
UBE2C	NCF4
AURKB	RGS9
TLX1	GYPC
S100A4	AL161785.1
MGP	MS4A7
RANBP3L	TPTEP1
FSTL5	LINC01088
	NMB
	MSTN
	CRYAB
	FOLR2
	LINC00836
