# Supplementary material for: Uncovering the Immune Cell Infiltration Landscape in Low-Grade Glioma for Aiding Immunotherapy
Source: J Oncol. 2022 Mar 11;2022:3370727. doi: 10.1155/2022/3370727 (PMC8933094; doi:10.1155/2022/3370727)
Supplement: Supplementary Materials — Supplementary Table 1: the list of ICI gene signatures A and B. Supplementary Table 2: GSEA results in high ICI score group. Supplementary Table 3: GSEA results in low ICI score group. Supplementary Table 4: DEGs between high and low ICI score groups. Supplementary Figure 1: validation of the three ICI subtypes for LGG in the CGGA-LGG dataset. (A–C) Unsupervised clustering analysis for validating the classifications of three ICI subtypes. (A) Consensus cumulative distribution function graph. (B) Delta area plot. (C) Heatmap for consensus matrix when k = 3. (D) Heatmap of tumor-infiltrating immune cells in different clinical phenotypes and ICI subtypes. (E) PCA plots for confirming the classification patterns of the ICI subtypes. (F) Kaplan-Meier curves for OS of LGG patients in the three ICI subtypes. [file 3370727.f1.zip › 3370727.f1/Supplementary table 2.pdf]

Supplementary table 2. GSEA results in high ICI score group.

NAME
KEGG_CARDIAC_MUSCLE_CONTRACTION
KEGG_NEUROACTIVE_LIGAND_RECEPTOR_INTERACTION
KEGG_CALCIUM_SIGNALING_PATHWAY
KEGG_LONG_TERM_POTENTIATION
KEGG_AMYOTROPHIC_LATERAL_SCLEROSIS_ALS
KEGG_OXIDATIVE_PHOSPHORYLATION
KEGG_LONG_TERM_DEPRESSION
KEGG_ALZHEIMERS_DISEASE
KEGG_GNRH_SIGNALING_PATHWAY
KEGG_PROXIMAL_TUBULE_BICARBONATE_RECLAMATION
KEGG_TYPE_II_DIABETES_MELLITUS
KEGG_PHOSPHATIDYLINOSITOL_SIGNALING_SYSTEM
KEGG_ALDOSTERONE_REGULATED_SODIUM_REABSORPTION
KEGG_ALANINE_ASPARTATE_AND_GLUTAMATE_METABOLISM
KEGG_VASCULAR_SMOOTH_MUSCLE_CONTRACTION
KEGG_PARKINSONS_DISEASE
KEGG_GAP_JUNCTION
KEGG_TASTE_TRANSDUCTION
KEGG_MELANOGENESIS
KEGG_TERPENOID_BACKBONE_BIOSYNTHESIS
KEGG_HUNTINGTONS_DISEASE
KEGG_DILATED_CARDIOMYOPATHY
KEGG_BUTANOATE_METABOLISM
KEGG_AXON_GUIDANCE
KEGG_ERBB_SIGNALING_PATHWAY
KEGG_OOCYTE_MEIOSIS
KEGG_WNT_SIGNALING_PATHWAY
KEGG_MAPK_SIGNALING_PATHWAY
KEGG_STEROID_BIOSYNTHESIS
KEGG_EPITHELIAL_CELL_SIGNALING_IN_HELICOBACTER_PYLORI_INFECTION
KEGG_PYRUVATE_METABOLISM
KEGG_NITROGEN_METABOLISM
KEGG_VIBRIO_CHOLERAE_INFECTION
KEGG_ENDOCYTOSIS
KEGG_HYPERTROPHIC_CARDIOMYOPATHY_HCM
KEGG_ARRHYTHMOGENIC_RIGHT_VENTRICULAR_CARDIOMYOPATHY_ARVC
KEGG_VASOPRESSIN_REGULATED_WATER_REABSORPTION
KEGG_CITRATE_CYCLE_TCA_CYCLE
KEGG_GLYCOSAMINOGLYCAN_BIOSYNTHESIS_HEPARAN_SULFATE
KEGG_GLIOMA
KEGG_ARGININE_AND_PROLINE_METABOLISM
KEGG_INOSITOL_PHOSPHATE_METABOLISM
KEGG_OLFACTORY_TRANSDUCTION
KEGG_O_GLYCAN_BIOSYNTHESIS
KEGG_GLYCEROPHOSPHOLIPID_METABOLISM
KEGG_HEDGEHOG_SIGNALING_PATHWAY
KEGG_BIOSYNTHESIS_OF_UNSATURATED_FATTY_ACIDS
KEGG_GLYCOSPHINGOLIPID_BIOSYNTHESIS_LACTO_AND_NEOLACTO_SERIES
KEGG_GLYCEROLIPID_METABOLISM
KEGG_GLYOXYLATE_AND_DICARBOXYLATE_METABOLISM
KEGG_PROGESTERONE_MEDIATED_OOCYTE_MATURATION

KEGG_PURINE_METABOLISM
KEGG_MTOR_SIGNALING_PATHWAY
KEGG_DORSO_VENTRAL_AXIS_FORMATION
KEGG_THYROID_CANCER
KEGG_RIBOFLAVIN_METABOLISM
KEGG_ENDOMETRIAL_CANCER
KEGG_REGULATION_OF_AUTOPHAGY
KEGG_LINOLEIC_ACID_METABOLISM
KEGG_BASAL_CELL_CARCINOMA
KEGG_STEROID_HORMONE_BIOSYNTHESIS

GS
 follow link to MSigDB
KEGG_CARDIAC_MUSCLE_CONTRACTION
KEGG_NEUROACTIVE_LIGAND_RECEPTOR_INTERACTION
KEGG_CALCIUM_SIGNALING_PATHWAY
KEGG_LONG_TERM_POTENTIATION
KEGG_AMYOTROPHIC_LATERAL_SCLEROSIS_ALS
KEGG_OXIDATIVE_PHOSPHORYLATION
KEGG_LONG_TERM_DEPRESSION
KEGG_ALZHEIMERS_DISEASE
KEGG_GNRH_SIGNALING_PATHWAY
KEGG_PROXIMAL_TUBULE_BICARBONATE_RECLAMATION
KEGG_TYPE_II_DIABETES_MELLITUS
KEGG_PHOSPHATIDYLINOSITOL_SIGNALING_SYSTEM
KEGG_ALDOSTERONE_REGULATED_SODIUM_REABSORPTION
KEGG_ALANINE_ASPARTATE_AND_GLUTAMATE_METABOLISM
KEGG_VASCULAR_SMOOTH_MUSCLE_CONTRACTION
KEGG_PARKINSONS_DISEASE
KEGG_GAP_JUNCTION
KEGG_TASTE_TRANSDUCTION
KEGG_MELANOGENESIS
KEGG_TERPENOID_BACKBONE_BIOSYNTHESIS
KEGG_HUNTINGTONS_DISEASE
KEGG_DILATED_CARDIOMYOPATHY
KEGG_BUTANOATE_METABOLISM
KEGG_AXON_GUIDANCE
KEGG_ERBB_SIGNALING_PATHWAY
KEGG_OOCYTE_MEIOSIS
KEGG_WNT_SIGNALING_PATHWAY
KEGG_MAPK_SIGNALING_PATHWAY
KEGG_STEROID_BIOSYNTHESIS
KEGG_EPITHELIAL_CELL_SIGNALING_IN_HELICOBACTER_PYLORI_INFECTION
KEGG_PYRUVATE_METABOLISM
KEGG_NITROGEN_METABOLISM
KEGG_VIBRIO_CHOLERAE_INFECTION
KEGG_ENDOCYTOSIS
KEGG_HYPERTROPHIC_CARDIOMYOPATHY_HCM
KEGG_ARRHYTHMOGENIC_RIGHT_VENTRICULAR_CARDIOMYOPATHY_ARVC
KEGG_VASOPRESSIN_REGULATED_WATER_REABSORPTION
KEGG_CITRATE_CYCLE_TCA_CYCLE
KEGG_GLYCOSAMINOGLYCAN_BIOSYNTHESIS_HEPARAN_SULFATE
KEGG_GLIOMA
KEGG_ARGININE_AND_PROLINE_METABOLISM
KEGG_INOSITOL_PHOSPHATE_METABOLISM
KEGG_OLFACTORY_TRANSDUCTION
KEGG_O_GLYCAN_BIOSYNTHESIS
KEGG_GLYCEROPHOSPHOLIPID_METABOLISM
KEGG_HEDGEHOG_SIGNALING_PATHWAY
KEGG_BIOSYNTHESIS_OF_UNSATURATED_FATTY_ACIDS
KEGG_GLYCOSPHINGOLIPID_BIOSYNTHESIS_LACTO_AND_NEOLACTO_SERIES
KEGG_GLYCEROLIPID_METABOLISM
KEGG_GLYOXYLATE_AND_DICARBOXYLATE_METABOLISM
KEGG_PROGESTERONE_MEDIATED_OOCYTE_MATURATION

KEGG_PURINE_METABOLISM
KEGG_MTOR_SIGNALING_PATHWAY
KEGG_DORSO_VENTRAL_AXIS_FORMATION
KEGG_THYROID_CANCER
KEGG_RIBOFLAVIN_METABOLISM
KEGG_ENDOMETRIAL_CANCER
KEGG_REGULATION_OF_AUTOPHAGY
KEGG_LINOLEIC_ACID_METABOLISM
KEGG_BASAL_CELL_CARCINOMA
KEGG_STEROID_HORMONE_BIOSYNTHESIS

GS DETAILS	SIZE	ES	NES	NOM p-val
Details ...	79	0.6893506	2.2257407	0
Details ...	271	0.590269	2.2022285	0
Details ...	177	0.6079409	2.0931687	0.001976285
Details ...	70	0.6042397	1.8167198	0.01004016
Details ...	53	0.51041013	1.6992246	0.016129032
Details ...	132	0.55261284	1.6853911	0.046709128
Details ...	70	0.4940858	1.6778862	0.014522822
Details ...	166	0.4820421	1.671227	0.052742615
Details ...	101	0.484929	1.6629488	0.01778656
Details ...	23	0.5806086	1.6414751	0.022222223
Details ...	47	0.50719285	1.632974	0.022494888
Details ...	76	0.5144006	1.6302472	0.0331384
Details ...	42	0.51345474	1.6259866	0.02020202
Details ...	30	0.5059419	1.6221536	0.020408163
Details ...	114	0.44740775	1.6026537	0.028282829
Details ...	128	0.49659115	1.5786822	0.08125
Details ...	90	0.4599845	1.561013	0.03269231
Details ...	51	0.4522377	1.5596584	0.030800821
Details ...	101	0.44852298	1.5367002	0.03448276
Details ...	15	0.6445787	1.5163581	0.078313254
Details ...	181	0.42953897	1.4941825	0.10330579
Details ...	90	0.4239277	1.471857	0.07370518
Details ...	34	0.46888426	1.4621836	0.07692308
Details ...	129	0.4232511	1.4453819	0.1023166
Details ...	87	0.43754417	1.4324468	0.1015625
Details ...	113	0.4500263	1.4323508	0.105675146
Details ...	151	0.40914685	1.4282655	0.091836736
Details ...	267	0.38005167	1.4233106	0.0945674
Details ...	17	0.5964815	1.4136517	0.13184585
Details ...	68	0.42762086	1.4050277	0.114173226
Details ...	40	0.43705404	1.3846338	0.11359026
Details ...	23	0.45975348	1.3550926	0.1085595
Details ...	54	0.41956356	1.3525504	0.13496932
Details ...	181	0.38399848	1.3502678	0.13972056
Details ...	83	0.39345703	1.3491346	0.13414635
Details ...	74	0.40964356	1.335874	0.14931238
Details ...	44	0.43696493	1.3271601	0.1468254
Details ...	31	0.48933437	1.3206644	0.20384616
Details ...	26	0.44815063	1.2853693	0.19111969
Details ...	65	0.38934627	1.2787992	0.15779093
Details ...	54	0.3534421	1.2420741	0.20934959
Details ...	54	0.39515093	1.2384051	0.20874752
Details ...	386	0.28558302	1.2284213	0.14541833
Details ...	30	0.42459512	1.2141396	0.247505
Details ...	77	0.33096194	1.1891606	0.23829788
Details ...	56	0.36065084	1.163238	0.27155173
Details ...	22	0.40696907	1.1291399	0.33988214
Details ...	26	0.3641827	1.1166883	0.31349206
Details ...	49	0.30872554	1.0885465	0.31702128
Details ...	16	0.3762084	1.0770141	0.36895162
Details ...	85	0.33922425	1.0766641	0.38779527

Details ...	157	0.29234964	1.0552031	0.38339922
Details ...	52	0.32580918	1.0516918	0.41308793
Details ...	24	0.36405018	1.045759	0.38336715
Details ...	29	0.34684855	1.0192394	0.4352227
Details ...	15	0.36846802	1.0007995	0.46105263
Details ...	52	0.3243684	0.99499494	0.4837476
Details ...	35	0.30759722	0.9496357	0.52362204
Details ...	29	0.2895373	0.9238739	0.5740319
Details ...	55	0.28667954	0.82068944	0.69911504
	55	0.20656188	0.66275936	0.93453723

FDR q-val	FWER p-val	RANK AT MAX	LEADING EDGE
0	0	6755	tags=49%, list=12%, signal=56%
0	0	6410	tags=42%, list=12%, signal=47%
0.001168491	0.004	4301	tags=40%, list=8%, signal=43%
0.053350978	0.153	5974	tags=53%, list=11%, signal=59%
0.14304605	0.348	7253	tags=51%, list=13%, signal=59%
0.13586551	0.377	8318	tags=48%, list=15%, signal=56%
0.12463728	0.391	8258	tags=53%, list=15%, signal=62%
0.11426516	0.402	6954	tags=40%, list=13%, signal=45%
0.10802315	0.421	4160	tags=32%, list=8%, signal=34%
0.11534264	0.459	10822	tags=57%, list=20%, signal=70%
0.11327883	0.481	4511	tags=40%, list=8%, signal=44%
0.10606085	0.484	4949	tags=41%, list=9%, signal=45%
0.10126253	0.494	8421	tags=45%, list=15%, signal=53%
0.0963475	0.498	6467	tags=40%, list=12%, signal=45%
0.10395023	0.532	4160	tags=29%, list=8%, signal=31%
0.11723283	0.572	8318	tags=45%, list=15%, signal=53%
0.12474677	0.603	4160	tags=31%, list=8%, signal=34%
0.11869627	0.603	4110	tags=20%, list=7%, signal=21%
0.1316227	0.637	4160	tags=28%, list=8%, signal=30%
0.14263335	0.668	3793	tags=47%, list=7%, signal=50%
0.15549242	0.703	7877	tags=40%, list=14%, signal=47%
0.16912028	0.733	3236	tags=24%, list=6%, signal=26%
0.17096546	0.748	4644	tags=35%, list=8%, signal=39%
0.1801693	0.766	3812	tags=28%, list=7%, signal=30%
0.18423219	0.783	6906	tags=37%, list=12%, signal=42%
0.17733474	0.784	4972	tags=29%, list=9%, signal=32%
0.17423555	0.789	5191	tags=26%, list=9%, signal=28%
0.17300206	0.8	4154	tags=27%, list=8%, signal=29%
0.17515096	0.814	6256	tags=65%, list=11%, signal=73%
0.17713916	0.82	2741	tags=28%, list=5%, signal=29%
0.19014405	0.845	8110	tags=43%, list=15%, signal=50%
0.21061714	0.872	531	tags=13%, list=1%, signal=13%
0.20662305	0.873	2644	tags=26%, list=5%, signal=27%
0.20270622	0.873	5854	tags=30%, list=11%, signal=33%
0.19807391	0.873	2290	tags=20%, list=4%, signal=21%
0.20476282	0.881	2779	tags=23%, list=5%, signal=24%
0.20713478	0.885	8942	tags=43%, list=16%, signal=51%
0.20732298	0.891	8059	tags=42%, list=15%, signal=49%
0.23447993	0.914	4831	tags=31%, list=9%, signal=34%
0.23526329	0.916	4511	tags=26%, list=8%, signal=28%
0.26506874	0.937	3283	tags=15%, list=6%, signal=16%
0.26264155	0.941	4949	tags=30%, list=9%, signal=33%
0.26706085	0.944	11571	tags=18%, list=21%, signal=23%
0.27589837	0.947	4870	tags=33%, list=9%, signal=37%
0.2952496	0.959	5702	tags=25%, list=10%, signal=27%
0.3148413	0.961	5994	tags=23%, list=11%, signal=26%
0.34461844	0.971	7210	tags=27%, list=13%, signal=31%
0.35179135	0.975	4493	tags=27%, list=8%, signal=29%
0.37770405	0.982	3581	tags=18%, list=6%, signal=20%
0.38339084	0.985	2978	tags=19%, list=5%, signal=20%
0.3762718	0.985	5261	tags=29%, list=10%, signal=32%

0.39380774	0.988	4268	tags=15%, list=8%, signal=16%
0.39026973	0.988	9212	tags=40%, list=17%, signal=48%
0.38944167	0.989	8197	tags=38%, list=15%, signal=44%
0.41215566	0.992	6371	tags=24%, list=12%, signal=27%
0.42595097	0.994	2691	tags=20%, list=5%, signal=21%
0.42531663	0.995	4511	tags=25%, list=8%, signal=27%
0.47271737	0.995	9292	tags=31%, list=17%, signal=38%
0.4965611	0.996	6289	tags=24%, list=11%, signal=27%
0.6268683	1	3784	tags=15%, list=7%, signal=16%
0.8296551	1	9173	tags=22%, list=17%, signal=26%
