# Supplementary material for: Uncovering the Immune Cell Infiltration Landscape in Low-Grade Glioma for Aiding Immunotherapy
Source: J Oncol. 2022 Mar 11;2022:3370727. doi: 10.1155/2022/3370727 (PMC8933094; doi:10.1155/2022/3370727)
Supplement: Supplementary Materials — Supplementary Table 1: the list of ICI gene signatures A and B. Supplementary Table 2: GSEA results in high ICI score group. Supplementary Table 3: GSEA results in low ICI score group. Supplementary Table 4: DEGs between high and low ICI score groups. Supplementary Figure 1: validation of the three ICI subtypes for LGG in the CGGA-LGG dataset. (A–C) Unsupervised clustering analysis for validating the classifications of three ICI subtypes. (A) Consensus cumulative distribution function graph. (B) Delta area plot. (C) Heatmap for consensus matrix when k = 3. (D) Heatmap of tumor-infiltrating immune cells in different clinical phenotypes and ICI subtypes. (E) PCA plots for confirming the classification patterns of the ICI subtypes. (F) Kaplan-Meier curves for OS of LGG patients in the three ICI subtypes. [file 3370727.f1.zip › 3370727.f1/Supplementary table 3.pdf]

Supplementary table 3. GSEA results in low ICI score group.

NAME
KEGG_PRIMARY_IMMUNODEFICIENCY
KEGG_ANTIGEN_PROCESSING_AND_PRESENTATION
KEGG_VIRAL_MYOCARDITIS
KEGG_LEUKOCYTE_TRANSENDOTHELIAL_MIGRATION
KEGG_LEISHMANIA_INFECTION
KEGG_JAK_STAT_SIGNALING_PATHWAY
KEGG_HEMATOPOIETIC_CELL_LINEAGE
KEGG_SYSTEMIC_LUPUS_ERYTHEMATOSUS
KEGG_CYTOSOLIC_DNA_SENSING_PATHWAY
KEGG_CYTOKINE_CYTOKINE_RECEPTOR_INTERACTION
KEGG_ASTHMA
KEGG_GLUTATHIONE_METABOLISM
KEGG_AUTOIMMUNE_THYROID_DISEASE
KEGG_NOTCH_SIGNALING_PATHWAY
KEGG_INTESTINAL_IMMUNE_NETWORK_FOR_IGA_PRODUCTION
KEGG_ALLOGRAFT_REJECTION
KEGG_TOLL_LIKE_RECEPTOR_SIGNALING_PATHWAY
KEGG_B_CELL_RECEPTOR_SIGNALING_PATHWAY
KEGG_CELL_ADHESION_MOLECULES_CAMS
KEGG_OTHER_GLYCAN_DEGRADATION
KEGG_HOMOLOGOUS_RECOMBINATION
KEGG_GRAFT_VERSUS_HOST_DISEASE
KEGG_PROTEASOME
KEGG_NUCLEOTIDE_EXCISION_REPAIR
KEGG_APOPTOSIS
KEGG_COMPLEMENT_AND_COAGULATION_CASCADES
KEGG_PATHOGENIC_ESCHERICHIA_COLI_INFECTION
KEGG_SMALL_CELL_LUNG_CANCER
KEGG_NATURAL_KILLER_CELL_MEDIATED_CYTOTOXICITY
KEGG_LYSOSOME
KEGG_PANCREATIC_CANCER
KEGG_REGULATION_OF_ACTIN_CYTOSKELETON
KEGG_DNA_REPLICATION
KEGG_PROTEIN_EXPORT
KEGG_CHRONIC_MYELOID_LEUKEMIA
KEGG_AMINO_SUGAR_AND_NUCLEOTIDE_SUGAR_METABOLISM
KEGG_PEROXISOME
KEGG_NOD_LIKE_RECEPTOR_SIGNALING_PATHWAY
KEGG_CHEMOKINE_SIGNALING_PATHWAY
KEGG_MISMATCH_REPAIR
KEGG_FC_GAMMA_R_MEDIATED_PHAGOCYTOSIS
KEGG_PATHWAYS_IN_CANCER
KEGG_NICOTINATE_AND_NICOTINAMIDE_METABOLISM
KEGG_PRIMARY_BILE_ACID_BIOSYNTHESIS
KEGG_PYRIMIDINE_METABOLISM
KEGG_BASE_EXCISION_REPAIR
KEGG_ONE_CARBON_POOL_BY_FOLATE
KEGG_ACUTE_MYELOID_LEUKEMIA
KEGG_ARACHIDONIC_ACID_METABOLISM
KEGG_FOCAL_ADHESION
KEGG_N_GLYCAN_BIOSYNTHESIS

KEGG_PANTOTHENATE_AND_COA_BIOSYNTHESIS
KEGG_SPLICEOSOME
KEGG_RIG_I_LIKE_RECEPTOR_SIGNALING_PATHWAY
KEGG_P53_SIGNALING_PATHWAY
KEGG_TYPE_I_DIABETES_MELLITUS
KEGG_VALINE_LEUCINE_AND_ISOLEUCINE_DEGRADATION
KEGG_GLYCOSAMINOGLYCAN_DEGRADATION
KEGG_TRYPTOPHAN_METABOLISM
KEGG_TGF_BETA_SIGNALING_PATHWAY
KEGG_CYSTEINE_AND_METHIONINE_METABOLISM
KEGG_ETHER_LIPID_METABOLISM
KEGG_T_CELL_RECEPTOR_SIGNALING_PATHWAY
KEGG_ADHERENS_JUNCTION
KEGG_GALACTOSE_METABOLISM
KEGG_GLYCINE_SERINE_AND_THREONINE_METABOLISM
KEGG_PROSTATE_CANCER
KEGG_ECM_RECEPTOR_INTERACTION
KEGG_FATTY_ACID_METABOLISM
KEGG_ABC_TRANSPORTERS
KEGG_CELL_CYCLE
KEGG_HISTIDINE_METABOLISM
KEGG_FC_EPSILON_RI_SIGNALING_PATHWAY
KEGG_SPHINGOLIPID_METABOLISM
KEGG_BLADDER_CANCER
KEGG_LYSINE_DEGRADATION
KEGG_PENTOSE_PHOSPHATE_PATHWAY
KEGG_TIGHT_JUNCTION
KEGG_BETA_ALANINE_METABOLISM
KEGG_COLORECTAL_CANCER
KEGG_STARCH_AND_SUCROSE_METABOLISM
KEGG_MELANOMA
KEGG_VEGF_SIGNALING_PATHWAY
KEGG_ADIPOCYTOKINE_SIGNALING_PATHWAY
KEGG_BASAL_TRANSCRIPTION_FACTORS
KEGG_RENAL_CELL_CARCINOMA
KEGG_PPAR_SIGNALING_PATHWAY
KEGG_GLYCOSYLPHOSPHATIDYLINOSITOL_GPI_ANCHOR_BIOSYNTHESIS
KEGG_RNA_DEGRADATION
KEGG_RIBOSOME
KEGG_RENIN_ANGIOTENSIN_SYSTEM
KEGG_GLYCOSAMINOGLYCAN_BIOSYNTHESIS_CHONDROITIN_SULFATE
KEGG_ASCORBATE_AND_ALDARATE_METABOLISM
KEGG_MATURITY_ONSET_DIABETES_OF_THE_YOUNG
KEGG_INSULIN_SIGNALING_PATHWAY
KEGG_SNARE_INTERACTIONS_IN_VESICULAR_TRANSPORT
KEGG_RNA_POLYMERASE
KEGG_FRUCTOSE_AND_MANNOSE_METABOLISM
KEGG_NEUROTROPHIN_SIGNALING_PATHWAY
KEGG_NON_SMALL_CELL_LUNG_CANCER
KEGG_PRION_DISEASES
KEGG_DRUG_METABOLISM_CYTOCHROME_P450
KEGG_ALPHA_LINOLENIC_ACID_METABOLISM
KEGG_SELENOAMINO_ACID_METABOLISM

KEGG_DRUG_METABOLISM_OTHER_ENZYMES
KEGG_GLYCOLYSIS_GLUCONEOGENESIS
KEGG_PORPHYRIN_AND_CHLOROPHYLL_METABOLISM
KEGG_GLYCOSAMINOGLYCAN_BIOSYNTHESIS_KERATAN_SULFATE
KEGG_METABOLISM_OF_XENOBIOTICS_BY_CYTOCHROME_P450
KEGG_PROPANOATE_METABOLISM
KEGG_GLYCOSPHINGOLIPID_BIOSYNTHESIS_GANGLIO_SERIES
KEGG_TYROSINE_METABOLISM
KEGG_PHENYLALANINE_METABOLISM
KEGG_RETINOL_METABOLISM
KEGG_UBIQUITIN_MEDIATED_PROTEOLYSIS
KEGG_AMINOACYL_TRNA_BIOSYNTHESIS
KEGG_PENTOSE_AND_GLUCURONATE_INTERCONVERSIONS

GS
 follow link to MSigDB
KEGG_PRIMARY_IMMUNODEFICIENCY
KEGG_ANTIGEN_PROCESSING_AND_PRESENTATION
KEGG_VIRAL_MYOCARDITIS
KEGG_LEUKOCYTE_TRANSENDOTHELIAL_MIGRATION
KEGG_LEISHMANIA_INFECTION
KEGG_JAK_STAT_SIGNALING_PATHWAY
KEGG_HEMATOPOIETIC_CELL_LINEAGE
KEGG_SYSTEMIC_LUPUS_ERYTHEMATOSUS
KEGG_CYTOSOLIC_DNA_SENSING_PATHWAY
KEGG_CYTOKINE_CYTOKINE_RECEPTOR_INTERACTION
KEGG_ASTHMA
KEGG_GLUTATHIONE_METABOLISM
KEGG_AUTOIMMUNE_THYROID_DISEASE
KEGG_NOTCH_SIGNALING_PATHWAY
KEGG_INTESTINAL_IMMUNE_NETWORK_FOR_IGA_PRODUCTION
KEGG_ALLOGRAFT_REJECTION
KEGG_TOLL_LIKE_RECEPTOR_SIGNALING_PATHWAY
KEGG_B_CELL_RECEPTOR_SIGNALING_PATHWAY
KEGG_CELL_ADHESION_MOLECULES_CAMS
KEGG_OTHER_GLYCAN_DEGRADATION
KEGG_HOMOLOGOUS_RECOMBINATION
KEGG_GRAFT_VERSUS_HOST_DISEASE
KEGG_PROTEASOME
KEGG_NUCLEOTIDE_EXCISION_REPAIR
KEGG_APOPTOSIS
KEGG_COMPLEMENT_AND_COAGULATION_CASCADES
KEGG_PATHOGENIC_ESCHERICHIA_COLI_INFECTION
KEGG_SMALL_CELL_LUNG_CANCER
KEGG_NATURAL_KILLER_CELL_MEDIATED_CYTOTOXICITY
KEGG_LYSOSOME
KEGG_PANCREATIC_CANCER
KEGG_REGULATION_OF_ACTIN_CYTOSKELETON
KEGG_DNA_REPLICATION
KEGG_PROTEIN_EXPORT
KEGG_CHRONIC_MYELOID_LEUKEMIA
KEGG_AMINO_SUGAR_AND_NUCLEOTIDE_SUGAR_METABOLISM
KEGG_PEROXISOME
KEGG_NOD_LIKE_RECEPTOR_SIGNALING_PATHWAY
KEGG_CHEMOKINE_SIGNALING_PATHWAY
KEGG_MISMATCH_REPAIR
KEGG_FC_GAMMA_R_MEDIATED_PHAGOCYTOSIS
KEGG_PATHWAYS_IN_CANCER
KEGG_NICOTINATE_AND_NICOTINAMIDE_METABOLISM
KEGG_PRIMARY_BILE_ACID_BIOSYNTHESIS
KEGG_PYRIMIDINE_METABOLISM
KEGG_BASE_EXCISION_REPAIR
KEGG_ONE_CARBON_POOL_BY_FOLATE
KEGG_ACUTE_MYELOID_LEUKEMIA
KEGG_ARACHIDONIC_ACID_METABOLISM
KEGG_FOCAL_ADHESION
KEGG_N_GLYCAN_BIOSYNTHESIS

KEGG_PANTOTHENATE_AND_COA_BIOSYNTHESIS
KEGG_SPLICEOSOME
KEGG_RIG_I_LIKE_RECEPTOR_SIGNALING_PATHWAY
KEGG_P53_SIGNALING_PATHWAY
KEGG_TYPE_I_DIABETES_MELLITUS
KEGG_VALINE_LEUCINE_AND_ISOLEUCINE_DEGRADATION
KEGG_GLYCOSAMINOGLYCAN_DEGRADATION
KEGG_TRYPTOPHAN_METABOLISM
KEGG_TGF_BETA_SIGNALING_PATHWAY
KEGG_CYSTEINE_AND_METHIONINE_METABOLISM
KEGG_ETHER_LIPID_METABOLISM
KEGG_T_CELL_RECEPTOR_SIGNALING_PATHWAY
KEGG_ADHERENS_JUNCTION
KEGG_GALACTOSE_METABOLISM
KEGG_GLYCINE_SERINE_AND_THREONINE_METABOLISM
KEGG_PROSTATE_CANCER
KEGG_ECM_RECEPTOR_INTERACTION
KEGG_FATTY_ACID_METABOLISM
KEGG_ABC_TRANSPORTERS
KEGG_CELL_CYCLE
KEGG_HISTIDINE_METABOLISM
KEGG_FC_EPSILON_RI_SIGNALING_PATHWAY
KEGG_SPHINGOLIPID_METABOLISM
KEGG_BLADDER_CANCER
KEGG_LYSINE_DEGRADATION
KEGG_PENTOSE_PHOSPHATE_PATHWAY
KEGG_TIGHT_JUNCTION
KEGG_BETA_ALANINE_METABOLISM
KEGG_COLORECTAL_CANCER
KEGG_STARCH_AND_SUCROSE_METABOLISM
KEGG_MELANOMA
KEGG_VEGF_SIGNALING_PATHWAY
KEGG_ADIPOCYTOKINE_SIGNALING_PATHWAY
KEGG_BASAL_TRANSCRIPTION_FACTORS
KEGG_RENAL_CELL_CARCINOMA
KEGG_PPAR_SIGNALING_PATHWAY
KEGG_GLYCOSYLPHOSPHATIDYLINOSITOL_GPI_ANCHOR_BIOSYNTHESIS
KEGG_RNA_DEGRADATION
KEGG_RIBOSOME
KEGG_RENIN_ANGIOTENSIN_SYSTEM
KEGG_GLYCOSAMINOGLYCAN_BIOSYNTHESIS_CHONDROITIN_SULFATE
KEGG_ASCORBATE_AND_ALDARATE_METABOLISM
KEGG_MATURITY_ONSET_DIABETES_OF_THE_YOUNG
KEGG_INSULIN_SIGNALING_PATHWAY
KEGG_SNARE_INTERACTIONS_IN_VESICULAR_TRANSPORT
KEGG_RNA_POLYMERASE
KEGG_FRUCTOSE_AND_MANNOSE_METABOLISM
KEGG_NEUROTROPHIN_SIGNALING_PATHWAY
KEGG_NON_SMALL_CELL_LUNG_CANCER
KEGG_PRION_DISEASES
KEGG_DRUG_METABOLISM_CYTOCHROME_P450
KEGG_ALPHA_LINOLENIC_ACID_METABOLISM
KEGG_SELENOAMINO_ACID_METABOLISM

KEGG_DRUG_METABOLISM_OTHER_ENZYMES
KEGG_GLYCOLYSIS_GLUCONEOGENESIS
KEGG_PORPHYRIN_AND_CHLOROPHYLL_METABOLISM
KEGG_GLYCOSAMINOGLYCAN_BIOSYNTHESIS_KERATAN_SULFATE
KEGG_METABOLISM_OF_XENOBIOTICS_BY_CYTOCHROME_P450
KEGG_PROPANOATE_METABOLISM
KEGG_GLYCOSPHINGOLIPID_BIOSYNTHESIS_GANGLIO_SERIES
KEGG_TYROSINE_METABOLISM
KEGG_PHENYLALANINE_METABOLISM
KEGG_RETINOL_METABOLISM
KEGG_UBIQUITIN_MEDIATED_PROTEOLYSIS
KEGG_AMINOACYL_TRNA_BIOSYNTHESIS
KEGG_PENTOSE_AND_GLUCURONATE_INTERCONVERSIONS

GS DETAILS	SIZE	ES	NES	NOM p-val
Details ...	35	-0.8286968	-2.057215	0
Details ...	81	-0.6969206	-1.998534	0.001926782
Details ...	68	-0.6875897	-1.9896119	0.003875969
Details ...	116	-0.6099798	-1.9667872	0.002024292
Details ...	70	-0.74108535	-1.952677	0.004016064
Details ...	155	-0.56840396	-1.9432997	0.001934236
Details ...	85	-0.6862897	-1.9415112	0.001890359
Details ...	55	-0.76862025	-1.9196126	0.001937985
Details ...	54	-0.6047924	-1.9131732	0
Details ...	264	-0.60044336	-1.9099698	0.003773585
Details ...	28	-0.80536616	-1.900754	0
Details ...	49	-0.5834016	-1.8663957	0
Details ...	50	-0.7152182	-1.8606865	0.008080808
Details ...	47	-0.6302957	-1.8578384	0
Details ...	46	-0.72527194	-1.8237466	0.01532567
Details ...	35	-0.834586	-1.8178136	0.003929273
Details ...	102	-0.5705991	-1.8066123	0.006329114
Details ...	75	-0.59068954	-1.7990792	0.014403292
Details ...	131	-0.569308	-1.7713052	0.009746589
Details ...	16	-0.7442124	-1.769923	0.012195122
Details ...	28	-0.71386707	-1.7166854	0.020491803
Details ...	37	-0.77903694	-1.7160925	0.021484375
Details ...	46	-0.6773197	-1.7121307	0.025896415
Details ...	44	-0.6519145	-1.700467	0.022312373
Details ...	87	-0.5410313	-1.6995437	0.022821577
Details ...	69	-0.63834643	-1.6935406	0.011049724
Details ...	56	-0.56814355	-1.685746	0.028077753
Details ...	84	-0.551431	-1.6804429	0.022540983
Details ...	132	-0.51258993	-1.6761689	0.016494846
Details ...	121	-0.55256504	-1.6697285	0.042682927
Details ...	70	-0.5251154	-1.6648091	0.022267206
Details ...	213	-0.4780673	-1.6478511	0.020491803
Details ...	36	-0.7207846	-1.6452109	0.05811623
Details ...	24	-0.65546924	-1.6386507	0.022312373
Details ...	73	-0.5299066	-1.6340061	0.030241935
Details ...	43	-0.5611268	-1.6328418	0.03846154
Details ...	78	-0.51339686	-1.625469	0.03448276
Details ...	62	-0.54554355	-1.6224849	0.038610037
Details ...	188	-0.4796085	-1.6212304	0.027777778
Details ...	23	-0.71736336	-1.6087229	0.042105265
Details ...	96	-0.51072294	-1.5957589	0.044491526
Details ...	325	-0.45617974	-1.5918533	0.030364372
Details ...	24	-0.55123985	-1.5916319	0.029296875
Details ...	16	-0.60356134	-1.5481468	0.062015504
Details ...	98	-0.50529087	-1.5443504	0.06666667
Details ...	35	-0.59022653	-1.5345749	0.07645875
Details ...	17	-0.59104323	-1.5327433	0.05050505
Details ...	57	-0.4895457	-1.5156722	0.075664625
Details ...	58	-0.43639815	-1.5142436	0.031307552
Details ...	199	-0.4629844	-1.5122786	0.078947365
Details ...	46	-0.5766565	-1.5070969	0.07172996

Details ...	16	-0.568118	-1.4996834	0.062
Details ...	127	-0.55285025	-1.4963508	0.11394892
Details ...	70	-0.44095227	-1.4882116	0.06300813
Details ...	68	-0.48123875	-1.4788551	0.11787819
Details ...	41	-0.5971963	-1.4758015	0.11001965
Details ...	43	-0.50084287	-1.474065	0.08196721
Details ...	21	-0.5566308	-1.4627795	0.118644066
Details ...	39	-0.46542156	-1.4609054	0.078095235
Details ...	86	-0.43372068	-1.435515	0.07269155
	34	-0.45242992	-1.4291273	0.07539683
	33	-0.45311403	-1.42846	0.0662768
	108	-0.45083222	-1.4169371	0.118393235
	73	-0.44796017	-1.390114	0.11885246
	26	-0.4741396	-1.375776	0.12727273
	31	-0.4372392	-1.3705508	0.12854442
	89	-0.42400223	-1.3624784	0.13438736
	84	-0.49341187	-1.3570378	0.18326694
	42	-0.4251729	-1.3556315	0.11742424
	44	-0.4249104	-1.3529084	0.129771
	125	-0.48140642	-1.3505169	0.20570265
	29	-0.4326739	-1.3271074	0.13409962
	79	-0.38429767	-1.3107426	0.16568047
	39	-0.42102942	-1.295073	0.18052739
	42	-0.41415775	-1.2844292	0.17391305
	44	-0.43477485	-1.2661736	0.25254583
	27	-0.4075981	-1.2541105	0.20440882
	132	-0.3442739	-1.2538707	0.17171717
	22	-0.4046734	-1.2533773	0.1764706
	62	-0.40972662	-1.251894	0.22745901
	52	-0.41219	-1.2518576	0.18250951
	71	-0.3734855	-1.2481638	0.1780303
	76	-0.35154012	-1.2479154	0.21135029
	67	-0.36148116	-1.2309109	0.22580644
	35	-0.43958962	-1.2219017	0.2534113
	70	-0.38283908	-1.2174798	0.23966943
	69	-0.352783	-1.2025563	0.19675091
	25	-0.457921	-1.1733388	0.28367347
	59	-0.43347844	-1.1715169	0.33333334
	88	-0.59389055	-1.1704766	0.369863
	17	-0.428112	-1.1606092	0.2591171
	22	-0.41818395	-1.146751	0.31875
	25	-0.44721922	-1.1464466	0.3024055
	25	-0.39342764	-1.1431137	0.26820603
	137	-0.32362556	-1.1304117	0.3122449
	38	-0.36933684	-1.1274949	0.33604887
	29	-0.41845706	-1.1196597	0.36309522
	33	-0.35045752	-1.1148016	0.29962546
	126	-0.3339198	-1.1097574	0.3677686
	54	-0.34889057	-1.1077036	0.33732533
	35	-0.35351527	-1.0958328	0.35203093
	71	-0.34640193	-1.0951229	0.33676976
	19	-0.36413464	-1.0841019	0.34369287
	25	-0.36959553	-1.0655848	0.4110672

51	-0.3223599	-1.0362709	0.41145834
62	-0.31726155	-1.0301338	0.41132075
40	-0.3432143	-1.0236673	0.43130434
15	-0.40279993	-1.018133	0.43373495
69	-0.31416106	-0.9797646	0.48205128
32	-0.32457265	-0.92483896	0.530572
15	-0.3549754	-0.9241986	0.53515625
42	-0.28741482	-0.9234233	0.5740072
18	-0.3427511	-0.9199373	0.573055
64	-0.2734908	-0.8541133	0.6810345
135	-0.26713467	-0.8006661	0.6802444
22	-0.30977273	-0.7504424	0.7197581
28	-0.26486066	-0.7198754	0.85223365

FDR q-val	FWER p-val	RANK AT MAX	LEADING EDGE
0.015938725	0.005	4410	tags=69%, list=8%, signal=74%
0.019161468	0.022	4417	tags=47%, list=8%, signal=51%
0.014218054	0.024	4417	tags=49%, list=8%, signal=53%
0.014578912	0.029	5364	tags=45%, list=10%, signal=50%
0.013206145	0.035	6020	tags=71%, list=11%, signal=80%
0.011933124	0.038	6998	tags=39%, list=13%, signal=44%
0.010639616	0.039	8965	tags=58%, list=16%, signal=69%
0.013759207	0.055	4952	tags=65%, list=9%, signal=72%
0.013401932	0.061	6932	tags=41%, list=13%, signal=47%
0.012233649	0.061	10075	tags=51%, list=18%, signal=62%
0.012938186	0.07	7654	tags=71%, list=14%, signal=83%
0.018499259	0.104	12689	tags=59%, list=23%, signal=77%
0.01807219	0.111	4417	tags=48%, list=8%, signal=52%
0.017574418	0.115	7354	tags=40%, list=13%, signal=47%
0.024523044	0.156	7654	tags=65%, list=14%, signal=76%
0.024538131	0.162	7654	tags=86%, list=14%, signal=99%
0.025419608	0.175	5932	tags=48%, list=11%, signal=54%
0.025867626	0.184	4370	tags=45%, list=8%, signal=49%
0.03435652	0.221	5371	tags=43%, list=10%, signal=47%
0.03359965	0.222	2776	tags=50%, list=5%, signal=53%
0.054697875	0.309	8737	tags=68%, list=16%, signal=81%
0.052485097	0.312	7654	tags=73%, list=14%, signal=85%
0.05206422	0.323	8928	tags=52%, list=16%, signal=62%
0.05488579	0.349	9864	tags=59%, list=18%, signal=72%
0.053124905	0.35	8720	tags=57%, list=16%, signal=68%
0.053610794	0.354	6842	tags=42%, list=12%, signal=48%
0.055954155	0.375	6240	tags=45%, list=11%, signal=50%
0.057336442	0.389	10070	tags=51%, list=18%, signal=63%
0.05710695	0.394	4508	tags=35%, list=8%, signal=38%
0.05788798	0.399	5867	tags=46%, list=11%, signal=52%
0.05836963	0.404	10167	tags=61%, list=18%, signal=75%
0.06496051	0.447	8092	tags=45%, list=15%, signal=53%
0.06468601	0.453	9255	tags=69%, list=17%, signal=83%
0.06580048	0.463	4721	tags=42%, list=9%, signal=46%
0.06681035	0.482	10167	tags=49%, list=18%, signal=60%
0.065451235	0.483	8146	tags=51%, list=15%, signal=60%
0.06838529	0.501	10128	tags=50%, list=18%, signal=61%
0.06795005	0.506	4370	tags=42%, list=8%, signal=45%
0.06691184	0.507	6822	tags=41%, list=12%, signal=47%
0.071682826	0.525	9663	tags=70%, list=17%, signal=84%
0.07664732	0.549	3686	tags=40%, list=7%, signal=42%
0.07706614	0.556	9098	tags=42%, list=16%, signal=49%
0.07541958	0.556	6808	tags=50%, list=12%, signal=57%
0.0999883	0.628	8139	tags=56%, list=15%, signal=66%
0.10032569	0.634	12994	tags=59%, list=24%, signal=77%
0.10537875	0.656	9051	tags=51%, list=16%, signal=61%
0.10419618	0.657	18040	tags=76%, list=33%, signal=113%
0.11425807	0.681	9595	tags=47%, list=17%, signal=57%
0.113002375	0.681	8131	tags=33%, list=15%, signal=38%
0.11194036	0.684	8655	tags=47%, list=16%, signal=55%
0.11284394	0.693	10768	tags=63%, list=19%, signal=78%

0.115702495	0.703	8077	tags=56%, list=15%, signal=66%
0.116029695	0.71	10107	tags=50%, list=18%, signal=61%
0.11925669	0.721	5750	tags=34%, list=10%, signal=38%
0.12405768	0.733	10483	tags=46%, list=19%, signal=56%
0.12366667	0.736	7654	tags=71%, list=14%, signal=82%
0.12268704	0.737	7316	tags=47%, list=13%, signal=54%
0.12868176	0.747	6585	tags=52%, list=12%, signal=59%
0.12786205	0.748	8649	tags=38%, list=16%, signal=46%
0.14413173	0.788	10372	tags=43%, list=19%, signal=53%
0.14641471	0.794	8669	tags=44%, list=16%, signal=52%
0.1444689	0.794	7947	tags=39%, list=14%, signal=46%
0.15028894	0.803	8418	tags=47%, list=15%, signal=56%
0.17013136	0.834	10562	tags=45%, list=19%, signal=56%
0.17980656	0.85	8021	tags=50%, list=15%, signal=58%
0.18184517	0.855	8701	tags=29%, list=16%, signal=34%
0.18629381	0.863	10070	tags=45%, list=18%, signal=55%
0.18800922	0.864	10533	tags=54%, list=19%, signal=66%
0.18677704	0.865	11431	tags=52%, list=21%, signal=66%
0.18676484	0.868	6363	tags=36%, list=12%, signal=41%
0.18632151	0.869	11952	tags=56%, list=22%, signal=71%
0.20473106	0.885	10204	tags=34%, list=18%, signal=42%
0.21646819	0.896	4076	tags=24%, list=7%, signal=26%
0.22881117	0.904	9120	tags=46%, list=17%, signal=55%
0.23548622	0.91	9482	tags=40%, list=17%, signal=49%
0.24955092	0.924	9013	tags=36%, list=16%, signal=43%
0.2581165	0.931	8021	tags=33%, list=15%, signal=39%
0.25500298	0.931	7577	tags=33%, list=14%, signal=38%
0.25224388	0.931	8649	tags=41%, list=16%, signal=48%
0.250633	0.933	6020	tags=32%, list=11%, signal=36%
0.24762636	0.933	10152	tags=35%, list=18%, signal=42%
0.24821806	0.934	8418	tags=37%, list=15%, signal=43%
0.24550897	0.934	4004	tags=24%, list=7%, signal=25%
0.2581551	0.936	9036	tags=39%, list=16%, signal=46%
0.26365498	0.937	8056	tags=34%, list=15%, signal=40%
0.26466295	0.939	8418	tags=40%, list=15%, signal=47%
0.2757714	0.943	8616	tags=33%, list=16%, signal=39%
0.30252075	0.952	9310	tags=52%, list=17%, signal=63%
0.30123925	0.952	9135	tags=37%, list=17%, signal=45%
0.29904416	0.953	16833	tags=65%, list=30%, signal=93%
0.30598557	0.958	3123	tags=24%, list=6%, signal=25%
0.31721312	0.96	16131	tags=64%, list=29%, signal=90%
0.31407726	0.96	23297	tags=68%, list=42%, signal=117%
0.31404477	0.961	7769	tags=32%, list=14%, signal=37%
0.3244631	0.966	8600	tags=38%, list=16%, signal=45%
0.32358593	0.967	5475	tags=37%, list=10%, signal=41%
0.3279272	0.971	12719	tags=41%, list=23%, signal=54%
0.3295343	0.972	3334	tags=24%, list=6%, signal=26%
0.33153582	0.973	4570	tags=26%, list=8%, signal=28%
0.3302665	0.974	10070	tags=43%, list=18%, signal=52%
0.34025836	0.979	8720	tags=37%, list=16%, signal=44%
0.3376965	0.98	13215	tags=32%, list=24%, signal=43%
0.34552464	0.981	10128	tags=37%, list=18%, signal=45%
0.36272356	0.985	7727	tags=28%, list=14%, signal=33%

0.39110017	0.988	13215	tags=35%, list=24%, signal=46%
0.39358208	0.989	3675	tags=21%, list=7%, signal=22%
0.39721477	0.989	3699	tags=15%, list=7%, signal=16%
0.3992318	0.989	3643	tags=20%, list=7%, signal=21%
0.43940118	0.99	13215	tags=32%, list=24%, signal=42%
0.5037036	0.997	8669	tags=31%, list=16%, signal=37%
0.5000081	0.997	7356	tags=40%, list=13%, signal=46%
0.4964539	0.997	13080	tags=38%, list=24%, signal=50%
0.49701244	0.997	3482	tags=22%, list=6%, signal=24%
0.5792396	0.998	9270	tags=23%, list=17%, signal=28%
0.64798063	1	6594	tags=24%, list=12%, signal=28%
0.7125312	1	9371	tags=32%, list=17%, signal=38%
0.74822855	1	23297	tags=61%, list=42%, signal=105%
