# Supplementary material for: Uncovering the Immune Cell Infiltration Landscape in Low-Grade Glioma for Aiding Immunotherapy
Source: J Oncol. 2022 Mar 11;2022:3370727. doi: 10.1155/2022/3370727 (PMC8933094; doi:10.1155/2022/3370727)
Supplement: Supplementary Materials — Supplementary Table 1: the list of ICI gene signatures A and B. Supplementary Table 2: GSEA results in high ICI score group. Supplementary Table 3: GSEA results in low ICI score group. Supplementary Table 4: DEGs between high and low ICI score groups. Supplementary Figure 1: validation of the three ICI subtypes for LGG in the CGGA-LGG dataset. (A–C) Unsupervised clustering analysis for validating the classifications of three ICI subtypes. (A) Consensus cumulative distribution function graph. (B) Delta area plot. (C) Heatmap for consensus matrix when k = 3. (D) Heatmap of tumor-infiltrating immune cells in different clinical phenotypes and ICI subtypes. (E) PCA plots for confirming the classification patterns of the ICI subtypes. (F) Kaplan-Meier curves for OS of LGG patients in the three ICI subtypes. [file 3370727.f1.zip › 3370727.f1/Supplementary table 4.pdf]

Supplementary table 4. DEGs between high and low ICI score groups.

id	logFC	AveExpr	t	P.Value
VSNL1	-3.44633838	4.710443342	-19.74597668	9.94E-65
NEFL	-3.435882699	4.07517438	-19.73545453	1.12E-64
SLC17A7	-3.425641678	5.173447874	-17.51905324	4.97E-54
CAMK2A	-3.33533302	4.575266737	-19.82408143	4.15E-65
NRGN	-3.280675321	6.521289702	-18.6752214	1.48E-59
NEFM	-3.218519539	3.276798343	-21.56309883	1.40E-73
PACSIN1	-3.159551166	3.979940353	-19.97328662	7.84E-66
SNAP25	-3.078547245	7.311049556	-21.60986547	8.29E-74
CPLX2	-3.059565516	5.630993488	-20.55258793	1.19E-68
GRIN1	-3.0350676	4.332399228	-21.28285325	3.28E-72
SNCB	-3.031570811	5.859690047	-21.86161492	4.88E-75
SULT4A1	-2.985392566	3.910645266	-20.33699089	1.34E-67
SYT1	-2.969369842	4.965739845	-19.38055646	5.85E-63
GABRA1	-2.961399953	2.77876226	-22.06135314	5.16E-76
GABRG2	-2.960692559	3.435768835	-23.04759474	7.81E-81
DDN	-2.960058546	3.519600162	-21.8127168	8.46E-75
CCK	-2.918207692	3.586762215	-19.42693848	3.49E-63
CRYM	-2.870308414	3.581601317	-18.53625969	6.89E-59
TMEM130	-2.86714287	4.226113535	-21.94667273	1.87E-75
HPCA	-2.851233917	4.556693146	-18.17681368	3.64E-57
L1CAM	-2.8405509	4.415696639	-18.84094751	2.36E-60
CHGA	-2.818887389	5.403138731	-19.82711843	4.02E-65
RGS4	-2.810819356	3.578515997	-19.2098425	3.92E-62
SYT4	-2.76154193	3.347473756	-21.16239146	1.27E-71
SYT13	-2.753549178	3.075101709	-23.31686538	3.78E-82
CREG2	-2.74256139	2.706873497	-20.72474162	1.73E-69
MYT1L	-2.729642532	2.754512133	-24.59074781	2.32E-88
RBFOX3	-2.715204894	3.081510629	-23.27749439	5.88E-82
SLC6A17	-2.699583941	3.063676909	-21.91593716	2.65E-75
CALY	-2.674207088	3.160479687	-21.64029871	5.89E-74
STMN2	-2.673908252	5.700477105	-15.14228937	5.69E-43
PVALB	-2.671488777	2.224236632	-20.53902598	1.39E-68
CACNG3	-2.661410166	2.215270807	-22.87259376	5.59E-80
SVOP	-2.615862162	3.046221987	-22.89460048	4.37E-80
SLC12A5	-2.611494817	3.362282324	-23.36300926	2.25E-82
FAM163B	-2.587471186	4.40194876	-18.63523721	2.31E-59
CELF4	-2.546018668	3.798583854	-23.82963741	1.19E-84
SYN1	-2.5382608	5.648821209	-23.18450195	1.67E-81
NPTX1	-2.537630858	4.072151879	-18.28459861	1.11E-57
CAMK1G	-2.5362481	2.338925822	-20.43741907	4.34E-68
GNG3	-2.533349685	4.867709267	-15.99389634	7.08E-47
PTPRN	-2.527412303	4.61185871	-19.16432014	6.50E-62
PHYHIP	-2.522281367	5.285644209	-20.4708767	2.98E-68
TESPA1	-2.51676906	1.586829156	-19.57468054	6.72E-64
GABRB2	-2.507704001	2.24144794	-21.14259923	1.58E-71
SYNGR3	-2.498720442	3.931154761	-22.83346827	8.68E-80
SLC30A3	-2.486142217	1.955219347	-20.93468957	1.64E-70
SV2B	-2.48446055	2.319023767	-19.89318255	1.92E-65
SYN2	-2.474921899	4.776217875	-20.24071733	3.93E-67
PTPN5	-2.459981853	3.11102775	-20.9668039	1.14E-70

GDA	-2.448491743	2.660522603	-18.18348837	3.39E-57
RBFOX1	-2.442606346	2.813979328	-20.44645396	3.92E-68
SYNPR	-2.440963676	2.861738076	-17.8048928	2.17E-55
MPPED1	-2.432896281	2.311673212	-20.55856549	1.12E-68
INA	-2.424291748	5.265278243	-16.61762276	8.85E-50
MAP7D2	-2.423194509	2.281128719	-20.47907908	2.72E-68
CHRM1	-2.420041642	3.209824968	-22.39234603	1.24E-77
KCNC2	-2.419121172	1.920075444	-25.81797745	2.57E-94
MAL2	-2.417632979	2.170996764	-20.58453255	8.34E-69
CDK5R2	-2.406122512	3.971258295	-22.54609719	2.20E-78
SLC32A1	-2.387939286	2.244586515	-20.48793095	2.46E-68
IQSEC3	-2.372911538	2.901023452	-23.54866672	2.79E-83
RPH3A	-2.349874183	4.153372733	-16.66321176	5.41E-50
GABRA5	-2.33778454	2.421165764	-18.76742545	5.33E-60
PCP4L1	-2.334758724	2.726367164	-20.96924779	1.11E-70
SYT7	-2.33285702	3.653784131	-20.37377362	8.85E-68
PRKCG	-2.312543419	2.77021295	-17.31443514	4.64E-53
RAB3C	-2.296003796	3.107233053	-23.8151029	1.40E-84
PHF24	-2.294878275	2.975801343	-19.07224617	1.81E-61
GAD2	-2.284892823	2.101898151	-20.41090569	5.84E-68
VSTM2A	-2.284052666	3.711923485	-16.4548354	5.10E-49
ICAM5	-2.283664999	2.811522449	-21.23260923	5.76E-72
TMEM155	-2.283058404	1.963503532	-21.42909246	6.33E-73
RAB3A	-2.276902514	5.801494419	-24.57444088	2.79E-88
AL022313.4	-2.266261306	3.631804774	-16.506515	2.93E-49
NEURL1	-2.260952245	2.717664389	-23.62955481	1.12E-83
FXYD7	-2.258513025	4.280066132	-15.81891254	4.56E-46
PCSK2	-2.25541405	3.603509543	-17.63614113	1.38E-54
TAC1	-2.253080957	2.64988367	-15.90553114	1.81E-46
SLC4A10	-2.237780253	2.879829748	-21.46598597	4.18E-73
DNM1	-2.226059251	5.464143043	-18.61602143	2.85E-59
ENC1	-2.208827989	5.41776127	-16.67914536	4.56E-50
SLC6A7	-2.203941808	1.434683349	-21.61830955	7.54E-74
C1QL3	-2.194202703	1.548881134	-19.78303277	6.57E-65
NEFH	-2.185929077	3.015848758	-18.8767506	1.59E-60
CAMKV	-2.178333672	4.035966544	-18.11194098	7.45E-57
CABP1	-2.177299925	3.498800813	-21.6823205	3.67E-74
ATP2B3	-2.168483394	2.109780042	-23.12007467	3.45E-81
HRH3	-2.149510961	2.751171562	-20.05090114	3.29E-66
SYT5	-2.147089576	3.188505157	-19.84724253	3.21E-65
CLEC2L	-2.132620878	2.047770674	-20.99258629	8.55E-71
SCRT1	-2.122339914	3.577076614	-16.95680128	2.26E-51
DOC2A	-2.122210098	2.753246008	-21.30509678	2.55E-72
CHGB	-2.120111516	6.311396357	-16.12084487	1.83E-47
SCN3B	-2.10843998	4.684209342	-18.2646397	1.38E-57
EPHB6	-2.096790174	3.454787226	-17.91218794	6.70E-56
TMEM151B	-2.096200478	4.182986252	-19.51876267	1.25E-63
CHD5	-2.088896311	2.770136361	-19.0200048	3.23E-61
ACTL6B	-2.083255558	4.812883257	-16.0290341	4.87E-47
NSG2	-2.079488295	7.056582675	-14.00270748	7.14E-38
C11orf87	-2.072672294	1.622973734	-20.23875032	4.02E-67
STX1A	-2.072546985	4.627562566	-20.24171592	3.89E-67
SLC8A2	-2.072442198	3.404180429	-20.19610527	6.48E-67

TMEM132D	-2.064985731	1.546116321	-22.76487049	1.88E-79
WSCD2	-2.044020295	2.167579185	-21.13333444	1.76E-71
DMTN	-2.042024596	5.262550531	-19.51567633	1.30E-63
4-Mar	-2.041816013	2.44531126	-17.08174943	5.83E-52
RBP4	-2.038395984	2.187085792	-19.20152802	4.30E-62
EEF1A2	-2.033109515	6.847634338	-18.84087627	2.36E-60
CCKBR	-2.03100879	1.644473434	-22.14692199	1.97E-76
SH2D5	-2.029590794	1.856595387	-22.05319857	5.65E-76
SNCG	-2.029372489	5.51669316	-16.11751515	1.89E-47
CACNG2	-2.027973629	2.783469646	-15.87480462	2.52E-46
KCNS1	-2.022658562	1.328691858	-18.98421156	4.81E-61
CPLX1	-2.02065654	5.17344631	-19.12732317	9.81E-62
KCNJ4	-2.01524865	3.086847754	-16.82645604	9.28E-51
MIR7-3HG	-2.014198057	1.921264732	-20.59068147	7.78E-69
GALNT17	-1.998137744	3.619637134	-15.49274245	1.44E-44
SHISAL1	-1.983492928	3.028799823	-17.63317841	1.43E-54
ATP8A2	-1.982228318	2.12043913	-22.52835459	2.69E-78
GLT1D1	-1.976459832	2.10216678	-21.40704888	8.11E-73
PPP2R2C	-1.975570387	4.753172887	-16.1294232	1.67E-47
PSD	-1.971263657	5.659691607	-20.77221115	1.01E-69
GABRD	-1.970119206	4.431714309	-15.2632334	1.60E-43
CBLN2	-1.969164889	2.179956954	-20.52969067	1.54E-68
RFPL1S	-1.966707874	2.283240653	-21.93217763	2.21E-75
ARHGDIG	-1.965154132	5.027706133	-18.5104647	9.17E-59
VSTM2L	-1.963901342	4.123514799	-16.86348972	6.22E-51
NEUROD6	-1.961460438	1.244506719	-18.94252793	7.64E-61
HCN1	-1.961361724	1.42246451	-21.34243476	1.68E-72
FAM19A2	-1.960464815	2.754663874	-22.65922085	6.17E-79
NGB	-1.940562336	1.321176519	-19.22809094	3.20E-62
OLFM3	-1.936915086	1.62567463	-19.94150346	1.12E-65
NGEF	-1.935480413	4.3196525	-14.95615098	3.97E-42
AC104072.1	-1.93539123	2.450917506	-15.06503265	1.28E-42
KCNT1	-1.934837237	1.783339173	-23.92129447	4.24E-85
OGDHL	-1.931359484	3.101223008	-17.53075938	4.38E-54
CPNE6	-1.929336959	2.843000819	-14.26463597	4.97E-39
CAMK2B	-1.927772834	4.865804514	-18.1128615	7.37E-57
NELL2	-1.923885086	4.770483907	-13.49404411	1.19E-35
SHANK1	-1.919583946	3.034903548	-20.57821254	8.95E-69
TRIM67	-1.919275797	2.609384967	-11.28243036	1.76E-26
NELL1	-1.91752546	2.024194086	-17.93076003	5.46E-56
MIR770	-1.916364653	3.39195752	-14.80589665	1.89E-41
NAPB	-1.912267523	5.465884321	-20.74172475	1.43E-69
PTER	-1.912228898	2.347780683	-17.54817627	3.62E-54
WNT10B	-1.909925108	1.565104394	-22.52400953	2.83E-78
SLC26A4-AS1	-1.906444513	1.612194422	-17.60103698	2.03E-54
C1orf115	-1.902933333	3.695597602	-19.27002834	2.00E-62
RIMS2	-1.902164086	2.675594543	-20.98430808	9.38E-71
TUNAR	-1.898370517	1.622446781	-23.02280388	1.03E-80
SRRM4	-1.896377243	2.078658685	-22.35511962	1.89E-77
MICAL2	-1.895004275	2.953022103	-18.2409365	1.80E-57
SNCA	-1.887364844	4.407660514	-17.27685516	6.99E-53
BEX5	-1.879426612	4.315023504	-15.48556167	1.55E-44
NNAT	-1.878673801	4.508905239	-10.42412082	3.57E-23

OLFM1	-1.878078369	7.123167298	-20.94927286	1.39E-70
CBLN4	-1.873623554	1.455196035	-16.77919265	1.55E-50
ZCCHC12	-1.873211424	2.456187602	-16.0779501	2.89E-47
CALB2	-1.870300118	2.882392276	-14.53694787	3.04E-40
PNOC	-1.86942533	1.889375439	-14.51545736	3.80E-40
RIMS1	-1.855269979	2.43525072	-22.47081861	5.14E-78
GJB6	-1.855203998	2.290109953	-11.61320373	8.52E-28
CAMK4	-1.852465574	2.23594529	-19.90340441	1.71E-65
STAC2	-1.852358178	2.901015778	-14.29770596	3.54E-39
ST8SIA3	-1.852048241	3.12578806	-17.08053797	5.91E-52
PRKCB	-1.850735541	4.246441039	-18.78145358	4.56E-60
RTN4RL1	-1.845384807	1.799357458	-20.2207497	4.91E-67
AK5	-1.84253293	4.313500861	-13.13372989	4.22E-34
SST	-1.840612473	3.968138457	-11.69147139	4.13E-28
MFSD4A	-1.83873728	3.404581036	-21.90740793	2.92E-75
TAC3	-1.833454342	1.781191731	-17.52456502	4.68E-54
HS6ST3	-1.83173063	1.758925829	-17.2799202	6.76E-53
EMX1	-1.827138151	1.353192299	-20.80983457	6.65E-70
SNAP91	-1.826204042	5.332665955	-17.75099818	3.93E-55
HPCAL4	-1.823422846	4.808050011	-13.81521372	4.75E-37
AMPH	-1.809274284	4.416879605	-21.16174548	1.28E-71
FAM19A1	-1.809199824	2.124809322	-16.4845511	3.71E-49
TNNT1	-1.804536348	2.562925601	-17.06061346	7.34E-52
RIT2	-1.802169182	2.608297651	-13.54977965	6.81E-36
NRSN1	-1.800925357	5.038104888	-16.00053367	6.60E-47
PRSS3	-1.796804261	2.409409611	-20.41690552	5.46E-68
ELAVL2	-1.793851743	3.314139243	-19.95237723	9.90E-66
FBXL16	-1.792932031	6.372786848	-20.89303961	2.61E-70
RGS7	-1.789024993	3.467389338	-20.31495073	1.71E-67
TBR1	-1.784450879	1.441325483	-19.56758672	7.28E-64
NRIP3	-1.780408378	2.761543475	-17.08047666	5.91E-52
TAGLN3	-1.779095633	6.451972445	-18.7172744	9.29E-60
PDYN	-1.779022671	2.118998731	-11.00514295	2.14E-25
CHN1	-1.778403195	6.687059257	-17.69843829	6.99E-55
RASAL1	-1.777076925	2.029588014	-15.69637496	1.67E-45
TCEAL6	-1.776157211	3.503826032	-13.55911054	6.20E-36
KCNH3	-1.775957468	3.083066761	-15.98721191	7.61E-47
NPM2	-1.771749441	2.596383624	-17.99805283	2.61E-56
RIMBP2	-1.770436588	2.14838457	-18.6749084	1.49E-59
NEUROD2	-1.769467197	1.515434647	-19.165957	6.38E-62
ASIC2	-1.767179623	1.824640417	-21.9563526	1.68E-75
AMER3	-1.765478187	2.17083197	-17.99245764	2.77E-56
JPH3	-1.763309807	4.964681639	-15.77292151	7.42E-46
UBE2QL1	-1.761397427	3.693554712	-21.99505437	1.09E-75
ADAM11	-1.759122917	2.84181492	-20.48679203	2.49E-68
SCN2A	-1.746086269	3.053206353	-21.5372304	1.88E-73
PDE2A	-1.7438631	5.139688789	-18.55650658	5.51E-59
KCNK3	-1.740414738	2.706591309	-15.973947	8.76E-47
TMEM271	-1.739945813	3.027100552	-17.2470287	9.67E-53
SEZ6L2	-1.737966287	5.924191324	-15.69422578	1.71E-45
VIP	-1.735152901	1.527645845	-19.76676376	7.88E-65
CNNM1	-1.734315755	1.840662709	-23.70548574	4.79E-84
CUX2	-1.734295781	2.556452774	-16.02533977	5.07E-47

GABRB3	-1.732565703	4.604712237	-16.04153995	4.26E-47
KCNIP2	-1.732134895	5.138107662	-13.45892545	1.69E-35
SCN2B	-1.732055688	3.47861371	-19.48704228	1.79E-63
DLGAP1-AS4	-1.732020609	1.409637602	-19.5188299	1.25E-63
CELF3	-1.730536586	4.871789543	-15.82447396	4.29E-46
MRAP2	-1.729925599	2.214188897	-16.2201261	6.33E-48
KCNV1	-1.727676069	0.979582899	-20.62202457	5.47E-69
SMIM10L2B	-1.723541349	3.061026208	-22.1984888	1.10E-76
ISLR2	-1.720983518	1.825841073	-19.02956051	2.91E-61
SYP	-1.719529686	7.131689101	-21.61615882	7.72E-74
AGAP2	-1.710235776	5.140804031	-18.8142725	3.17E-60
SHC3	-1.708814919	4.459584158	-13.59060126	4.53E-36
AC122707.1	-1.708267961	2.040404451	-16.44668076	5.57E-49
CNTNAP2	-1.707608061	3.427210267	-16.8203757	9.91E-51
LRTM2	-1.704744729	2.033077079	-16.17099158	1.07E-47
STXBP1	-1.701364609	6.688376806	-24.44092831	1.24E-87
FRRS1L	-1.701078393	3.47478479	-18.0054497	2.40E-56
ZMAT4	-1.692970202	1.734684361	-20.63986392	4.48E-69
KCNAB2	-1.692448727	5.091988662	-18.71179466	9.87E-60
PITPNM3	-1.689767595	2.83655895	-17.702365	6.69E-55
CPNE7	-1.687629419	1.667163516	-20.71917049	1.84E-69
ARHGAP44	-1.686890399	3.089460331	-21.5610899	1.43E-73
DLGAP3	-1.686620283	3.286919185	-19.83433503	3.70E-65
HS3ST2	-1.686094114	2.783527853	-14.65855679	8.69E-41
BICDL1	-1.684135012	2.203605578	-22.02969737	7.36E-76
BSN	-1.682240983	3.434349119	-21.88310569	3.83E-75
PTPRR	-1.681885635	1.477600415	-21.19162477	9.13E-72
CAMKK1	-1.677734808	3.83753454	-16.87607549	5.42E-51
GPR22	-1.671419166	1.246697639	-20.00087531	5.76E-66
HTR5A	-1.671291747	1.055497523	-23.65485076	8.46E-84
ZFR2	-1.666560305	1.747305623	-16.59925152	1.08E-49
TUBA4A	-1.662919906	4.927393198	-14.64794043	9.69E-41
LINC00507	-1.660841608	0.771689536	-18.39836944	3.16E-58
RASD2	-1.659067601	2.829054312	-18.98210691	4.92E-61
AL356479.1	-1.654226623	1.181592388	-17.45694575	9.80E-54
STX1B	-1.649256501	5.255029797	-21.33403108	1.84E-72
UNC5A	-1.64485557	4.228994687	-17.26748762	7.74E-53
NRXN3	-1.643206472	2.723617989	-17.30843067	4.95E-53
PRMT8	-1.641171489	1.403677054	-23.15722675	2.27E-81
CPNE9	-1.640379722	1.476817754	-21.3754533	1.16E-72
CHRNB2	-1.637643322	3.461596629	-18.54426987	6.31E-59
ST6GALNAC5	-1.636678663	1.535652043	-17.16240744	2.43E-52
GABRA4	-1.635690649	1.220455547	-20.71204064	1.99E-69
CARMIL2	-1.632985359	1.932300083	-24.85433636	1.21E-89
WIF1	-1.630580438	1.682380649	-10.80500548	1.27E-24
SOHLH1	-1.62265905	1.262048298	-19.03147807	2.85E-61
AP003355.2	-1.6226277	0.989295196	-21.14460577	1.55E-71
SLC7A14	-1.619604511	3.226612802	-16.50679136	2.92E-49
FBXO41	-1.619430583	3.859036951	-22.89673121	4.26E-80
STYK1	-1.617554711	0.983378286	-23.94393072	3.29E-85
ITPKA	-1.617539872	3.363134471	-18.14930313	4.93E-57
KIF5A	-1.61544615	6.869203843	-16.26195453	4.04E-48
CACNG8	-1.614555481	3.036052758	-19.76615056	7.93E-65

FRMPD4	-1.612335286	1.322032477	-20.11273561	1.65E-66
UNC13A	-1.612209455	4.504513099	-18.51241993	8.97E-59
RTN4R	-1.609584094	3.668288932	-18.33630043	6.28E-58
SOWAHA	-1.608801924	2.80274538	-14.98207597	3.03E-42
5-Sep	-1.608479833	5.930081296	-22.88623134	4.80E-80
GABRA3	-1.607307513	4.15508506	-14.81965942	1.64E-41
PNMA6F	-1.598860055	1.093088995	-19.10408815	1.27E-61
PRKAR1B	-1.59550773	6.595668655	-20.19591744	6.49E-67
AL049749.1	-1.581107924	2.328380037	-13.32581692	6.32E-35
SHISA9	-1.579491052	2.871433692	-13.2747501	1.05E-34
NMNAT2	-1.576828783	4.903803413	-15.18879096	3.50E-43
CTXN3	-1.575651542	0.796419544	-18.40127313	3.06E-58
DYNC1I1	-1.572942253	4.256074459	-16.58825713	1.21E-49
PCSK1	-1.566818009	2.40317822	-12.64517835	4.95E-32
RGS7BP	-1.564472558	2.69150782	-16.46425916	4.61E-49
HIPK4	-1.563322353	1.03499016	-20.10910871	1.71E-66
CARTPT	-1.561967372	0.860271587	-12.64654012	4.88E-32
PART1	-1.555078059	1.451897159	-18.61801966	2.79E-59
RALYL	-1.551553716	2.824655397	-15.82667486	4.20E-46
NCDN	-1.55085088	6.533998684	-17.72114913	5.45E-55
CKMT1A	-1.550334044	1.996249766	-22.88348419	4.95E-80
AC008780.2	-1.549670325	1.049329737	-18.66067089	1.74E-59
GABRA2	-1.549570783	2.30059136	-12.58474089	8.87E-32
RAP1GAP2	-1.547210367	3.245203938	-17.69110568	7.57E-55
KRT222	-1.545703227	1.098882431	-23.15211767	2.41E-81
TNNT2	-1.53854649	0.934804373	-19.6108144	4.49E-64
CYGB	-1.537315566	2.405542492	-16.45537848	5.07E-49
ATP1A3	-1.534440177	7.330322214	-15.55081516	7.78E-45
CACNA2D3	-1.533238262	2.570055683	-17.28851852	6.15E-53
RXFP1	-1.531566034	1.173586336	-20.72516445	1.72E-69
LHX6	-1.531464182	1.536074806	-19.47363719	2.08E-63
GRIN3A	-1.530918656	1.9928779	-14.08932866	2.96E-38
RYR2	-1.529423901	1.197992025	-18.61579252	2.86E-59
NAP1L2	-1.524934759	4.778579379	-18.06661343	1.23E-56
PAK3	-1.524542886	3.235053899	-18.94485455	7.45E-61
GRIN2B	-1.52083509	1.357432826	-19.58362836	6.09E-64
SERPINI1	-1.518696755	5.358546116	-12.49492725	2.10E-31
MATK	-1.518634594	3.102909845	-15.42085359	3.06E-44
KCNK1	-1.517690963	3.877305868	-13.57442084	5.32E-36
SLC6A15	-1.514476339	1.848985108	-14.45784717	6.87E-40
KCNC1	-1.512685646	3.181400346	-20.13815948	1.24E-66
RIMS4	-1.512529875	4.022465361	-14.6097238	1.44E-40
C3orf80	-1.511823987	1.242837222	-19.2794739	1.80E-62
ADCY1	-1.51096717	3.036036889	-13.03661867	1.09E-33
CA7	-1.509927409	1.12465968	-20.32481158	1.53E-67
PAK1	-1.509770831	4.749476236	-15.21812096	2.57E-43
CDH18	-1.508448542	2.476219822	-14.89809032	7.26E-42
KCNAB1	-1.507904549	3.282761776	-19.06329423	2.00E-61
JAKMIP1	-1.50779487	3.385628131	-15.07278742	1.18E-42
KCNA1	-1.50775151	1.478505263	-17.63455783	1.41E-54
SYT16	-1.507544697	2.007267422	-17.94528687	4.66E-56
KCNJ3	-1.507346434	2.31368212	-15.11720036	7.40E-43
CKMT1B	-1.505756769	2.17501297	-20.93403478	1.65E-70

SLC35F3	-1.497161644	1.498255896	-21.28884944	3.06E-72
VGF	-1.496632789	4.285888395	-8.106514581	3.97E-15
DOK6	-1.493315486	2.765136604	-18.53191824	7.23E-59
PPP1R14C	-1.492316964	2.972945359	-14.35699197	1.93E-39
VWA5B2	-1.490843363	2.573030479	-20.88828094	2.76E-70
CLVS1	-1.489200278	1.848216254	-15.99847993	6.75E-47
NRN1	-1.48917664	5.434255565	-12.68169162	3.48E-32
NPTXR	-1.487696262	6.176189596	-18.40090618	3.08E-58
CHRM3	-1.486564112	1.444043296	-14.97320298	3.32E-42
RUNDC3A	-1.485674833	6.263431226	-17.78209859	2.79E-55
RSPO2	-1.48457217	1.858825666	-14.36390043	1.80E-39
STXBP6	-1.479421763	3.497951723	-18.52513438	7.79E-59
CA11	-1.477216772	6.491001861	-17.58912543	2.31E-54
SERTM1	-1.476620116	1.196936349	-16.0237198	5.16E-47
CACNA1I	-1.473802711	1.498554157	-21.13667337	1.69E-71
DIO2	-1.473022367	3.248579936	-11.9984059	2.36E-29
AC062021.1	-1.472587175	3.326402149	-7.781784161	4.07E-14
KLK7	-1.472281593	0.985939589	-16.78456785	1.46E-50
HTR1A	-1.468049484	1.259859459	-16.77381523	1.64E-50
HTR2A	-1.467484288	2.079082272	-16.91913396	3.40E-51
CXCL14	-1.463226753	4.204287645	-8.336018991	7.34E-16
NSG1	-1.460893097	4.039352057	-13.28055434	9.90E-35
CPNE4	-1.45683663	1.910193902	-13.51778005	9.37E-36
CDH9	-1.453428792	1.082134335	-21.8798098	3.98E-75
GUCY1B1	-1.453078701	4.233126098	-19.19817411	4.46E-62
PCLO	-1.450030859	1.39842788	-19.50428619	1.47E-63
SCN8A	-1.446838165	2.781091615	-21.30492957	2.56E-72
AL359764.1	-1.446031646	2.380368141	-14.34040572	2.29E-39
ATCAY	-1.44560649	6.382594353	-11.23901275	2.61E-26
SCG5	-1.444299682	6.907926484	-12.94455111	2.70E-33
UNC13C	-1.442450574	1.167929205	-17.69298779	7.42E-55
ENTPD3	-1.43962524	1.537688167	-15.30873821	9.95E-44
PNCK	-1.436040252	3.975910616	-16.54078966	2.02E-49
CDH22	-1.432713044	3.046964809	-14.19914517	9.69E-39
ERICH3	-1.431437257	1.644956176	-13.29417964	8.65E-35
NPTX2	-1.430708895	3.534566483	-8.777146515	2.60E-17
ITPR1	-1.429118242	2.830761818	-14.90635693	6.66E-42
HECW1	-1.429074364	2.134711969	-18.58319432	4.10E-59
GPR83	-1.428770485	1.01140192	-20.38434079	7.86E-68
STXBP5L	-1.428017774	2.052510342	-16.54634753	1.91E-49
MMD2	-1.427838997	4.746546307	-11.44878237	3.86E-27
LINC00599	-1.427663286	3.073951032	-12.40931737	4.78E-31
FNDC9	-1.427314469	1.839938802	-13.74248694	9.87E-37
CELF5	-1.427144077	4.363312611	-14.47119006	5.99E-40
XKR7	-1.425842263	1.721550523	-13.72580046	1.17E-36
CA10	-1.424496046	4.652220326	-10.03181363	1.02E-21
GAD1	-1.4244277	5.04729244	-12.90046958	4.15E-33
PCDH8	-1.423702997	2.308872913	-14.50775904	4.11E-40
AKAP5	-1.421318761	1.811405869	-20.9248568	1.83E-70
CYP4X1	-1.421258936	1.619013545	-20.57610714	9.16E-69
AL139246.5	-1.41708764	1.715338165	-15.39452415	4.04E-44
PCP4	-1.414352576	3.728922181	-11.1836483	4.31E-26
PENK	-1.413535827	2.428998509	-10.26802697	1.37E-22

FAM81A	-1.407864029	3.36375241	-15.71256234	1.41E-45
KCNS2	-1.40472376	0.872765738	-20.03111221	4.10E-66
KCNJ9	-1.40470567	4.833448092	-15.75206031	9.26E-46
AC104024.2	-1.402522188	0.789528635	-19.65351353	2.79E-64
GRIN2A	-1.402079111	2.007591316	-12.5768177	9.57E-32
DACH2	-1.401667857	1.988985555	-13.18950684	2.43E-34
MIR124-2HG	-1.401064464	2.310192944	-16.49367773	3.36E-49
TENM2	-1.39983635	1.583012283	-15.79269186	6.02E-46
PPFIA2	-1.399509215	3.104105342	-16.56257058	1.60E-49
PTPRT	-1.398725897	3.306314478	-12.54344198	1.32E-31
RAB15	-1.398656748	4.303693601	-17.87977811	9.56E-56
DIRAS2	-1.398064459	4.321966666	-12.87498015	5.32E-33
LY86-AS1	-1.397653426	0.855866105	-19.64888895	2.94E-64
HTR5A-AS1	-1.396714726	0.798244545	-20.29962865	2.03E-67
MGAT5B	-1.396382911	3.907969987	-16.57767932	1.36E-49
ANK3	-1.395580847	2.899040987	-15.37105501	5.17E-44
MAP3K9	-1.395527329	1.664763475	-20.37028453	9.20E-68
SEZ6L	-1.391702731	6.613231052	-10.89037062	5.97E-25
RNU6-353P	-1.390232155	1.583180824	-18.58068205	4.22E-59
ABCC8	-1.387934411	3.812021583	-11.04120087	1.55E-25
CEND1	-1.374289301	6.694179481	-18.06629351	1.23E-56
COL26A1	-1.373102855	2.452727854	-14.46943235	6.09E-40
DLX1	-1.372653601	1.74785298	-13.17810479	2.72E-34
OPCML	-1.371610388	4.107569768	-13.20570818	2.07E-34
TTC9B	-1.37157361	3.775064053	-15.38913433	4.28E-44
RCAN2	-1.369136808	4.530710872	-13.00813026	1.45E-33
ABLIM2	-1.368351341	3.316602913	-20.03081183	4.12E-66
RAP1GAP	-1.367428655	5.191038435	-17.49431129	6.52E-54
PNMA3	-1.366682015	3.412312205	-14.44805689	7.59E-40
SCG2	-1.366450845	6.440388394	-10.24799119	1.62E-22
ABCG4	-1.365673308	1.425536567	-20.7026803	2.21E-69
SSTR3	-1.364474233	0.842144057	-21.54734165	1.67E-73
PPFIA4	-1.364076755	3.149046451	-18.30201081	9.16E-58
CNTN4	-1.363270493	1.696203134	-18.65980084	1.76E-59
KIAA0513	-1.358930544	4.78952379	-19.218761	3.55E-62
HAR1A	-1.358629594	1.842386881	-17.36245729	2.75E-53
RIMS3	-1.354178297	4.006455572	-13.11661474	4.99E-34
ASPHD1	-1.352519991	5.412616965	-14.39338027	1.33E-39
LINC01007	-1.35171576	0.575050235	-15.98420448	7.85E-47
JPH4	-1.350709762	5.922067047	-13.90863605	1.85E-37
CHRDL1	-1.348657066	3.916955308	-10.36282949	6.06E-23
SYNPO	-1.348562412	4.251086397	-10.73405505	2.38E-24
AP000843.1	-1.347536711	1.297526794	-19.50119378	1.53E-63
NCS1	-1.346344513	6.186055638	-17.27954505	6.79E-53
CACNA1B	-1.345056415	1.800170548	-14.83894016	1.34E-41
LINC00943	-1.341962438	1.092219829	-18.9114739	1.08E-60
RTP5	-1.341229731	4.348588559	-9.368025806	2.46E-19
SHANK2	-1.340277402	3.015342268	-14.29392844	3.68E-39
NECAB2	-1.338791502	4.547682964	-14.79684754	2.08E-41
LYPD8	-1.338268331	0.83952069	-19.30889084	1.30E-62
SLC17A6	-1.336508051	1.147972564	-16.52893029	2.30E-49
LY6H	-1.336161491	4.9633935	-10.07757509	6.94E-22
PDE1B	-1.333405811	2.957907412	-15.47339176	1.76E-44

ANKRD34A	-1.331114925	2.184833408	-19.20325253	4.21E-62
TMEM196	-1.330654331	1.780499563	-12.26260085	1.94E-30
AC012213.4	-1.329603236	2.23219508	-12.19323601	3.74E-30
RPRML	-1.328794236	1.186615985	-17.51449717	5.23E-54
GOT1	-1.326439281	6.001144589	-21.46684206	4.14E-73
GREM2	-1.324587231	1.15606272	-19.05937572	2.09E-61
MEPE	-1.324453834	0.903523403	-15.62499722	3.56E-45
GFOD1	-1.32434797	2.919593369	-21.37566773	1.15E-72
SLC7A4	-1.322361135	1.167452684	-20.71674655	1.89E-69
GRM7	-1.321313829	1.180642413	-17.61159228	1.81E-54
PRDM8	-1.321186975	2.812999663	-14.66172879	8.41E-41
SLC17A8	-1.319860936	1.493413551	-10.23517805	1.81E-22
HSPB3	-1.318831947	1.154841493	-16.17139221	1.07E-47
KCNJ11	-1.31827232	3.996882995	-10.50230292	1.81E-23
ADRA1B	-1.316941356	2.34808589	-15.08241603	1.06E-42
LRFN5	-1.31606436	2.265871188	-13.30587355	7.70E-35
SLC1A2	-1.31579808	7.393130606	-11.50404687	2.33E-27
EPHA10	-1.315540231	2.027874671	-17.36002001	2.82E-53
PNMA5	-1.315063246	1.247605099	-17.56006149	3.18E-54
AL021395.1	-1.312804555	1.151152379	-19.18042365	5.43E-62
STAT4	-1.312236283	1.201796564	-16.17845753	9.88E-48
NSF	-1.311776885	5.639023261	-21.28600965	3.16E-72
SSTR2	-1.311265332	3.366902716	-11.34118051	1.03E-26
GJD2	-1.31092102	1.076275146	-15.94743497	1.16E-46
CLVS2	-1.30832389	2.190206257	-12.06677286	1.24E-29
GRM2	-1.306856217	1.162623452	-21.00356545	7.56E-71
KCNN1	-1.304160345	3.492672945	-17.70718294	6.35E-55
IDS	-1.302715135	6.759693714	-15.44185828	2.46E-44
EPHX4	-1.301519643	2.082987055	-17.72556138	5.19E-55
CACNA1E	-1.299246078	2.154549677	-15.29469	1.15E-43
ANKRD33B	-1.297999566	1.817118842	-17.62421425	1.57E-54
MAPK8IP2	-1.296735012	5.497305483	-18.45840567	1.63E-58
CPNE5	-1.295831699	5.272420052	-12.68005235	3.53E-32
FAM155A	-1.295345048	3.454903426	-13.98776879	8.31E-38
SRRM3	-1.293362061	3.986996716	-11.31315029	1.33E-26
WNT7B	-1.292581309	3.54134316	-9.956196049	1.93E-21
MCHR2	-1.291349305	0.641556652	-18.4564535	1.67E-58
RTN4RL2	-1.290862364	3.489390125	-14.78424535	2.37E-41
SLITRK1	-1.289736138	3.437707842	-12.79957	1.11E-32
AC015468.3	-1.289273912	0.819930689	-17.28274911	6.55E-53
SPTBN2	-1.289104549	5.055893222	-15.95614338	1.06E-46
SYNGR1	-1.287849943	6.075173314	-20.22798205	4.53E-67
CDK5R1	-1.287519363	5.647985724	-15.3093205	9.89E-44
ATP2B2	-1.287277219	4.611103404	-14.99839206	2.56E-42
BRINP1	-1.283701349	4.957390372	-10.58375989	8.90E-24
NECAB1	-1.283479966	3.462155279	-11.65099296	6.01E-28
PPP4R4	-1.277290386	2.472917772	-12.12726655	6.99E-30
SPTB	-1.275111765	1.283685052	-18.79699947	3.84E-60
ASPDH	-1.273448077	3.540278779	-15.64407902	2.91E-45
GOLGA7B	-1.273447886	3.623733183	-13.51891051	9.27E-36
CACNA1G	-1.272524328	2.060913539	-14.26052795	5.18E-39
UNC79	-1.272161397	3.448472696	-12.56921166	1.03E-31
ZNF204P	-1.272033598	2.686118817	-13.78286657	6.58E-37

CBLN1	-1.270607257	2.879426634	-12.35311836	8.18E-31
KCTD16	-1.269644029	1.408172308	-19.23897572	2.83E-62
EXTL1	-1.269301275	2.93707006	-14.91212575	6.28E-42
FADS6	-1.269282793	0.712722589	-20.39797187	6.75E-68
CEP170B	-1.266747835	3.992769062	-19.15238703	7.42E-62
PNMA8B	-1.266290165	3.77283441	-18.77357797	4.98E-60
PDE1A	-1.264905278	2.753978688	-12.83268115	8.03E-33
SLC1A6	-1.263779952	2.647613433	-13.59413081	4.37E-36
AC125616.1	-1.261384942	1.14266963	-16.53316811	2.20E-49
HSPA12A	-1.260677637	4.257111727	-16.17691083	1.00E-47
SOWAHB	-1.258451266	0.743996431	-18.7411184	7.13E-60
FSTL4	-1.258344615	1.575163619	-13.71415155	1.31E-36
SPRN	-1.255560822	3.390453811	-19.72572479	1.25E-64
PARM1	-1.255008794	3.425836377	-12.02544206	1.83E-29
AC134312.3	-1.25168268	0.778199748	-17.89554005	8.04E-56
FAM189A1	-1.250981798	2.324729397	-15.31494291	9.33E-44
AC092720.2	-1.250361599	1.468740756	-20.71719115	1.88E-69
MAST1	-1.249247435	4.51204666	-14.41779184	1.04E-39
CYP26B1	-1.24880519	1.971822135	-12.30610805	1.28E-30
GPR12	-1.248789707	1.407686472	-15.3874634	4.35E-44
DLG4	-1.245665674	6.222700467	-24.08433083	6.80E-86
LRRC73	-1.242163314	2.327341678	-20.64828789	4.08E-69
LRFN2	-1.241915672	1.636247748	-16.94956666	2.45E-51
CLSTN3	-1.241496911	5.498133917	-17.24120471	1.03E-52
SSTR1	-1.240714368	2.812018348	-9.635422495	2.78E-20
GPR26	-1.238542005	0.657147144	-18.97867423	5.12E-61
FGF13	-1.23643269	1.827661098	-19.00554227	3.80E-61
MCF2	-1.235305032	2.04716989	-16.04418382	4.14E-47
GRM5	-1.234604261	1.284957403	-14.39236459	1.34E-39
SGSM1	-1.232879565	3.117140948	-15.31472177	9.35E-44
PAK5	-1.232282941	2.533065545	-14.91318349	6.21E-42
PGBD5	-1.231266672	3.427939258	-19.76850021	7.73E-65
PRRT2	-1.230772955	4.815658494	-15.15502253	4.98E-43
CRTAC1	-1.228367432	5.444132045	-9.315434543	3.75E-19
PRRG3	-1.228354036	0.894940951	-18.46145412	1.58E-58
PRKAR2B	-1.228010712	4.01188342	-17.6881124	7.82E-55
GPRASP1	-1.227372839	3.966025455	-13.06995969	7.89E-34
IL34	-1.226069147	3.128866715	-13.57875364	5.10E-36
ATRNL1	-1.220956829	3.531163074	-14.40525504	1.18E-39
PLK2	-1.220718857	3.944462324	-13.73628383	1.05E-36
SLITRK4	-1.219290594	1.419259537	-15.25272196	1.79E-43
VIPR1	-1.218268709	1.916713016	-16.73894055	2.39E-50
AC012213.1	-1.218041773	1.853505751	-12.09116066	9.83E-30
GNG13	-1.215163035	1.177773081	-15.731493	1.15E-45
TMEM179	-1.213589939	4.70670429	-9.102275625	2.05E-18
RTBDN	-1.213552487	1.162983468	-20.18326349	7.48E-67
ANO3	-1.21309796	1.270780768	-15.10439195	8.46E-43
LHX5-AS1	-1.212447216	2.077480706	-7.024427929	6.99E-12
GPRIN1	-1.212331695	4.552207942	-14.40648343	1.16E-39
PHACTR1	-1.211849579	4.477107056	-16.61835707	8.78E-50
KCNF1	-1.209636339	4.757027528	-10.70767835	3.00E-24
RAPGEF4	-1.209215229	5.354432409	-11.97608379	2.91E-29
GALNT9	-1.2079333	4.047520415	-8.942377334	7.22E-18

CACNB1	-1.207810766	3.824853301	-19.46501143	2.28E-63
MAGEE1	-1.206424041	3.901039426	-16.79411258	1.32E-50
CHRM4	-1.203414358	2.182132352	-14.4337555	8.79E-40
VWC2L	-1.203318263	1.498718749	-12.34039016	9.24E-31
PRKCZ	-1.202185293	5.150731736	-15.1725662	4.15E-43
TMEM246	-1.202152824	4.074421758	-14.81116347	1.79E-41
SLC2A13	-1.201660836	3.738260622	-13.7336012	1.08E-36
CRHR1	-1.200584785	2.16860515	-18.23939166	1.83E-57
AC110491.1	-1.19982464	1.186131103	-15.0718899	1.19E-42
ATP6V1G2	-1.197784808	7.262867743	-13.66020463	2.26E-36
MT-TM	-1.197613583	2.533945578	-9.839015259	5.15E-21
AC021683.1	-1.197593364	1.070830083	-17.11874537	3.90E-52
AP001972.5	-1.197541834	5.072546706	-12.49046561	2.20E-31
LRRTM1	-1.197078118	2.907568763	-15.23673864	2.12E-43
C1QTNF4	-1.19638803	2.805216883	-12.92800692	3.17E-33
MMP24	-1.195933561	3.665352633	-12.14327885	6.01E-30
BCL11A	-1.195925789	1.590339185	-12.93235904	3.04E-33
AP3B2	-1.195473558	4.974217724	-17.65394562	1.14E-54
CLEC4GP1	-1.195091922	0.895154355	-16.60051365	1.06E-49
SMIM10L2A	-1.194342472	3.527537672	-18.02890192	1.86E-56
MCHR1	-1.194020599	2.446624833	-10.64705024	5.12E-24
PRRT1	-1.193752778	4.644577403	-19.55147857	8.71E-64
BEGAIN	-1.191782895	1.992148288	-14.94604126	4.41E-42
BASP1	-1.190271643	7.410141609	-14.91252655	6.25E-42
AC061961.1	-1.190228472	1.274039035	-13.8391443	3.73E-37
LINC02217	-1.18851518	0.863322533	-15.99488852	7.01E-47
LHX2	-1.188366532	4.244942527	-12.94127039	2.79E-33
PRKCE	-1.187961349	4.19804647	-17.11393061	4.11E-52
KCNQ2	-1.186500865	5.450873792	-13.4986295	1.13E-35
THY1	-1.185552049	7.078522615	-15.07176318	1.19E-42
CASKIN1	-1.185229643	4.009778626	-15.80655762	5.19E-46
ANK1	-1.184743829	2.380524539	-15.41552648	3.24E-44
KCNK9	-1.182693365	1.20790327	-13.80710437	5.15E-37
FLJ33534	-1.182135764	0.77838851	-18.01551382	2.15E-56
CRH	-1.182083284	0.84847468	-16.9266534	3.14E-51
PTPRN2	-1.181765472	5.871548958	-18.12870174	6.19E-57
C2CD4C	-1.181389572	3.059082639	-15.64445738	2.89E-45
PPP1R16B	-1.180888948	4.205490685	-11.64685882	6.24E-28
CORO6	-1.179845677	2.235778151	-11.44455779	4.02E-27
SUSD5	-1.178453491	3.559326421	-7.916372397	1.57E-14
KIF3C	-1.175394464	6.11437224	-17.3677107	2.60E-53
DOC2B	-1.175092298	2.69805543	-13.83631874	3.84E-37
LINGO1	-1.173846989	6.757588497	-12.5945599	8.07E-32
RPSAP69	-1.1723989	0.708363994	-19.86282898	2.69E-65
KIAA0319	-1.171882483	1.940533007	-12.33375476	9.84E-31
RAB26	-1.171614951	3.12884726	-16.30430585	2.57E-48
GLRA2	-1.169825011	1.030270921	-14.67064742	7.67E-41
AC004816.1	-1.169596212	1.903122595	-16.16699628	1.12E-47
CAMSAP3	-1.16915696	3.231971354	-13.03694511	1.09E-33
VWA7	-1.16895352	1.179599702	-17.4657078	8.91E-54
DLGAP2	-1.168793884	1.117206222	-16.9366192	2.82E-51
RTN1	-1.167655878	7.792923581	-12.48525998	2.31E-31
GDAP1L1	-1.167007203	5.260426406	-10.35211487	6.64E-23

CAP2	-1.165715265	4.297654547	-10.21755708	2.11E-22
KCNQ3	-1.164950006	2.804124407	-14.02978799	5.43E-38
UNC80	-1.164335788	3.46311112	-14.2818634	4.17E-39
SH3GL2	-1.164007078	6.123750535	-9.53136223	6.52E-20
LINC00643	-1.163644862	3.048666527	-9.476144465	1.02E-19
GRM3	-1.162822866	3.616539279	-9.874725747	3.82E-21
AC018358.1	-1.160825877	0.629301123	-18.85250913	2.07E-60
ZNF385B	-1.160484826	1.555191016	-13.62354634	3.26E-36
CCDC3	-1.16048172	3.562034127	-12.31677308	1.16E-30
LRRC7	-1.158565226	2.009093381	-15.17699907	3.96E-43
FBLL1	-1.157764615	4.606021416	-12.0541232	1.39E-29
SDR16C5	-1.156294707	0.722909097	-19.96784424	8.33E-66
KIF5C	-1.152424754	6.4897621	-15.46829266	1.86E-44
AL583859.2	-1.151447016	0.747151463	-16.17729631	1.00E-47
PANX2	-1.151355467	2.967845949	-14.15674372	1.49E-38
LAMP5	-1.151167824	4.415487274	-10.14685955	3.85E-22
MT-TL1	-1.150656273	3.969092513	-10.36664015	5.86E-23
PLPPR3	-1.147272099	2.945108629	-9.645445287	2.56E-20
MMP17	-1.146446401	3.726533113	-11.72984466	2.89E-28
AC005696.4	-1.146289938	3.761661498	-11.32399613	1.21E-26
PRKAG2-AS1	-1.145361596	3.065060845	-16.66743013	5.17E-50
FABP6	-1.144651934	1.787846292	-11.99318004	2.47E-29
AIFM3	-1.144425099	3.391696539	-8.433230077	3.55E-16
KALRN	-1.143931952	3.648247856	-17.77390223	3.06E-55
LINC02552	-1.143703492	1.186619147	-12.3790952	6.38E-31
ELOVL4	-1.143447071	3.248767119	-20.72514848	1.72E-69
TRHDE	-1.143146392	0.782841352	-18.86576173	1.79E-60
TPPP	-1.143113217	5.909236673	-10.91001385	5.01E-25
ACBD7	-1.141892123	4.25802913	-8.785492384	2.44E-17
AL354863.1	-1.140462358	1.487771588	-12.14104288	6.14E-30
GNAL	-1.140050471	3.52575923	-13.77511041	7.11E-37
TMEM63C	-1.139824573	3.393426122	-15.81206803	4.90E-46
CDH13	-1.136118255	3.623259923	-12.72057005	2.39E-32
MTUS2	-1.13568801	0.867907653	-20.28572979	2.37E-67
AC138649.1	-1.13499438	1.77130568	-12.95980725	2.32E-33
ZNF365	-1.134908987	4.03386537	-14.89497758	7.50E-42
MIAT	-1.130792584	3.657417725	-10.00266733	1.31E-21
EPB41L4B	-1.129940057	2.207601454	-13.61840912	3.43E-36
REEP1	-1.129831668	4.635704466	-15.2174982	2.59E-43
RNF144A-AS1	-1.128834397	0.91281083	-17.34845154	3.20E-53
ZDHHC8P1	-1.128287744	1.755952699	-9.96922481	1.73E-21
AF106564.1	-1.126328845	1.534467124	-17.63280319	1.43E-54
GABBR1	-1.12479135	8.007352416	-12.65886586	4.34E-32
SHANK3	-1.124572071	4.12118867	-17.7923294	2.50E-55
HLF	-1.12444162	4.355040544	-16.3744164	1.21E-48
EMX2OS	-1.124152739	2.851697983	-10.81055333	1.21E-24
DIRAS1	-1.12163868	5.348480873	-15.07091261	1.20E-42
SLC25A22	-1.121597093	5.536520362	-19.12613493	9.94E-62
KCNK12	-1.120815068	1.719321179	-13.63548057	2.89E-36
NEGR1	-1.120169801	2.922683445	-13.03485194	1.11E-33
HPRT1	-1.118490819	4.717318279	-16.14744782	1.38E-47
ACSL6	-1.118167157	4.854152585	-11.77626938	1.88E-28
CDKL5	-1.116055499	2.445666223	-14.69714314	5.83E-41

IQSEC1	-1.115734028	5.34559727	-16.48040491	3.88E-49
RASGRF2	-1.114550222	2.694348889	-13.47546964	1.43E-35
DRD1	-1.112497097	1.051456006	-15.11667898	7.44E-43
RAPGEFL1	-1.112431488	4.089939479	-16.59646358	1.11E-49
SH3GL3	-1.111779889	4.174991162	-10.29219628	1.11E-22
PDZD4	-1.111136948	7.295747726	-14.74040514	3.73E-41
CHRNA4	-1.110554398	3.14953943	-11.18239546	4.36E-26
KIAA1211L	-1.108816694	3.127563847	-12.3730391	6.76E-31
SYT12	-1.108671255	2.762645642	-14.51545058	3.80E-40
TGFBR3L	-1.107658524	1.18095828	-16.66266561	5.44E-50
ELAVL4	-1.107523614	4.358322585	-10.7637532	1.83E-24
GPR27	-1.106796991	4.030699903	-9.921326134	2.59E-21
ANKRD34C-AS	-1.106784541	0.612486461	-18.97687147	5.22E-61
SLIT1	-1.106589603	5.939027112	-8.757107585	3.04E-17
TMEM35A	-1.106192292	4.991794331	-15.10265877	8.61E-43
RIMKLA	-1.105550983	2.106413992	-17.90775424	7.03E-56
AC134312.1	-1.105482568	0.69489007	-19.07506422	1.75E-61
AC023301.1	-1.104681519	3.272093625	-11.24308989	2.52E-26
GPC5	-1.10461855	2.595776177	-9.218971147	8.12E-19
CDH8	-1.103216494	1.385666174	-13.87602982	2.57E-37
DLG2	-1.10199794	4.126362604	-13.07900537	7.22E-34
ZDHHC22	-1.10086193	6.332628975	-9.50373412	8.17E-20
AC026790.1	-1.100737675	1.042840583	-17.97887333	3.22E-56
SNPH	-1.099033894	4.345603357	-16.80638297	1.15E-50
PPL	-1.096528756	1.438787669	-10.24980243	1.60E-22
ATP2B1	-1.095281891	4.609412982	-13.1614288	3.21E-34
KCNB2	-1.092210422	0.684104061	-21.67273929	4.09E-74
YPEL4	-1.089069071	3.509663954	-13.75378059	8.81E-37
RAB11FIP4	-1.089064497	4.416145552	-14.34991174	2.08E-39
PNMA6A	-1.089040893	3.090574713	-13.30257675	7.96E-35
PPFIA3	-1.086880428	4.132306051	-18.29315474	1.01E-57
GFRA1	-1.085475996	3.557066423	-8.729748526	3.75E-17
ANKS1B	-1.084513971	4.726029047	-11.62248705	7.82E-28
SLC7A10	-1.083245922	1.485874093	-7.896647836	1.80E-14
GLRB	-1.082057157	4.657001042	-15.95779483	1.04E-46
SEMA6B	-1.081286615	5.399194269	-13.56716851	5.72E-36
STEAP2	-1.074175078	2.475793889	-14.12426561	2.08E-38
ARPP21	-1.073967933	5.173545629	-11.1814826	4.39E-26
SYT2	-1.072478888	0.863397671	-14.97343695	3.32E-42
CACNB3	-1.072127934	3.633218463	-14.27125539	4.64E-39
DLG3	-1.071918638	3.953134263	-18.76266982	5.62E-60
TMEM132B	-1.071323614	3.997310229	-10.57377436	9.71E-24
NXPH2	-1.07067091	0.956760025	-14.7237407	4.43E-41
GALNTL6	-1.069825013	0.956154529	-20.03076512	4.12E-66
CHRNA2	-1.06949527	0.683671958	-18.19161743	3.10E-57
MCTP1	-1.067219755	2.119282185	-13.68579444	1.74E-36
ISX-AS1	-1.066586856	1.120946022	-11.90423248	5.70E-29
C9orf24	-1.065728692	1.84111769	-8.442333183	3.32E-16
RASGRF1	-1.064803725	3.562939155	-8.587251935	1.11E-16
CAMKK2	-1.063780988	4.826986175	-16.78157259	1.51E-50
DCTN1-AS1	-1.063026078	0.928826317	-18.68208222	1.37E-59
DUSP2	-1.062881636	2.06601292	-13.73277467	1.09E-36
AP005901.3	-1.062790328	1.142953515	-13.16089862	3.23E-34

FBXW7	-1.062680156	4.110153142	-15.6902924	1.78E-45
SNORD113-3	-1.061340397	1.69799141	-10.72825744	2.51E-24
11-Mar	-1.060189756	0.954019839	-17.68942704	7.71E-55
LINC02440	-1.059189647	2.55349664	-9.568645217	4.81E-20
CAMK2N2	-1.058234538	4.544357567	-13.3132917	7.16E-35
PSD3	-1.057025036	4.42097731	-14.32223316	2.76E-39
BCYRN1	-1.056547224	2.068718869	-9.838477833	5.17E-21
INSM2	-1.055954665	1.035456562	-13.46559019	1.58E-35
DGKK	-1.055451292	1.199782872	-11.03941817	1.58E-25
DUSP8	-1.055365065	4.041278395	-16.59597675	1.12E-49
KNDC1	-1.055167618	4.087672436	-11.55956112	1.40E-27
ELMOD1	-1.054849943	4.250673646	-11.03989242	1.57E-25
TSPOAP1	-1.052242232	4.91510513	-14.02756735	5.55E-38
YWHAH	-1.052238082	7.889489808	-14.11938642	2.18E-38
GABBR2	-1.05163994	5.333262844	-7.953811142	1.20E-14
TCERG1L	-1.051154163	0.761584782	-18.36881081	4.38E-58
CNKSR2	-1.050727705	3.667683166	-14.01453637	6.33E-38
CDS1	-1.05067852	2.319139031	-10.26999838	1.35E-22
EGR4	-1.05054539	0.813509189	-13.04554071	1.00E-33
ACVR1C	-1.050065167	1.067202623	-18.93808626	8.03E-61
SPHKAP	-1.049635604	3.062590285	-7.607245662	1.38E-13
ANO5	-1.048757927	2.46100585	-12.65339785	4.57E-32
MEG3	-1.048204588	4.452271463	-8.449914728	3.14E-16
COX7A1	-1.047831148	4.089098588	-9.812489067	6.42E-21
INPP5F	-1.04660161	4.83096923	-15.50757904	1.23E-44
STXBP5	-1.045972956	2.670525984	-17.64389846	1.27E-54
CACNA2D1	-1.045545253	3.577062307	-11.95964159	3.39E-29
HMGCLL1	-1.045502149	2.4514388	-10.36136167	6.13E-23
TMEM233	-1.045028957	1.13724611	-12.96935282	2.12E-33
AL354798.1	-1.043901218	2.231889908	-13.69343779	1.62E-36
ATP1B1	-1.043414812	7.118346065	-11.08748806	1.02E-25
KIAA1549L	-1.043368188	3.727488803	-13.25233401	1.31E-34
ARX	-1.042709594	1.79556515	-10.79837555	1.35E-24
SAMD12	-1.042668131	1.699464351	-16.26132445	4.07E-48
ANO4	-1.041725798	2.166757473	-13.65619522	2.35E-36
THRB	-1.041286937	3.228683132	-14.18991099	1.06E-38
AC011995.2	-1.040016075	0.711184939	-16.27297076	3.59E-48
SLC13A5	-1.039784176	1.051087638	-11.45791881	3.55E-27
FAIM2	-1.037893301	7.600516242	-12.87560267	5.29E-33
CDHR1	-1.037808377	4.089450887	-9.575698655	4.54E-20
ADRB1	-1.03758578	2.067949893	-15.38487769	4.47E-44
RHBDL1	-1.036903435	3.355010616	-13.35140909	4.91E-35
LY6E-DT	-1.036258045	1.347727819	-20.62037212	5.58E-69
DLGAP1	-1.035471146	5.074500145	-10.74729086	2.12E-24
ENO2	-1.035289753	7.941323114	-17.04045294	9.13E-52
BRSK2	-1.035256224	4.879632275	-13.93762502	1.38E-37
CAMK2N1	-1.034873036	7.369279882	-12.91342887	3.66E-33
CDKN2D	-1.034467038	4.826146521	-17.4353105	1.24E-53
GUCY1A1	-1.034053272	3.336534132	-10.50827199	1.72E-23
B4GALT6	-1.033879968	3.171295949	-15.38593185	4.42E-44
LGI3	-1.033860639	4.58007921	-8.355341652	6.36E-16
CALB1	-1.033663023	1.744665976	-9.882417771	3.58E-21
AC124312.2	-1.03359085	3.800946059	-9.419294422	1.62E-19

DGKZ	-1.03261556	5.26800082	-17.11531352	4.05E-52
LINC01551	-1.03254393	2.149546326	-11.02264663	1.83E-25
VSTM2A-OT1	-1.032179193	0.873857707	-18.33916114	6.08E-58
SEZ6	-1.031575952	5.497694457	-8.336437321	7.32E-16
KCNQ5	-1.031410844	2.948262742	-10.72827336	2.51E-24
REPS2	-1.030658702	3.856805332	-11.12766279	7.14E-26
CLDN10	-1.027900425	2.716084942	-8.289605059	1.04E-15
IQSEC2	-1.027353488	4.166570356	-13.09516086	6.16E-34
SEMA4A	-1.027224425	3.706782566	-13.09554388	6.14E-34
LRRTM4	-1.027000582	3.589097305	-10.53235185	1.39E-23
CACNB4	-1.025734714	3.125154619	-16.8795263	5.23E-51
TENM3	-1.025185644	1.46965665	-10.5966825	7.95E-24
AC107398.3	-1.024949902	3.549976051	-9.772692762	8.94E-21
LONRF2	-1.024598491	3.968843045	-14.98378703	2.98E-42
AC010857.1	-1.024513852	0.683360418	-19.46793731	2.21E-63
MAST3	-1.02287023	4.524883829	-13.93293452	1.45E-37
GRIP2	-1.022721199	1.231976044	-16.93673199	2.81E-51
KCNMA1	-1.020823975	3.645408773	-13.85805079	3.08E-37
HOOK1	-1.020504375	0.914909435	-15.85468304	3.12E-46
ERC2	-1.019785249	2.532277905	-13.76839094	7.61E-37
SRCIN1	-1.018345457	4.172460528	-9.985075977	1.51E-21
DGCR5	-1.018228437	3.256446281	-14.06743295	3.70E-38
FEZF2	-1.015559324	1.932989549	-11.26165415	2.13E-26
WASF1	-1.01546083	5.850756246	-14.03877063	4.95E-38
C1QL2	-1.014976084	2.183867707	-9.809051877	6.61E-21
GPR68	-1.013530186	1.105754688	-16.41451165	7.87E-49
MIR124-2	-1.012107963	1.268755714	-12.42309333	4.19E-31
TSPYL2	-1.011571236	6.105023655	-13.49242065	1.21E-35
GPRASP2	-1.011530919	4.338359779	-16.71563617	3.07E-50
LINC01202	-1.010861857	0.514027694	-15.77121139	7.56E-46
SLIT2	-1.009695531	1.812276958	-10.60598485	7.33E-24
LINC01476	-1.008921621	0.50650024	-15.7250087	1.23E-45
EIF4E1B	-1.006809562	0.62226963	-21.54744972	1.67E-73
APBA1	-1.003275976	4.181734462	-17.33430871	3.74E-53
NRXN1	-1.00212605	5.360564313	-10.99546327	2.34E-25
CNTNAP5	-1.000090292	1.525456054	-11.66262621	5.40E-28
FLNA	1.000506295	6.520003521	12.12574161	7.09E-30
SQOR	1.000509043	2.88719903	15.38828383	4.31E-44
CA3	1.002839213	1.559197458	8.158243767	2.72E-15
PLAUR	1.0037403	3.048895835	11.05498432	1.37E-25
LHFPL2	1.004397013	4.393758062	14.46510821	6.37E-40
KIAA0040	1.006478537	2.687549976	9.812042965	6.45E-21
HLA-B	1.007120005	9.432087222	11.8323648	1.12E-28
TREML1	1.007239395	1.935066311	14.63649406	1.09E-40
NFATC1	1.007555413	2.600840954	14.0953348	2.79E-38
GBX2	1.007670265	2.473981386	8.437612457	3.44E-16
HLA-DQB2	1.008749694	1.545237931	8.690757341	5.05E-17
HPSE	1.010741114	2.14332427	14.10093081	2.63E-38
HS3ST1	1.012042227	2.302165304	10.11708228	4.96E-22
FGL2	1.015489866	2.979476712	10.73723314	2.31E-24
PLVAP	1.015849821	3.633427421	11.3242874	1.20E-26
ICAM1	1.018435704	2.926642707	10.30762115	9.74E-23
AC018755.4	1.020360824	1.984752178	12.2240907	2.79E-30

SERPINE1	1.020811487	3.37945918	6.243193474	9.10E-10
ASAP3	1.021715596	5.03403257	14.88098281	8.67E-42
SLAMF8	1.024074506	1.530781566	12.61810192	6.43E-32
BST2	1.024757	6.259368855	10.78566728	1.51E-24
PIK3AP1	1.02476736	3.069568933	14.35502835	1.97E-39
MFNG	1.025594141	4.149016251	14.85759398	1.11E-41
GAL3ST4	1.02660503	4.556878924	16.11658191	1.91E-47
WDFY4	1.027646021	1.929513563	16.52324791	2.45E-49
RAB13	1.030858629	6.342116251	19.88936569	2.00E-65
OAS1	1.032619387	3.656409457	9.597528482	3.80E-20
FCGRT	1.032764132	6.458189302	20.37129633	9.10E-68
STEAP3	1.032912576	2.906025461	8.488354191	2.35E-16
FGD2	1.035022195	2.589504774	14.94103422	4.65E-42
PLSCR1	1.035062401	4.286756442	11.82689057	1.17E-28
EMP3	1.035636033	4.13356072	6.24556166	8.97E-10
P2RY13	1.036192024	3.543277482	11.61767107	8.18E-28
CYR61	1.037283466	4.9808227	6.949214944	1.14E-11
ARHGAP30	1.038272886	3.126364553	16.7220827	2.87E-50
NPC2	1.039117401	7.133090623	17.92890051	5.57E-56
IQGAP2	1.041869727	2.264969153	9.386505252	2.12E-19
HAMP	1.042495575	1.796724187	9.310479236	3.90E-19
FCGR1B	1.042661316	2.366905618	13.34610266	5.17E-35
KCNQ1	1.043268896	2.900681646	17.66285965	1.03E-54
ADAMTS15	1.043402636	2.157885838	9.473112004	1.05E-19
CD300C	1.043873704	1.832632056	17.42676348	1.36E-53
FUOM	1.045024749	3.114538287	15.44887778	2.28E-44
CTSZ	1.045197476	6.548691743	14.89528014	7.48E-42
PDE8A	1.046824419	4.217209394	12.47909331	2.45E-31
AEBP1	1.047994497	5.462522359	5.874215531	7.72E-09
TUBB6	1.048391684	4.186072411	10.19599751	2.53E-22
PIFO	1.049619693	2.849500277	10.33680432	7.58E-23
F2RL1	1.053291583	2.023982901	10.16118577	3.41E-22
RASAL3	1.05404182	2.25365265	16.94762899	2.50E-51
MSTN	1.055183433	3.280053625	6.637183783	8.27E-11
STAT5A	1.058612146	3.311688697	16.3871259	1.06E-48
STK19B	1.059392423	1.918191037	9.291605018	4.54E-19
DHRS3	1.060098439	6.023494943	12.82598732	8.57E-33
FOXD3-AS1	1.062111179	1.699387552	8.441913844	3.33E-16
HAS2	1.063230493	2.160946912	11.42756275	4.69E-27
SCIMP	1.064973111	1.994524566	15.73508092	1.11E-45
IKZF1	1.065085912	2.299042283	15.45953906	2.04E-44
TMC8	1.067200238	2.448625399	17.96909238	3.58E-56
LILRA2	1.070681085	1.857900414	16.81904996	1.01E-50
BIN2	1.072955461	3.068193709	15.21275948	2.72E-43
CIITA	1.075394215	1.661757625	12.83804003	7.62E-33
COL4A1	1.07610422	4.077163978	7.135188417	3.38E-12
AOAH	1.076393967	3.44870511	15.05587045	1.40E-42
HOTAIRM1	1.076538397	1.665718828	8.408591498	4.27E-16
SELPLG	1.07655374	5.391117081	11.4357271	4.35E-27
ARHGAP4	1.076671644	4.100465613	16.53266972	2.21E-49
FBXO32	1.076998027	4.72943462	11.1279339	7.12E-26
ABCC3	1.077341474	1.290323932	8.048309686	6.06E-15
APLNR	1.077471143	5.933343388	6.88507008	1.72E-11

COL1A1	1.080304619	2.802278719	6.739308011	4.36E-11
MSN	1.0821136	6.349460168	10.90071309	5.44E-25
CSTA	1.085170835	1.381521675	11.90786945	5.51E-29
APOBR	1.085894247	2.268178062	17.09853973	4.86E-52
IL13RA1	1.087089733	4.98937727	14.58194295	1.91E-40
GPR65	1.087491345	1.245024325	15.05621716	1.40E-42
SP100	1.087594206	3.235102139	14.86210921	1.06E-41
SLA	1.08849059	3.999760348	12.10013056	9.03E-30
BCL2A1	1.090336303	1.889453971	10.77021248	1.73E-24
STXBP2	1.090672539	3.351082199	17.15090521	2.75E-52
AL035446.1	1.091846306	1.456215862	10.02614542	1.07E-21
P3H2	1.092300317	2.408813449	12.64492104	4.96E-32
S100A6	1.093463861	8.959470584	11.67217607	4.94E-28
LYL1	1.09708368	3.452942826	16.12372468	1.77E-47
AGTRAP	1.097243207	5.616435189	18.09467818	9.00E-57
CPVL	1.098118863	4.442078015	11.21962763	3.11E-26
CARD16	1.098293453	2.088086363	12.95168951	2.52E-33
AC092131.1	1.098315425	1.368964491	11.74074841	2.62E-28
RNASET2	1.099713844	5.659224679	15.58262011	5.56E-45
PLCB2	1.101049591	3.81060429	14.45708841	6.92E-40
AC138207.5	1.101344935	2.745406723	17.71500675	5.83E-55
LPAR6	1.102748925	3.779481582	15.97041036	9.10E-47
FCGR1CP	1.103869428	1.987846704	14.56506748	2.28E-40
CLEC7A	1.103894795	2.136779621	13.95854828	1.12E-37
SIRPB2	1.105437729	2.019865178	15.82664986	4.20E-46
HERC5	1.107344269	2.926123287	12.64438406	4.99E-32
DOCK8	1.108656895	2.735205895	14.49735279	4.57E-40
SH3TC1	1.11579133	3.097833196	15.05880138	1.36E-42
PLXDC2	1.115911825	3.795315309	14.14534074	1.68E-38
RAB32	1.119466118	3.596332113	15.29714891	1.12E-43
WAS	1.120070536	4.205055888	17.44174184	1.16E-53
FOLR2	1.125469565	5.261732269	10.16406001	3.33E-22
TNFRSF1A	1.125798377	5.942120259	14.59110223	1.74E-40
SUSD3	1.12708796	3.185282271	12.36448192	7.34E-31
CSF1	1.127784442	5.5998643	15.89072729	2.12E-46
AL355974.2	1.127811553	4.058003282	8.359864661	6.15E-16
TMEM176B	1.128364594	5.599541383	10.32682273	8.26E-23
AL355974.3	1.130811386	2.296037601	9.322878074	3.53E-19
CHI3L1	1.136067365	5.030129177	4.113862039	4.54E-05
CEBPD	1.136681756	4.581305141	11.14847085	5.92E-26
HK2	1.139192925	3.531368457	13.83200065	4.01E-37
SLC16A3	1.139206902	2.992756925	15.48319613	1.59E-44
FLNC	1.140331573	3.216959064	8.388827407	4.95E-16
TLR1	1.14149157	2.391386989	14.69906547	5.72E-41
IRX1	1.14170534	2.880686767	7.380021253	6.56E-13
EFEMP1	1.142519542	6.38453008	9.669674973	2.10E-20
IRF5	1.142548277	3.454649507	17.14250805	3.01E-52
PRAM1	1.1517489	2.684442564	14.71485155	4.85E-41
PYGL	1.152897207	4.120850456	13.71538646	1.30E-36
CXCR4	1.153236856	4.854990823	12.79687851	1.14E-32
TGFB1	1.154714342	5.201995402	18.18520441	3.32E-57
LST1	1.155535305	4.025294582	16.13018962	1.65E-47
AL161785.1	1.156460908	2.260222573	17.53987149	3.96E-54

ADAP2	1.156888305	3.615582246	19.31443846	1.22E-62
APOBEC3C	1.160424551	3.30165789	14.22707186	7.29E-39
SLC14A1	1.161975416	4.366503128	5.870165271	7.90E-09
LPCAT2	1.162067117	3.191787482	13.8418838	3.63E-37
ADAM28	1.165553971	2.617122953	15.96941887	9.19E-47
MRC2	1.167073198	4.500094907	12.03448557	1.68E-29
SFRP4	1.168196684	2.841209591	9.207389321	8.91E-19
FOXJ1	1.168535946	3.133627834	9.177774728	1.13E-18
GPR183	1.170105999	2.844058232	11.96907335	3.10E-29
ITGAM	1.171285079	3.093479341	15.1140878	7.65E-43
CXCL10	1.173139383	1.639147384	8.292251568	1.02E-15
RARRES3	1.174883225	6.164528333	13.60964581	3.74E-36
AR	1.17604069	2.232678176	12.66031243	4.28E-32
LEFTY2	1.176216189	1.477637181	9.033919009	3.52E-18
INPP5D	1.176970342	4.204871534	17.11620452	4.01E-52
FXYD5	1.178237086	4.737559845	16.17785683	9.94E-48
ITGAL	1.181316005	2.095452431	15.63183066	3.31E-45
LYZ	1.189282655	2.9563608	8.522008424	1.82E-16
SRPX2	1.19006458	2.077613356	10.6473751	5.10E-24
CMTM7	1.192213298	2.806873121	18.51806671	8.43E-59
RBM47	1.194744226	1.969997964	17.37386234	2.43E-53
PLD4	1.196888639	3.20796174	13.19129435	2.39E-34
CFI	1.198845946	3.660071255	9.831001483	5.51E-21
WWTR1	1.199058231	4.766994522	12.26277393	1.93E-30
RUNX1	1.202168341	2.241155629	13.23610398	1.54E-34
TCIM	1.202828289	3.721272382	10.22119936	2.04E-22
C2	1.204212916	2.595486158	14.37442849	1.62E-39
CSF3R	1.204300267	2.98340432	16.43177862	6.54E-49
EBI3	1.205664699	3.533861876	16.56178637	1.61E-49
FBP1	1.206078294	2.494259896	17.21627935	1.35E-52
MNDA	1.207804584	3.617728309	14.59582941	1.66E-40
LY96	1.208462722	3.564072081	14.67699757	7.18E-41
S1PR3	1.208837971	3.183480485	12.13685633	6.39E-30
LINC01094	1.209063294	4.832424127	11.82461832	1.20E-28
TNFRSF1B	1.212716314	4.468528079	18.445556	1.88E-58
CD4	1.21389513	5.170434301	17.68691444	7.93E-55
IRF8	1.213982302	3.723894517	14.74033391	3.73E-41
TGIF1	1.214627079	3.660872675	14.87559173	9.17E-42
CSRP2	1.214936464	4.939022085	11.74562431	2.50E-28
LRRC25	1.215550646	2.395679164	16.8718348	5.68E-51
HPGDS	1.215553107	2.968193815	15.31056458	9.76E-44
GNG5	1.22177468	6.525972581	17.48420544	7.28E-54
SIGLEC14	1.223990526	2.314947108	13.35831482	4.58E-35
S100A4	1.224222237	3.377102794	9.968708555	1.74E-21
GNG12	1.228121673	4.417743586	10.61084669	7.02E-24
CTSC	1.228668851	3.548763338	15.9661491	9.52E-47
PCED1B-AS1	1.229934589	2.544468443	17.47149581	8.36E-54
GPSM3	1.230453605	4.770461949	19.12921646	9.60E-62
PIK3R5	1.230835434	2.628351821	18.6585925	1.78E-59
CLIC1	1.232101455	5.835466645	13.80452878	5.29E-37
BTK	1.234641988	2.924297717	18.48198688	1.26E-58
UNC93B1	1.235224778	4.036111356	16.81155785	1.09E-50
COL8A2	1.236524402	2.258602382	14.2483956	5.86E-39

CYTH4	1.238079314	3.348386673	16.85285344	6.98E-51
ARHGDIB	1.238348604	6.813572068	19.68441548	1.98E-64
BLNK	1.240001972	3.079619658	14.39106786	1.36E-39
DEF6	1.240923565	3.344059078	17.65398298	1.14E-54
TPTEP1	1.241023546	3.355507988	5.570213875	4.14E-08
RPS6KA1	1.242203893	4.080601844	17.50411119	5.86E-54
THEMIS2	1.243703148	3.9147847	15.96642513	9.49E-47
TGFBI	1.244076083	3.75304951	9.472806898	1.05E-19
TSPO	1.244310311	6.023401097	15.54690201	8.11E-45
CEBPA	1.245042089	3.611442893	15.61638596	3.89E-45
PTGS1	1.247436544	3.045709939	14.4695212	6.09E-40
OLFML2B	1.249492815	3.311336643	12.30695407	1.27E-30
VAV1	1.253570463	3.08246241	17.8761657	9.95E-56
LINC02381	1.253674298	3.451432244	12.77781115	1.37E-32
ARPC1B	1.255214508	5.905097709	19.4741281	2.06E-63
HCST	1.2588517	3.328975357	15.33121751	7.86E-44
SLC15A3	1.263280468	3.39177194	19.38710714	5.44E-63
ST14	1.264793894	2.115242952	13.8804277	2.46E-37
SULF1	1.264977449	3.514764875	8.77442873	2.66E-17
CD84	1.272889593	2.529713089	16.2453012	4.83E-48
IGSF6	1.277257991	3.317847684	17.91058126	6.82E-56
LCP1	1.279732312	4.255331923	16.49747305	3.23E-49
DOCK2	1.280036469	3.021578814	16.9754999	1.85E-51
MILR1	1.281137411	2.794919786	17.79231238	2.50E-55
TLR7	1.285517883	2.353247037	15.89917623	1.94E-46
RAC2	1.285565182	3.108047608	16.49237097	3.41E-49
LINC01736	1.290813471	4.131340653	9.96859435	1.74E-21
HLA-DQA2	1.291632236	2.251945218	7.956194791	1.18E-14
GYPC	1.29233765	4.2657671	17.31916133	4.41E-53
SLC37A2	1.292362465	2.56051653	19.20950054	3.93E-62
FYB1	1.295287915	3.433890127	14.93765306	4.81E-42
NCF4	1.295595993	3.274586746	19.3516125	8.08E-63
CSF2RA	1.296766767	4.069287228	15.26103209	1.64E-43
C1orf162	1.297310669	3.588760021	17.052915	7.98E-52
ANKRD22	1.298688394	2.257215816	10.88376648	6.33E-25
C4A	1.304904864	3.142711988	12.3516091	8.30E-31
PTPRC	1.305354237	3.095091038	14.42004709	1.01E-39
ITGAX	1.306467077	3.693085038	15.47656451	1.70E-44
CYTL1	1.307932415	3.242405733	14.56333541	2.32E-40
LTF	1.309501715	1.754337168	6.371174551	4.23E-10
IL10RA	1.311972089	3.007229567	17.72044996	5.49E-55
TNFAIP8L2	1.315170466	3.529413051	18.83260365	2.59E-60
SIGLEC10	1.316178326	3.933295006	16.25959024	4.15E-48
METTL7B	1.316731732	3.689319788	7.886503282	1.94E-14
AP000924.1	1.317214565	1.541888932	13.17724975	2.75E-34
OLFML3	1.318417218	5.014092411	16.18636961	9.08E-48
RGS10	1.321491662	5.98428906	16.30768594	2.48E-48
GEM	1.32505235	4.094377231	12.18661776	3.99E-30
SLC1A5	1.326812442	3.581417323	17.21981781	1.30E-52
STAB1	1.327071684	4.329068784	15.75808849	8.69E-46
NCKAP1L	1.328972166	3.071678211	18.48568072	1.21E-58
HLA-DQB1	1.330317259	3.471875692	9.632937044	2.84E-20
HMOX1	1.331183162	5.468872311	16.29891009	2.72E-48

SIGLEC8	1.331513417	4.04470585	13.60127532	4.07E-36
VAMP8	1.335889383	5.252726627	19.67064182	2.30E-64
PYCARD	1.336027252	3.638484295	18.81936281	3.00E-60
GLIS3	1.337195941	3.427846709	14.77734172	2.54E-41
FERMT3	1.338729791	3.97073167	19.89684975	1.84E-65
RHBDF2	1.338914131	4.277474867	16.90643478	3.91E-51
SIGLEC9	1.339075554	2.317791594	18.84920562	2.15E-60
LINC01088	1.34086238	5.908698774	7.421744253	4.94E-13
SYK	1.341796153	3.402980162	17.74556701	4.17E-55
PLEK	1.342396224	3.513269927	16.39097944	1.01E-48
PTPN6	1.345887657	4.146324108	19.49351918	1.66E-63
PARVG	1.346885232	2.761412528	20.27526935	2.67E-67
EVI2B	1.348232062	3.774105401	15.80202	5.45E-46
IFI44L	1.349304064	4.257805437	11.02738794	1.76E-25
MYO1F	1.349577663	3.694717136	19.56002359	7.92E-64
ALOX5	1.350152528	3.413432484	15.7481875	9.65E-46
SLC7A7	1.350790392	3.37993168	20.79542121	7.82E-70
GBP3	1.350963465	3.29824356	11.61578343	8.32E-28
ANXA2	1.353039665	4.975883603	12.41006059	4.75E-31
TBXAS1	1.359336105	4.358185306	18.73615181	7.54E-60
GPR34	1.361505117	5.128777411	14.03997509	4.89E-38
C5AR1	1.362454249	3.050683318	14.13503432	1.86E-38
HCK	1.365145444	4.07424105	18.98791062	4.62E-61
PLAU	1.367380476	2.542666664	12.17634177	4.39E-30
LPAR5	1.369723679	3.527691991	16.32897905	1.97E-48
CD300A	1.373104804	3.705345026	19.086666	1.54E-61
S100A3	1.373705725	1.900264837	11.44658319	3.94E-27
LAT2	1.379069283	4.135000083	18.13788529	5.60E-57
CTSH	1.380024371	6.201074359	11.38297017	7.05E-27
FPR3	1.381164603	1.952098017	14.28643288	3.98E-39
LILRB1	1.384342777	2.724821859	18.51582255	8.64E-59
RNASE6	1.389097748	4.472434841	17.11546641	4.04E-52
CSF1R	1.39089261	6.503066711	14.25850929	5.29E-39
CCR1	1.391504082	3.151860744	16.38708082	1.06E-48
RPE65	1.392183372	3.136566264	10.6000314	7.72E-24
SLC2A5	1.392268156	4.54794568	14.41045394	1.12E-39
TNC	1.401128476	5.756055022	11.53197572	1.80E-27
SAMSN1	1.40274331	3.436712798	16.67489764	4.77E-50
CASP1	1.409918418	3.279860575	18.38609267	3.62E-58
C1R	1.410268346	5.224702062	11.82053684	1.25E-28
MMP14	1.413833639	5.019878615	14.09698032	2.74E-38
CX3CR1	1.414181063	5.250273303	10.380642	5.19E-23
CAPG	1.416756793	5.787594762	13.50120675	1.11E-35
LGALS9	1.421937277	4.714280027	19.50536164	1.46E-63
CD86	1.424401716	3.326157592	18.10223043	8.29E-57
CRNDE	1.432597131	2.712418685	9.837264425	5.23E-21
GPNMB	1.433589957	4.243070691	9.798078258	7.24E-21
ECM2	1.435250462	3.4328612	12.7109109	2.62E-32
AIF1	1.43922324	5.832086322	17.57951246	2.57E-54
NFIA-AS2	1.450301525	3.559212647	13.62595242	3.18E-36
FCGR1A	1.453613646	3.472487624	16.59664961	1.11E-49
ABI3	1.454065681	3.87428475	19.5097938	1.39E-63
TMEM119	1.455656529	4.497476355	13.41091252	2.72E-35

IL18	1.458859267	3.596365589	17.65532084	1.12E-54
APBB1IP	1.459251667	4.434515957	15.94481818	1.19E-46
HLA-DRB6	1.468220784	3.456757739	10.56350111	1.06E-23
PTAFR	1.472529765	3.293916664	17.9682995	3.62E-56
CD53	1.478388026	5.640582426	17.8811948	9.41E-56
TLR2	1.478773758	2.955283846	15.84763357	3.36E-46
C3AR1	1.478851384	4.680495823	15.67147903	2.17E-45
HCLS1	1.479004491	5.239098055	17.79096055	2.53E-55
SASH3	1.479226446	3.616953481	19.39255542	5.12E-63
GFAP	1.479315109	13.48316258	11.54162639	1.65E-27
CTSS	1.479923942	4.147932164	18.74706809	6.68E-60
RGS1	1.484864737	4.990780852	9.957634803	1.91E-21
CYBA	1.48491818	5.675030243	20.52070779	1.71E-68
LY86	1.488038811	4.642288351	18.19388631	3.02E-57
C4B	1.499810769	3.264752899	13.60520945	3.91E-36
CD37	1.502639557	3.778194332	18.58130245	4.19E-59
APOL4	1.504971142	2.253576705	11.8302632	1.14E-28
MS4A4A	1.505520079	3.626384499	16.27259932	3.61E-48
LAIR1	1.508104284	3.407876278	18.96392018	6.03E-61
RNASE2	1.51861579	2.260654706	14.01210431	6.49E-38
TMIGD3	1.518932215	5.326043476	17.0826906	5.77E-52
PI16	1.525112779	4.086017805	9.856108518	4.47E-21
HAVCR2	1.527160189	4.234446598	18.83372568	2.56E-60
HLA-DQA1	1.530918404	2.841974546	11.06102544	1.30E-25
CP	1.534959553	2.809676061	11.03907045	1.58E-25
NMB	1.544681954	7.457057702	11.15017423	5.83E-26
CD99	1.545214927	8.92967472	14.74676087	3.49E-41
GBP1	1.551706079	3.93311073	13.26939845	1.11E-34
OLR1	1.551811593	4.441462235	12.80384417	1.06E-32
CCL2	1.554899001	4.783118348	10.2906087	1.13E-22
ID3	1.559599708	8.304591864	14.95092922	4.19E-42
S100A10	1.561489641	6.503844508	14.03557613	5.12E-38
SPI1	1.56637455	5.24319969	20.82517799	5.60E-70
SERPINA1	1.566622555	3.284166124	14.62270895	1.26E-40
SLC11A1	1.571190279	2.779957703	14.45841686	6.83E-40
CD163	1.571693741	3.004524404	10.08283747	6.63E-22
TNFRSF12A	1.574129706	3.521193056	11.91651103	5.08E-29
MS4A7	1.57436835	4.251990153	18.67085549	1.55E-59
HLA-DMA	1.575036598	5.339001397	17.5360216	4.13E-54
HLA-DPA1	1.579279891	5.244768134	12.27954778	1.65E-30
TYMP	1.584371363	3.389461969	13.99798465	7.49E-38
LAPTM5	1.608658816	7.483057551	20.15042701	1.08E-66
ANXA1	1.618765529	5.066659445	10.04778509	8.92E-22
EMP1	1.624992833	4.982650625	13.59240292	4.45E-36
HLA-DMB	1.626577791	3.662053405	18.98471736	4.78E-61
ALOX5AP	1.627939899	4.69805485	14.35347977	2.00E-39
HLA-DOA	1.628790119	3.327597564	13.78319565	6.56E-37
UCP2	1.633790898	5.146054998	21.17537077	1.10E-71
HLA-DRB5	1.640618374	5.644423727	11.49319921	2.57E-27
TYROBP	1.645422558	7.164607564	21.28689796	3.13E-72
PLEKHA4	1.648941403	4.194034411	14.43197978	8.95E-40
S100A11	1.650049308	6.71217793	17.91172466	6.73E-56
C1QA	1.651767999	7.593391721	18.37830837	3.95E-58

SPOCD1	1.651938713	2.325280415	10.79657508	1.37E-24
LILRB4	1.65590459	4.066927151	20.25472923	3.36E-67
MSR1	1.655923771	3.12505973	16.63232538	7.55E-50
SCIN	1.658495734	2.469433424	16.12444094	1.76E-47
HLA-DPB1	1.661203815	6.496605075	13.16485256	3.10E-34
FPR1	1.669531545	3.948402168	14.72484121	4.38E-41
FCGR2A	1.673990331	4.107665012	19.42672572	3.50E-63
C1QC	1.68837093	8.04924194	19.47118307	2.13E-63
ITGB4	1.692980579	4.875766758	13.83839084	3.76E-37
CYBB	1.693876865	4.571516477	17.80663133	2.13E-55
ITGB2	1.69420379	5.033581696	19.67537927	2.19E-64
HLA-DRB1	1.71968406	7.298146893	13.31234632	7.23E-35
FCER1G	1.741920058	6.369352957	21.67283842	4.08E-74
C1QB	1.757369715	8.053918015	18.86454218	1.82E-60
CD74	1.759174368	9.570773397	15.48742693	1.52E-44
CD14	1.76378843	6.023441918	17.95053792	4.39E-56
CD44	1.766583458	6.392529872	15.17946325	3.86E-43
VSIG4	1.772558188	5.579294441	16.7051096	3.44E-50
SPP1	1.818610504	9.028893187	12.98481624	1.82E-33
HLA-DRA	1.822854047	8.197158495	13.7421072	9.91E-37
PDPN	1.8286456	3.47987262	11.29055201	1.64E-26
VCAM1	1.835428312	4.193021776	13.09883935	5.94E-34
GBP2	1.836172257	4.084085799	15.45968819	2.03E-44
NAPSB	1.840905869	3.840771678	12.73301214	2.12E-32
VIM	1.851440448	8.912432732	14.97831436	3.15E-42
FCGR3A	1.867061443	5.130066296	15.71974456	1.30E-45
MS4A6A	1.874277516	4.18793544	16.32080816	2.15E-48
TREM2	1.895930172	6.313840286	19.69490622	1.76E-64
C3	1.939445596	7.297488925	15.98681322	7.64E-47
CHI3L2	2.055696352	4.027676433	12.18226305	4.15E-30
FCGBP	2.107678341	3.332905117	15.01883452	2.07E-42

adj.P.Val	B
3.03E-62	136.6434771
3.39E-62	136.5264702
5.99E-52	112.1153526
1.31E-62	137.5122477
2.72E-57	124.783217
1.29E-70	156.9372062
2.66E-63	139.1730194
7.76E-71	157.4610228
5.45E-66	145.6335729
2.32E-69	153.7992477
5.29E-72	160.2813963
5.35E-65	143.2270483
1.47E-60	132.5848086
7.13E-73	162.5196402
1.96E-77	173.5720547
8.99E-72	159.7335147
8.96E-61	133.0993921
1.20E-56	123.2520281
2.30E-72	161.2344974
5.67E-55	119.301563
4.68E-58	126.6119893
1.28E-62	137.5460371
9.29E-60	130.692377
8.51E-69	152.4510208
1.30E-78	176.5880629
9.01E-67	147.5567366
3.85E-84	190.8304623
1.91E-78	176.1471631
3.11E-72	160.8900861
5.81E-71	157.8019162
3.53E-41	86.77673691
6.29E-66	145.4821261
1.10E-76	171.6113372
9.65E-77	171.8579221
8.28E-79	177.1047698
4.15E-57	124.3424257
7.29E-81	182.3271174
5.14E-78	175.1056625
1.77E-55	120.4845579
1.84E-65	144.3477556
5.56E-45	95.72209523
1.49E-59	130.1881689
1.29E-65	144.7212317
1.90E-61	134.7396507
1.03E-68	152.229543
1.66E-76	171.1729242
9.60E-68	149.9037572
6.28E-63	138.2812159
1.49E-64	142.1532006
6.86E-68	150.2629101

5.29E-55	119.3747797
1.68E-65	144.4486036
3.01E-53	115.2309245
5.14E-66	145.7003273
8.18E-48	102.3746417
1.18E-65	144.8128004
1.91E-74	166.2292495
1.42E-89	204.4858854
3.94E-66	145.9903333
3.81E-75	167.952452
1.09E-65	144.9116234
1.10E-79	179.1832426
5.08E-48	102.8638754
1.02E-57	125.8003306
6.75E-68	150.290243
3.62E-65	143.6374576
5.27E-51	109.8925319
7.72E-81	182.1645401
3.98E-59	129.1689215
2.44E-65	144.0518353
4.44E-47	100.6309137
4.03E-69	153.2368596
5.22E-70	155.4364904
3.85E-84	190.648512
2.59E-47	101.1839399
4.78E-80	180.0885436
3.39E-44	93.87033547
1.75E-52	113.3901515
1.38E-44	94.7861333
3.50E-70	155.8496169
5.10E-57	124.1306493
4.31E-48	103.0349564
7.31E-71	157.5556062
2.05E-62	137.0556048
3.22E-58	127.0074406
1.14E-54	118.590245
3.83E-71	158.2726434
9.09E-78	174.3839981
1.16E-63	140.0374949
1.03E-62	137.7699524
5.31E-68	150.5512754
2.33E-49	106.0235584
1.91E-69	154.0482439
1.49E-45	97.06966817
2.20E-55	120.2653918
9.56E-54	116.4033811
3.45E-61	134.1186402
6.92E-59	128.5909628
3.87E-45	96.09474299
3.28E-36	75.10319269
1.51E-64	142.1312655
1.48E-64	142.1643364
2.38E-64	141.6557643

3.46E-76	170.4042283
1.12E-68	152.1258727
3.55E-61	134.0843711
6.19E-50	107.3728642
1.01E-59	130.6002718
4.68E-58	126.6112026
2.79E-73	163.4786133
7.62E-73	162.428254
1.53E-45	97.03427927
1.90E-44	94.46107892
1.01E-58	128.1951217
2.21E-59	129.7785246
9.19E-49	104.6188475
3.71E-66	146.0590104
9.78E-43	90.43712868
1.80E-52	113.35787
4.51E-75	167.7536008
6.59E-70	155.1896652
1.37E-45	97.16085103
5.50E-67	148.0872506
1.02E-41	88.03651504
6.93E-66	145.3778833
2.65E-72	161.0720695
1.56E-56	122.968032
6.21E-49	105.0176497
1.58E-58	127.7342923
1.31E-69	154.4662376
1.10E-75	169.2202583
7.65E-60	130.8945489
3.72E-63	138.8191259
2.34E-40	84.84523992
7.73E-41	85.97397239
2.93E-81	183.3522092
5.32E-52	112.2427123
2.45E-37	77.75287665
1.13E-54	118.6003346
4.79E-34	70.02028183
4.19E-66	145.9197471
4.34E-25	49.05364493
7.86E-54	116.6064841
1.07E-39	83.29280093
7.67E-67	147.7465275
4.44E-52	112.4322414
4.59E-75	167.7049031
2.53E-52	113.0077421
4.86E-60	131.3592774
5.76E-68	150.4586846
2.48E-77	173.2943215
2.82E-74	165.8120209
2.85E-55	120.0051729
7.82E-51	109.4849994
1.05E-42	90.36182529
7.26E-22	41.5071765

8.26E-68	150.066846
1.50E-48	104.1102409
2.33E-45	96.61395274
1.61E-38	80.52952763
2.01E-38	80.30960981
8.12E-75	167.108756
2.26E-26	52.05762689
5.66E-63	138.3949933
1.76E-37	78.08890572
6.25E-50	107.359769
8.79E-58	125.9551539
1.82E-64	141.9305424
1.57E-32	66.47316915
1.12E-26	52.77579843
3.36E-72	160.7945125
5.66E-52	112.1753167
7.59E-51	109.5182295
3.68E-67	148.5077899
5.29E-53	114.642595
2.09E-35	73.2197396
8.51E-69	152.443792
3.26E-47	100.9488399
7.71E-50	107.1444322
2.78E-34	70.57302245
5.20E-45	95.79246607
2.30E-65	144.1187974
3.32E-63	138.9401939
1.49E-67	149.4380166
6.75E-65	142.9811655
2.04E-61	134.6608551
6.25E-50	107.3591063
1.73E-57	125.2469983
4.95E-24	46.57541827
9.13E-53	114.0692289
1.20E-43	92.5776483
2.54E-34	70.66566239
5.96E-45	95.65123474
3.82E-54	117.3427771
2.72E-57	124.7797658
1.47E-59	130.2062957
2.11E-72	161.3429684
4.05E-54	117.2815337
5.46E-44	93.38476331
1.40E-72	161.7766626
1.09E-65	144.8989083
1.62E-70	156.6474809
9.63E-57	123.4749929
6.83E-45	95.51064413
1.07E-50	109.1617094
1.23E-43	92.55500643
2.43E-62	136.8746536
2.40E-80	180.9382089
4.01E-45	96.05555086

3.40E-45	96.22743545
6.73E-34	69.67255079
4.73E-61	133.7664687
3.45E-61	134.1193866
3.20E-44	93.92908492
5.29E-46	98.12590905
2.65E-66	146.4091067
1.60E-73	164.0565437
6.25E-59	128.6966608
7.36E-71	157.5315154
6.17E-58	126.3174428
1.87E-34	70.97853049
4.84E-47	100.5436977
9.80E-49	104.5533939
8.83E-46	97.6029174
1.38E-83	189.1583758
3.53E-54	117.4237472
2.19E-66	146.6083882
1.83E-57	125.1865544
8.78E-53	114.1120511
9.51E-67	147.4944804
1.30E-70	156.9147058
1.18E-62	137.6263311
4.74E-39	81.77646695
9.69E-73	162.1648852
4.32E-72	160.5222
6.31E-69	152.7781668
5.44E-49	105.1532366
1.96E-63	139.480261
3.89E-80	180.3716218
9.92E-48	102.1776024
5.27E-39	81.66744329
5.20E-56	121.7347662
1.03E-58	128.17185
1.16E-51	111.439984
1.41E-69	154.3721564
8.60E-51	109.383449
5.61E-51	109.827402
6.62E-78	174.8001554
9.13E-70	154.8359047
1.10E-56	123.3402332
2.65E-50	108.2452789
1.00E-66	147.4148077
3.35E-85	193.7699018
2.82E-23	44.81019576
6.14E-59	128.7178724
1.02E-68	152.2519959
2.59E-47	101.1868986
9.65E-77	171.8817966
2.60E-81	183.6053325
7.64E-55	118.99985
3.40E-46	98.57152461
2.44E-62	136.8678336

5.87E-64	140.726475
1.53E-56	122.9895559
1.01E-55	121.0525106
1.80E-40	85.11371059
1.01E-76	171.7641467
9.35E-40	83.43474476
2.84E-59	129.521321
2.38E-64	141.6536703
2.46E-33	68.35845448
4.04E-33	67.85595807
2.20E-41	87.26066813
5.07E-56	121.7666942
1.11E-47	102.0597133
1.65E-30	61.73966686
4.04E-47	100.7317193
6.08E-64	140.6860566
1.63E-30	61.75273368
5.00E-57	124.1526698
3.13E-44	93.9523366
7.27E-53	114.3169292
1.01E-76	171.733365
3.16E-57	124.6227903
2.91E-30	61.16047257
9.84E-53	113.98927
6.66E-78	174.7429276
1.29E-61	135.1410712
4.42E-47	100.6367226
5.38E-43	91.04657709
6.94E-51	109.6114575
9.01E-67	147.5614617
5.44E-61	133.6176642
1.40E-36	75.97710825
5.10E-57	124.1281267
1.85E-54	118.093552
1.54E-58	127.7600096
1.72E-61	134.8390453
6.79E-30	60.30243307
2.04E-42	89.68380421
2.19E-34	70.81773195
3.56E-38	79.72072862
4.45E-64	141.0098222
7.75E-39	81.27524447
4.41E-60	131.463969
3.99E-32	65.5251361
6.09E-65	143.0911713
1.63E-41	87.56618143
4.21E-40	84.24463224
4.38E-59	129.0698661
7.16E-41	86.05448147
1.77E-52	113.3728999
6.72E-54	116.7653805
4.55E-41	86.51587137
9.60E-68	149.8964347

2.26E-69	153.8663683
4.69E-14	23.19763101
1.25E-56	123.2042247
9.70E-38	78.69213929
1.56E-67	149.384808
5.31E-45	95.77069074
1.17E-30	62.09028262
5.07E-56	121.7626593
1.97E-40	85.02180483
3.81E-53	114.9820467
9.06E-38	78.76250082
1.34E-56	123.1295322
2.87E-52	112.8780237
4.08E-45	96.03836599
1.09E-68	152.1632341
6.82E-28	55.61852151
4.47E-13	20.90559334
1.42E-48	104.1680639
1.58E-48	104.0523995
3.45E-49	105.6173175
9.13E-15	24.86092385
3.82E-33	67.91302497
3.80E-34	70.25554595
4.40E-72	160.4852693
1.04E-59	130.5631198
3.98E-61	133.9579073
1.91E-69	154.046372
1.14E-37	78.52326913
6.36E-25	48.66315532
9.57E-32	64.62956079
9.67E-53	114.0097929
6.41E-42	88.51145571
1.82E-47	101.5509987
4.70E-37	77.08839013
3.34E-33	68.04703526
3.64E-16	28.15567437
3.87E-40	84.33009103
7.26E-57	123.7689554
3.24E-65	143.7553754
1.71E-47	101.6105409
9.88E-26	50.55803835
1.51E-29	59.48756897
4.25E-35	72.49223304
3.13E-38	79.85703163
4.99E-35	72.32555888
1.91E-20	38.18555876
1.46E-31	64.20188626
2.17E-38	80.23086332
1.05E-67	149.793799
4.25E-66	145.896233
2.68E-42	89.40821049
1.04E-24	48.16652711
2.70E-21	40.17555656

1.02E-43	92.74821835
1.41E-63	139.8170491
6.77E-44	93.16466858
8.16E-62	135.6155527
3.14E-30	61.08464877
9.18E-33	67.01923916
2.96E-47	101.0465194
4.43E-44	93.5934399
1.45E-47	101.7843743
4.30E-30	60.76552162
1.34E-53	116.0490609
1.86E-31	63.95492491
8.55E-62	135.5641569
7.96E-65	142.8102458
1.24E-47	101.9463126
3.39E-42	89.16269843
3.71E-65	143.5985245
1.35E-23	45.56067195
7.42E-57	123.7412797
3.62E-24	46.89556581
1.85E-54	118.0900473
3.18E-38	79.83907292
1.02E-32	66.90751857
7.85E-33	67.17806519
2.82E-42	89.35181535
5.22E-32	65.24768172
1.41E-63	139.813703
7.76E-52	111.8462342
3.92E-38	79.62074999
3.19E-21	40.00558161
1.10E-66	147.3102135
1.47E-70	156.7607249
1.47E-55	120.675798
3.18E-57	124.6131984
8.45E-60	130.7911802
3.18E-51	110.413628
1.85E-32	66.30583431
6.74E-38	79.06290652
6.13E-45	95.61935661
8.31E-36	74.1567963
1.22E-21	40.98275274
5.19E-23	44.18924818
4.09E-61	133.9235742
7.61E-51	109.5141622
7.67E-40	83.63368565
2.20E-58	127.3910867
3.94E-18	32.76212145
1.83E-37	78.05050517
1.17E-39	83.19950015
3.19E-60	131.790066
2.05E-47	101.4239672
1.31E-20	38.56868551
1.19E-42	90.23423017

9.91E-60	130.6193748
5.90E-29	58.09802247
1.13E-28	57.44417262
6.28E-52	112.0657892
3.50E-70	155.8592036
4.54E-59	129.0265091
2.51E-43	91.8262447
9.13E-70	154.8383054
9.59E-67	147.4673938
2.26E-52	113.1227081
4.59E-39	81.80904757
3.54E-21	39.89699431
8.80E-46	97.6071797
3.74E-22	42.17901087
6.49E-41	86.15446621
2.99E-33	68.16210216
6.02E-26	51.06068288
3.26E-51	110.387172
3.93E-52	112.5616012
1.26E-59	130.3665113
8.21E-46	97.68235344
2.27E-69	153.8345798
2.57E-25	49.58345914
8.92E-45	95.22976574
3.63E-28	56.25731049
4.75E-68	150.6740797
8.37E-53	114.1645955
1.64E-42	89.90378374
6.95E-53	114.3650608
7.40E-42	88.36477733
1.97E-52	113.2602052
2.73E-56	122.3951032
1.19E-30	62.07453047
3.81E-36	74.95271574
3.30E-25	49.33047608
3.54E-20	37.55502133
2.78E-56	122.3736249
1.33E-39	83.06960275
3.81E-31	63.22573937
7.38E-51	109.5489009
8.14E-45	95.3220091
1.69E-64	142.0111875
6.38E-42	88.51753644
1.53E-40	85.28276578
1.88E-22	42.88240136
1.61E-26	52.40402861
2.08E-28	56.82420137
7.42E-58	126.1267517
2.06E-43	92.02700602
3.76E-34	70.26675555
2.55E-37	77.71115741
3.37E-30	61.01188354
2.87E-35	72.89594778

2.55E-29	58.9542657
6.84E-60	131.0151538
3.65E-40	84.38973894
2.80E-65	143.9074916
1.69E-59	130.0560276
9.55E-58	125.8682313
2.78E-31	63.5456437
1.81E-34	71.01361839
1.97E-47	101.4693575
8.31E-46	97.66589595
1.34E-57	125.5100441
5.60E-35	72.20925739
3.76E-62	136.418282
5.31E-28	55.87089483
1.14E-53	116.2213605
6.04E-42	88.57625498
9.59E-67	147.472362
5.30E-38	79.31185959
3.95E-29	58.5091533
2.87E-42	89.3343334
6.26E-82	185.1749428
2.01E-66	146.7024964
2.51E-49	105.9455142
1.14E-50	109.0985997
4.75E-19	34.91621512
1.07E-58	128.1338946
8.10E-59	128.4310056
3.31E-45	96.25549235
6.80E-38	79.05255232
6.04E-42	88.57394527
3.63E-40	84.40067656
2.40E-62	136.8939663
3.10E-41	86.90919296
5.94E-18	32.34345432
2.65E-56	122.4286441
1.01E-52	113.9566319
2.89E-32	65.85023216
2.10E-34	70.86078195
5.98E-38	79.18398541
4.52E-35	72.43026204
1.14E-41	87.92687913
2.29E-48	103.6774013
2.90E-28	56.48566302
8.36E-44	92.94777108
3.09E-17	30.66369359
2.72E-64	141.5125959
5.17E-41	86.38275706
6.37E-11	15.85085004
5.91E-38	79.19651283
8.13E-48	102.3825187
6.51E-23	43.95905711
8.35E-28	55.41038991
1.05E-16	29.42197728

5.90E-61	133.5219206
1.29E-48	104.2707527
4.52E-38	79.47475404
2.87E-29	58.83366032
2.59E-41	87.09175813
1.02E-39	83.34711472
4.64E-35	72.40346567
2.89E-55	119.9882156
7.22E-41	86.04516273
9.51E-35	71.67124505
9.24E-20	36.58424952
4.21E-50	107.7728863
7.08E-30	60.25989259
1.34E-41	87.76022375
1.12E-31	64.46896494
1.79E-28	56.97451379
1.07E-31	64.51120138
1.45E-52	113.5841744
9.80E-48	102.1911373
2.76E-54	117.6805155
1.09E-22	43.43130455
2.42E-61	134.481946
2.59E-40	84.74059541
3.64E-40	84.39388338
1.65E-35	73.45950194
5.51E-45	95.73261407
9.88E-32	64.59770639
4.40E-50	107.7208133
4.58E-34	70.06571586
7.22E-41	86.04384702
3.84E-44	93.73984587
2.16E-42	89.628031
2.27E-35	73.1385337
3.17E-54	117.5339261
3.19E-49	105.6983948
9.53E-55	118.7739731
2.06E-43	92.03098768
1.68E-26	52.36610069
1.03E-25	50.51967301
1.77E-13	21.84671139
3.01E-51	110.4706556
1.70E-35	73.43118279
2.66E-30	61.25447365
8.71E-63	137.9433972
3.05E-29	58.77081327
2.18E-46	99.02307293
4.19E-39	81.90066903
9.20E-46	97.56041298
3.98E-32	65.52831715
1.05E-51	111.5352289
2.87E-49	105.8058647
7.42E-30	60.21026855
1.33E-21	40.89128141

4.10E-21	39.74780776
2.51E-36	75.37615336
2.06E-37	77.9278861
1.08E-18	34.07291899
1.68E-18	33.62801952
6.90E-20	36.87926781
4.17E-58	126.7396754
1.36E-34	71.30620707
3.57E-29	58.61005433
2.48E-41	87.13790043
4.09E-28	56.13896686
2.81E-63	139.112416
1.25E-42	90.18077933
8.29E-46	97.6699975
7.18E-37	76.65887111
7.37E-21	39.15095296
1.18E-21	41.01529935
4.38E-19	34.99777248
7.86E-27	53.12891501
3.00E-25	49.42831968
4.86E-48	102.909164
7.15E-28	55.56977628
4.55E-15	25.57605638
4.16E-53	114.8925728
2.00E-29	59.20061391
9.01E-67	147.5612833
3.63E-58	126.886053
1.13E-23	45.73388041
3.42E-16	28.21922125
1.83E-28	56.95351759
3.10E-35	72.81836029
3.63E-44	93.79804125
8.06E-31	62.46418191
9.24E-65	142.6552131
8.30E-32	64.7777443
4.34E-40	84.21245797
2.42E-20	37.94214448
1.43E-34	71.25508748
1.63E-41	87.55969232
3.68E-51	110.261612
3.19E-20	37.66343398
1.80E-52	113.3537817
1.45E-30	61.87103888
3.43E-53	115.0937426
1.04E-46	99.77138151
2.69E-23	44.85885737
7.28E-41	86.03501593
2.23E-59	129.76537
1.21E-34	71.42499686
4.06E-32	65.5079209
1.13E-45	97.3524929
5.15E-27	53.55700344
3.22E-39	82.17299186

3.40E-47	100.9044698
5.73E-34	69.83631178
4.57E-41	86.51045201
1.02E-47	102.1477061
2.21E-21	40.38088718
2.08E-39	82.61805892
1.05E-24	48.15530596
2.12E-29	59.143157
2.01E-38	80.30954045
5.10E-48	102.8580122
4.02E-23	44.44885065
4.71E-20	37.26533627
1.09E-58	128.1139619
4.22E-16	28.00327571
5.26E-41	86.36474787
9.96E-54	116.3549014
3.88E-59	129.2001055
6.14E-25	48.69978599
1.26E-17	31.57986575
1.15E-35	73.82942467
2.65E-32	65.93850379
1.35E-18	33.85008956
4.69E-54	117.1328611
1.13E-48	104.40279
3.14E-21	40.02093844
1.20E-32	66.74420723
4.11E-71	158.1653122
3.81E-35	72.60509268
1.04E-37	78.62004314
3.08E-33	68.12965706
1.61E-55	120.5785263
5.16E-16	27.79562045
2.09E-26	52.14266397
2.03E-13	21.70799801
8.01E-45	95.33950388
2.35E-34	70.74568827
9.90E-37	76.33024736
1.06E-24	48.14713033
1.96E-40	85.02422794
2.29E-37	77.82011119
1.07E-57	125.7478491
2.04E-22	42.7959889
2.45E-39	82.44655839
1.41E-63	139.8131827
4.86E-55	119.4639573
7.41E-35	71.92633126
1.61E-27	54.74191516
4.26E-15	25.6433432
1.48E-15	26.72184684
1.46E-48	104.1358422
2.53E-57	124.8588688
4.67E-35	72.3952101
1.21E-32	66.73901675

1.28E-43	92.51357054
5.45E-23	44.13862159
1.00E-52	113.9709663
8.07E-19	34.37432925
2.78E-33	68.23512108
1.38E-37	78.33834218
9.28E-20	36.57981515
6.31E-34	69.73850965
3.67E-24	46.87972275
1.02E-47	102.1424858
3.65E-26	51.56701916
3.65E-24	46.88393727
2.56E-36	75.35376155
1.04E-36	76.28090627
1.36E-13	22.11074055
7.15E-56	121.4098047
2.92E-36	75.22239473
2.65E-21	40.1922929
3.66E-32	65.61209145
1.65E-58	127.6851978
1.45E-12	19.70404972
1.53E-30	61.81854837
4.03E-15	25.69942485
1.14E-19	36.36557497
8.39E-43	90.592756
1.61E-52	113.4746807
9.71E-28	55.25722054
1.23E-21	40.97021821
7.58E-32	64.87050517
6.87E-35	72.00256456
2.41E-24	47.30747114
5.01E-33	67.6356771
2.99E-23	44.75206339
3.42E-46	98.56480942
9.89E-35	71.63129798
5.15E-37	76.99480275
3.03E-46	98.68894426
9.09E-26	50.64103874
1.85E-31	63.96095347
7.63E-19	34.43144367
2.94E-42	89.30728199
1.92E-33	68.61062794
2.68E-66	146.390648
4.64E-23	44.3048917
9.51E-50	106.926613
6.23E-36	74.44813639
1.29E-31	64.32754002
1.46E-51	111.2048556
3.56E-22	42.23043662
2.91E-42	89.31831034
7.94E-15	25.00257198
6.48E-20	36.94290915
2.63E-18	33.171853

4.35E-50	107.7357695
4.24E-24	46.73074835
9.80E-56	121.0839453
9.10E-15	24.86398786
5.45E-23	44.13876058
1.69E-24	47.66583007
1.27E-14	24.52169449
2.27E-32	66.09623095
2.27E-32	66.09997156
2.91E-22	42.43808306
5.26E-49	105.1904172
1.68E-22	42.99430759
1.57E-19	36.03825754
1.77E-40	85.13143603
5.74E-61	133.5543964
6.52E-36	74.40097849
2.87E-49	105.8070811
1.38E-35	73.64905904
2.34E-44	94.2483267
3.30E-35	72.75115908
2.80E-20	37.79546001
1.73E-36	75.75598206
5.21E-25	48.86667546
2.30E-36	75.46674579
1.18E-19	36.33726904
6.82E-47	100.1997677
1.33E-29	59.61849305
4.85E-34	70.0041976
2.92E-48	103.426939
5.55E-44	93.36671712
1.56E-22	43.07491702
8.93E-44	92.87940927
1.47E-70	156.7619353
4.27E-51	110.1081392
5.39E-24	46.48958428
1.45E-26	52.51079712
2.11E-28	56.80989188
2.85E-42	89.34291696
3.25E-14	23.56942934
3.20E-24	47.01811376
3.32E-38	79.79489665
1.15E-19	36.36190085
3.10E-27	54.07554586
5.92E-39	81.5499314
1.31E-36	76.03779073
4.41E-15	25.60844251
6.90E-16	27.50050557
1.24E-36	76.09433959
9.42E-21	38.90038077
5.05E-23	44.21700756
2.99E-25	49.4309481
1.94E-21	40.51209394
8.46E-29	57.73476833

6.86E-09	11.08651573
5.01E-40	84.06783617
2.13E-30	61.48000536
3.33E-23	44.64069736
9.88E-38	78.67214274
6.34E-40	83.82625394
1.55E-45	97.02436103
2.18E-47	101.3631101
6.51E-63	138.2387329
6.42E-19	34.60839256
3.70E-65	143.6098146
3.05E-15	25.98434794
2.73E-40	84.68877816
3.25E-27	54.02488115
6.77E-09	11.10023932
2.18E-26	52.09854374
1.02E-10	15.37184582
2.73E-48	103.4962128
8.00E-54	116.5861464
3.40E-18	32.90962569
6.17E-18	32.30409193
2.02E-33	68.55832189
1.33E-52	113.6813314
1.72E-18	33.60363858
1.59E-51	111.1119876
1.53E-42	89.97732193
4.33E-40	84.21558529
7.87E-30	60.15149727
5.30E-08	9.003219717
4.91E-21	39.56550487
1.52E-21	40.76068099
6.55E-21	39.2716796
2.56E-49	105.9246129
6.88E-10	13.43039431
9.11E-47	99.90713819
7.15E-18	32.15430045
2.96E-31	63.48093718
4.27E-15	25.6402424
1.19E-25	50.36541755
8.06E-44	92.98560143
1.37E-42	90.08903515
5.21E-54	117.0258291
9.92E-49	104.5391235
1.72E-41	87.51031785
2.65E-31	63.597458
3.16E-11	16.56394045
8.47E-41	85.87887172
5.42E-15	25.39421121
1.11E-25	50.43950452
1.98E-47	101.4640192
1.69E-24	47.66825109
7.06E-14	22.78146572
1.52E-10	14.96669348

3.71E-10	14.05762903
1.23E-23	45.65184488
1.56E-27	54.77569799
5.20E-50	107.5543826
1.02E-38	80.99040274
8.45E-41	85.88247008
6.07E-40	83.87288005
2.67E-28	56.56971541
3.80E-23	44.50537303
2.99E-50	108.1208025
2.00E-20	38.13818393
1.65E-30	61.73719788
1.33E-26	52.59849036
1.45E-45	97.10027723
1.36E-54	118.4010507
7.56E-25	48.48910017
8.96E-32	64.69888552
7.12E-27	53.22937338
3.90E-43	91.38069501
3.59E-38	79.71297918
7.72E-53	114.2499289
7.07E-45	95.4731669
1.22E-38	80.81748302
5.06E-36	74.65858269
3.13E-44	93.95207249
1.66E-30	61.73204548
2.41E-38	80.12444446
8.24E-41	85.90929146
8.02E-37	76.54345515
7.22E-42	88.39044746
1.36E-51	111.2747429
6.40E-21	39.29591428
9.35E-39	81.08429012
2.29E-29	59.06199669
1.61E-44	94.62949808
7.69E-15	25.03576452
1.65E-21	40.67560475
5.60E-18	32.40260966
0.000194777	0.639171795
1.41E-24	47.85174638
1.77E-35	73.38790971
1.08E-42	90.33702103
6.26E-15	25.24863211
3.16E-39	82.19275722
6.52E-12	18.17235344
3.61E-19	35.19517481
3.27E-50	108.0299427
2.69E-39	82.35510803
5.54E-35	72.22158442
3.91E-31	63.19975392
5.20E-55	119.3936044
1.36E-45	97.16899838
4.83E-52	112.3418638

3.02E-60	131.8515716
3.56E-37	77.37158238
5.42E-08	8.98096246
1.61E-35	73.48696115
7.14E-45	95.46266069
4.89E-28	55.95538309
1.38E-17	31.48856532
1.73E-17	31.25548295
8.91E-28	55.34506896
4.68E-41	86.48351953
1.25E-14	24.5409986
1.56E-34	71.16790569
1.43E-30	61.88492729
5.22E-17	30.13091683
4.32E-50	107.7454059
8.25E-46	97.67596167
2.34E-43	91.89813019
2.39E-15	26.23459717
1.09E-22	43.43412732
1.45E-56	123.0517204
2.82E-51	110.5374391
9.02E-33	67.03675787
9.86E-20	36.51814399
5.89E-29	58.09965652
5.86E-33	67.47629808
3.98E-21	39.77863098
8.15E-38	78.86975448
5.67E-47	100.3843491
1.46E-47	101.7759702
1.48E-50	108.828566
8.91E-39	81.13275556
3.93E-39	81.96591791
1.90E-28	56.91421055
3.31E-27	54.00385501
3.13E-56	122.2537352
1.02E-52	113.9435698
2.08E-39	82.61732566
5.28E-40	84.01213881
6.81E-27	53.27431323
5.69E-49	105.1075484
6.30E-42	88.53052855
8.65E-52	111.7363393
1.80E-33	68.67871327
3.20E-20	37.6591364
1.49E-22	43.11706519
7.36E-45	95.42801446
9.92E-52	111.5981518
2.18E-59	129.7994848
3.21E-57	124.5998769
2.32E-35	73.1127466
2.12E-56	122.6545851
1.07E-48	104.4584833
2.87E-37	77.58797646

6.95E-49	104.9030866
5.87E-62	135.959025
6.89E-38	79.0393333
1.45E-52	113.5845816
2.63E-07	7.370101735
6.99E-52	111.9528168
7.35E-45	95.43093916
1.72E-18	33.60118584
5.60E-43	91.00548507
2.74E-43	91.73567258
3.18E-38	79.83998064
3.92E-29	58.51715575
1.39E-53	116.009577
4.68E-31	63.01574455
5.43E-61	133.6231133
5.10E-42	88.74626549
1.37E-60	132.6574743
1.10E-35	73.87356008
3.71E-16	28.13499026
4.05E-46	98.39406695
9.68E-54	116.3858128
2.85E-47	101.0871443
1.91E-49	106.2253137
3.43E-53	115.0935567
1.47E-44	94.71888799
3.00E-47	101.0325326
3.20E-20	37.65818572
1.34E-13	22.12758353
5.01E-51	109.9438005
9.29E-60	130.6885888
2.82E-40	84.65379073
2.02E-60	132.2637804
1.04E-41	88.0135527
8.35E-50	107.0612481
1.43E-23	45.50248194
2.59E-29	58.93996131
5.19E-38	79.33486782
1.15E-42	90.26749139
1.24E-38	80.79973944
3.28E-09	11.83453105
7.31E-53	114.3093025
5.09E-58	126.5198484
3.48E-46	98.54632726
2.18E-13	21.63676127
1.03E-32	66.89914256
7.56E-46	97.76654879
2.11E-46	99.05912694
1.20E-28	57.38189255
1.43E-50	108.8668944
6.37E-44	93.22825832
2.04E-56	122.6952369
4.84E-19	34.89599956
2.30E-46	98.9655234

1.69E-34	71.08465607
6.78E-62	135.8059235
5.85E-58	126.3736447
1.42E-39	82.99846188
6.06E-63	138.3220338
3.95E-49	105.480413
4.31E-58	126.7031902
4.96E-12	18.45081567
5.59E-53	114.5833292
8.76E-47	99.94830616
4.42E-61	133.8383712
1.03E-64	142.5385406
4.02E-44	93.69192924
4.07E-24	46.77284937
2.21E-61	134.576851
7.04E-44	93.12381957
4.28E-67	148.3466766
2.21E-26	52.08125353
1.50E-29	59.49463042
1.41E-57	125.4552481
2.27E-36	75.47889501
8.90E-37	76.43917253
9.81E-59	128.2360243
1.32E-28	57.28522836
1.68E-46	99.28630457
3.42E-59	129.3284962
1.01E-25	50.53806559
8.64E-55	118.8746564
1.78E-25	49.96131459
1.97E-37	77.97432136
1.48E-56	123.0270144
4.35E-50	107.737423
2.60E-37	77.69065873
9.11E-47	99.90665664
1.64E-22	43.02332231
5.69E-38	79.23700812
4.68E-26	51.31524006
4.49E-48	102.9893435
5.94E-56	121.5997855
3.44E-27	53.96609298
1.29E-36	76.05441805
1.05E-21	41.13495474
4.47E-34	70.09125553
3.95E-61	133.9698475
1.26E-54	118.483816
9.36E-20	36.56980379
1.28E-19	36.24694424
8.83E-31	62.37123362
3.18E-52	112.7733524
1.33E-34	71.33015264
1.02E-47	102.149701
3.78E-61	134.0190565
1.08E-33	69.19783627

1.43E-52	113.5991626
9.16E-45	95.20205085
2.23E-22	42.70714069
5.24E-54	117.0171532
1.32E-53	116.0645459
2.52E-44	94.17381163
1.55E-43	92.31543163
3.47E-53	115.0787971
1.30E-60	132.7179138
4.29E-26	51.40328446
1.26E-57	125.57569
3.50E-20	37.56698857
7.60E-66	145.2775797
4.75E-55	119.4888488
1.62E-34	71.1237805
7.39E-57	123.7481141
3.15E-27	54.05609371
3.04E-46	98.68498475
1.25E-58	127.9707714
2.99E-36	75.19788278
6.14E-50	107.3830378
8.04E-20	36.72537341
5.04E-58	126.5322385
3.04E-24	47.07185439
3.68E-24	46.87663267
1.39E-24	47.86697511
1.95E-39	82.68348862
4.25E-33	67.80335157
3.66E-31	63.26701082
2.24E-21	40.36739024
2.47E-40	84.79118669
2.37E-36	75.43452547
3.13E-67	148.6793084
6.79E-39	81.40845847
3.55E-38	79.72654721
1.25E-20	38.61281779
1.44E-27	54.85599036
2.83E-57	124.7350783
5.03E-52	112.2999706
5.05E-29	58.25807299
3.44E-36	75.05561213
3.90E-64	141.1465566
1.67E-20	38.31914414
1.84E-34	70.99644055
1.01E-58	128.2007146
1.00E-37	78.65637364
2.87E-35	72.89924019
7.48E-69	152.5962675
6.63E-26	50.96190907
2.27E-69	153.8445236
4.60E-38	79.45663079
9.59E-54	116.3983153
6.46E-56	121.5142078

3.03E-23	44.73628038
1.29E-64	142.3094598
7.03E-48	102.532379
1.44E-45	97.10789055
1.16E-32	66.77772854
2.43E-39	82.45788175
8.96E-61	133.0970311
5.56E-61	133.5904235
1.66E-35	73.4519502
2.96E-53	115.2499095
6.46E-62	135.8585812
2.80E-33	68.22581433
4.11E-71	158.1664227
3.66E-58	126.8725822
1.03E-42	90.38138467
6.36E-54	116.8228242
2.42E-41	87.16355255
3.26E-48	103.3138379
6.53E-32	65.02084611
4.27E-35	72.48843896
4.04E-25	49.12678913
2.20E-32	66.13215764
1.37E-42	90.09059795
7.17E-31	62.58396339
1.87E-40	85.07474551
9.43E-44	92.82391822
1.83E-46	99.19911799
5.25E-62	136.0756444
5.97E-45	95.64700856
1.25E-28	57.34092324
1.24E-40	85.49467437
