# Supplementary figures and images for: Uncovering the Immune Cell Infiltration Landscape in Low-Grade Glioma for Aiding Immunotherapy
Source: J Oncol. 2022 Mar 11;2022:3370727. doi: 10.1155/2022/3370727 (PMC8933094; doi:10.1155/2022/3370727)

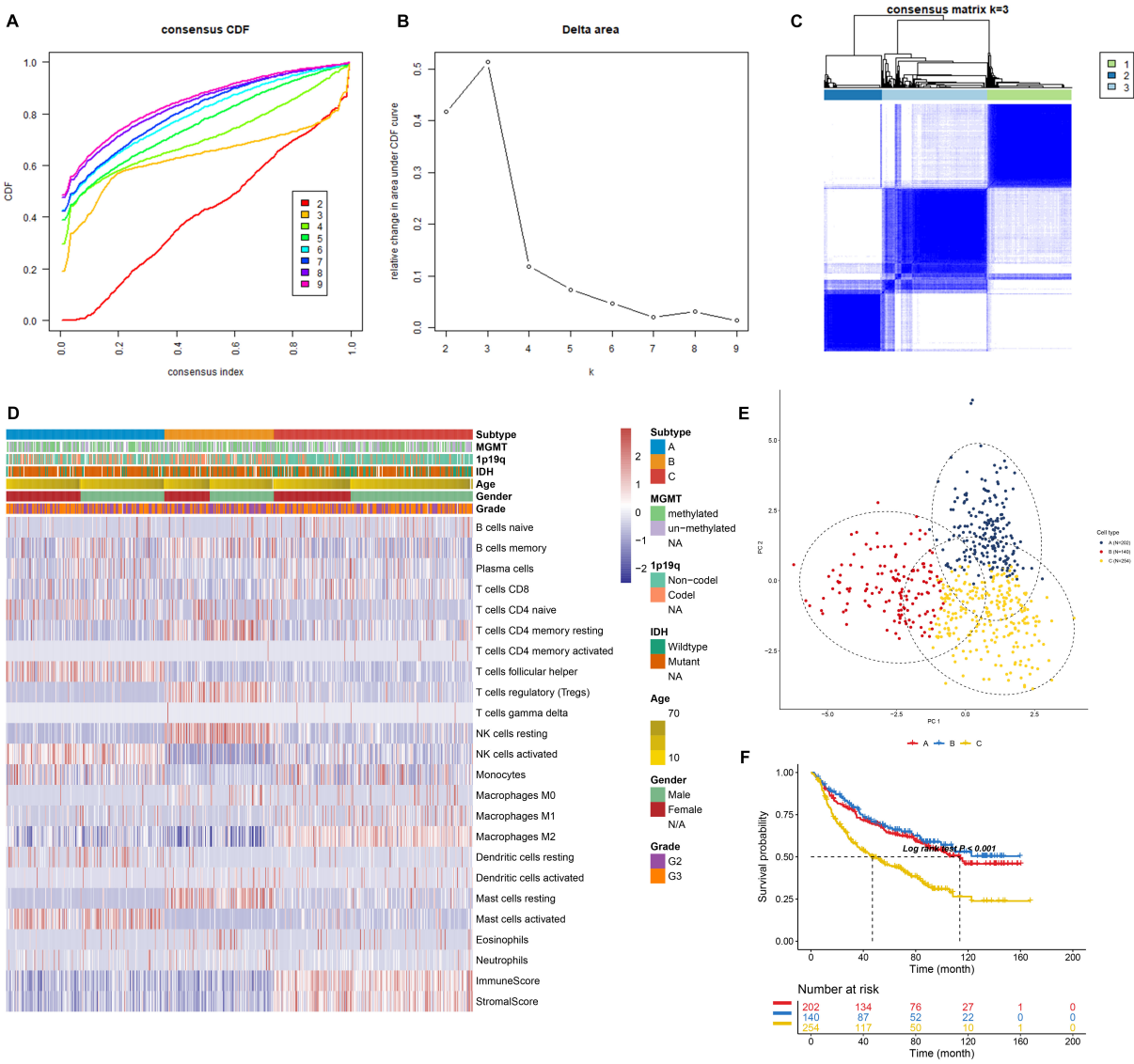

Supplement: Supplementary Materials — Supplementary Table 1: the list of ICI gene signatures A and B. Supplementary Table 2: GSEA results in high ICI score group. Supplementary Table 3: GSEA results in low ICI score group. Supplementary Table 4: DEGs between high and low ICI score groups. Supplementary Figure 1: validation of the three ICI subtypes for LGG in the CGGA-LGG dataset. (A–C) Unsupervised clustering analysis for validating the classifications of three ICI subtypes. (A) Consensus cumulative distribution function graph. (B) Delta area plot. (C) Heatmap for consensus matrix when k = 3. (D) Heatmap of tumor-infiltrating immune cells in different clinical phenotypes and ICI subtypes. (E) PCA plots for confirming the classification patterns of the ICI subtypes. (F) Kaplan-Meier curves for OS of LGG patients in the three ICI subtypes. [file 3370727.f1.zip › 3370727.f1/Supplementary figure 1.pdf]
